# Computation-through-Dynamics Toolkit: Simulated datasets and quality metrics for dynamical models of neural activity

**DOI:** 10.1371/journal.pcbi.1014755

**Published:** 2026-10-15

**Authors:** Christopher Versteeg, Jonathan D. McCart, Mitchell Ostrow, David M. Zoltowski, Clayton B. Washington, Laura Driscoll, Olivier Codol, Jonathan A. Michaels, Scott W. Linderman, David Sussillo, Chethan Pandarinath

**Affiliations:** 1 Wallace H. Coulter Department of Biomedical Engineering, Emory University and Georgia Institute of Technology, Atlanta, Georgia, United States of America; 2 Center for Machine Learning, Georgia Institute of Technology, Atlanta, Georgia, United States of America; 3 Department of Brain and Cognitive Sciences, Massachusetts Institute of Technology, Cambridge, Massachusetts, United States of America; 4 Wu Tsai Neurosciences Institute, Stanford University, Stanford, California, United States of America; 5 Department of Statistics, Stanford University, Stanford, California, United States of America; 6 Allen Institute for Neural Dynamics, Seattle, Washington, United States of America; 7 Department of Neurobiology & Biophysics, University of Washington, Seattle, Washington, United States of America; 8 Département de Neurosciences, Faculté de Médecine, Université de Montréal, Montréal, Canada; 9 MILA, Quebec Artificial Intelligence Institute, Montréal, Quebec, Canada; 10 School of Kinesiology and Health Science, Faculty of Health, York University, Toronto, Ontario, Canada; 11 Department of Electrical Engineering, Stanford University, Stanford, California, United States of America; 12 Department of Neurosurgery, Emory University, Atlanta, Georgia, United States of America; Research Center Jülich, GERMANY

## Abstract

A primary goal of systems neuroscience is to discover how ensembles of neurons transform inputs into goal-directed behavior, a process known as neural computation. A powerful framework for understanding neural computation uses neural dynamics – the rules that govern how neural activity evolves over time – to explain how goal-directed input-output transformations occur. As dynamical rules are not directly observable, we need computational models that can infer neural dynamics from recorded neural activity. We typically validate such models using synthetic datasets with known ground-truth dynamics, but unfortunately existing synthetic datasets don’t reflect fundamental features of neural computation and may therefore be poor proxies for neural systems. Further, the field lacks validated metrics for quantifying the accuracy of the dynamics inferred by models. The Computation-through-Dynamics Toolkit (CtDToolkit) addresses these critical gaps by providing: 1) synthetic datasets that reflect computational properties of biological neural circuits, 2) interpretable metrics for quantifying model performance, and 3) a standardized pipeline for training and evaluating models with or without known external inputs. In this manuscript, we demonstrate how CtDToolkit can help guide the development, tuning, and troubleshooting of neural dynamics models. In summary, CtDToolkit provides a necessary framework for model developers to better understand and characterize neural computation through the lens of dynamics.

## Author summary

Understanding how the brain works requires interpretable accounts of how populations of neurons process information to produce behavior. One powerful approach is to study “neural dynamics,” the patterns of how neural activity evolves over time. Scientists develop computational models to infer these dynamics from neural recordings, but it has been challenging to know when the inferred dynamics are trustworthy. Existing datasets often lack key features of biological neural circuits, and current performance metrics can provide an incomplete picture of model quality.

We developed the Computation-through-Dynamics Toolkit (CtDToolkit) to solve these problems. Our toolkit provides three key resources: biologically motivated synthetic datasets, improved metrics that provide more holistic accounts of model performance, and a standardized workflow for training and evaluating models. We hope that CtDToolkit enables researchers to rigorously test, improve, and troubleshoot their models before applying them to real brain data. This work establishes a crucial foundation for developing better methods to understand neural computation, ultimately advancing our ability to decode how the brain transforms sensory information into thought and action.

## 1 Introduction

### 1.1 What is the Computation-through-Dynamics Toolkit?

Understanding how the brain performs computation is the central aim of systems neuroscience. Although modern neural interfaces can now monitor hundreds or thousands of neurons simultaneously [1], we struggle to translate these massive new datasets into interpretable accounts of neural computation. We need a conceptual language that can describe how neural populations transform inputs into goal-directed behavior. Neural dynamics - the rules that govern how neural activity evolves over time - have gained renewed attention through advances in artificial neural network research [2–5]. Neural dynamics offer a framework for connecting neural observations with neural computation [6].

A dynamics-based understanding of neural computation requires new methods that can estimate the (not directly observable) dynamics of neural circuits. Recent years have seen a surge in data-driven models that attempt to infer these dynamics by learning to reconstruct observed neural activity as the product of a model dynamical system [7–14]. Unfortunately, we lack consensus on the types of synthetic systems and performance criteria that are appropriate for evaluating these models. A common set of datasets and metrics for model evaluation would facilitate model comparisons and help promising innovations be disseminated more quickly through the field.

Notably, the broader field of dynamical systems reconstruction (DSR) has long grappled with the analogous problem of validating an inferred dynamical system against a known ground-truth system, and has developed a mature set of evaluation measures for doing so on canonical (non-neural) systems such as the Lorenz and Rössler attractors [15–17]. However, the additional challenges presented by the dynamics implementing neural computation, chiefly the presence of substantial unknown external inputs and goal-directed task structure, require a framework that links these well-established methods to biological computational systems.

In this manuscript, we introduce the Computation-through-Dynamics Toolkit (CtDToolkit), a model development platform designed to help researchers assess the strengths and weaknesses of different data-driven (DD) dynamics models across a range of simulated neural datasets. As part of CtDToolkit, we present (1) a library of synthetic datasets that reflect goal-directed dynamical computations, (2) performance criteria and metrics designed to reveal specific model failures, and (3) a public codebase that allows researchers to submit new models, generate customized synthetic datasets, and iterate rapidly during model development. CtDToolkit is designed for modularity and extensibility, serving both as a shared framework for comparing DD models and as a flexible platform for tailoring datasets to specific experimental contexts.

This manuscript is divided into three sections: Section 1.2 lays out a theoretical foundation for the synthetic systems and performance metrics included in CtDToolkit, which Section 2 then describes in detail. Section 3 provides illustrative examples of how CtDToolkit datasets and metrics can help guide DD model development.

### 1.2 Computation-through-Dynamics: Definitions, approach, and challenges

#### 1.2.1 Problem definition.

Any satisfying account of neural computation needs to span three conceptual levels [18]: computational, algorithmic, and implementation (Fig 1A). In this section, we provide a high-level description of each level and illustrative examples from a simple computational system, the 1-bit flip-flop.

**Fig 1:**
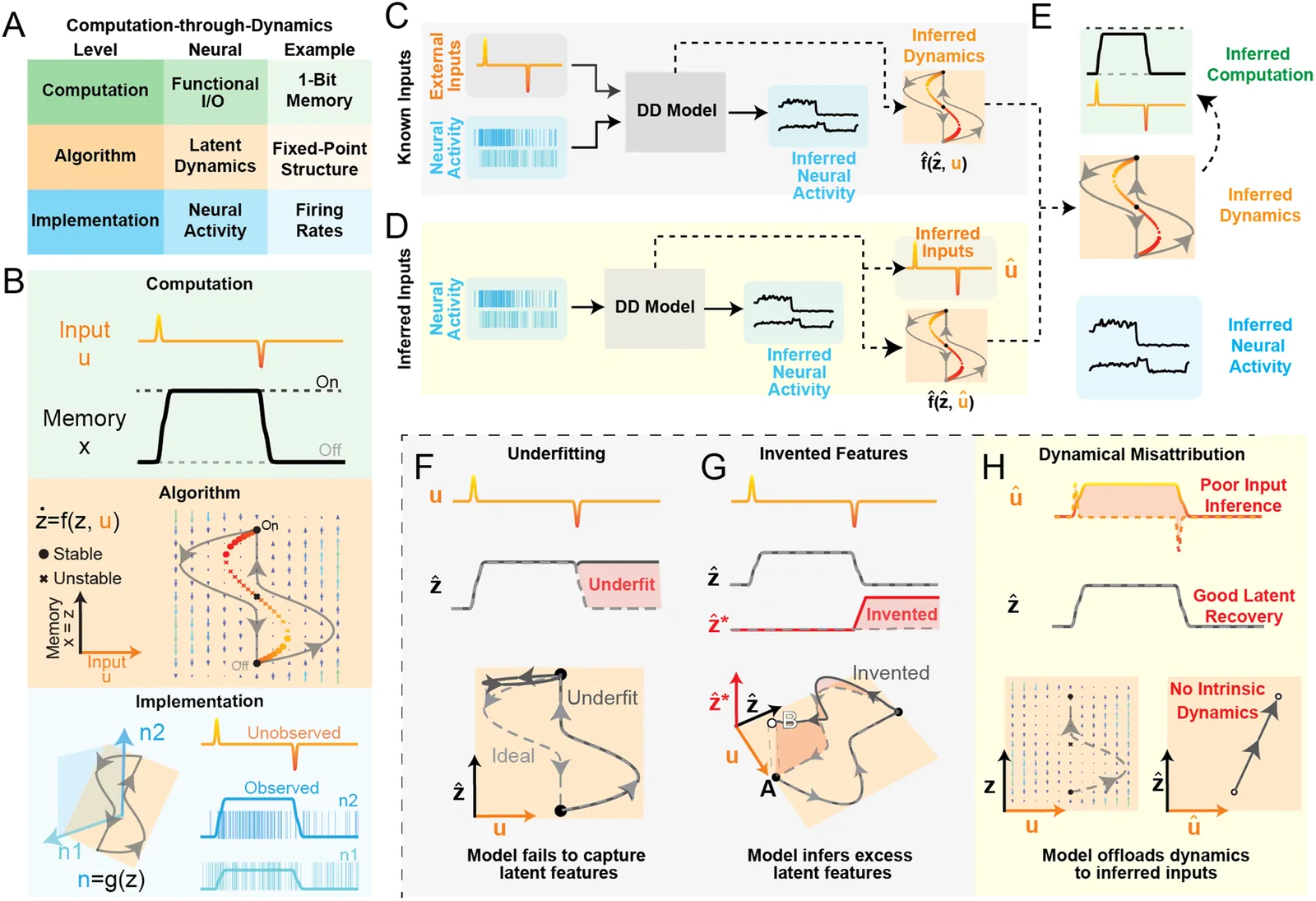
Computation-through-Dynamics framework and data-driven model failure modes. **A)** Overview of Computation-through-Dynamics framework with levels, corresponding concepts in neuroscience, and specific examples from 1-Bit Memory (1BFF) task. **B)** Schematic of conceptual hierarchy of 1-Bit Memory task. Top row (green): Computation – Example of input pulses **u** and desired output p for a single trial of 1BFF task. Middle row (orange): Algorithm – State-space diagram of dynamical system that performs 1BFF. x-axis: external input **u**. y-axis: latent state **z**. Flow field (written as **f**) controls how **z** evolves as a function of both **z** and **u**. circles: stable fixed points,: unstable fixed points. Output **x** is generated by projection **h** acting on the latent **z**. In this example **h** is the identity function. Bottom row (blue): Implementation of latent dynamics in neural activity. Left: Schematic of simple linear observation model of 1D latent dynamics into 2D neural activity. Inputs **u** are orthogonal to the linear observation model, implying that inputs are not directly visible in neural activity. Right: Simulated firing rates **n** and spiking activity **y** for two neurons in blue. **C)** Schematic of data-training pipeline (with known external inputs). Solid lines represent inputs and outputs from the DD model, dashed black lines represent what we hope our DD models infer indirectly. Neural observations (blue) and external inputs (orange) are provided to the dynamics model, which is optimized to reconstruct neural activity. **D)** Data-training pipeline (with inferred external inputs): Same as C, except the DD model does not receive external inputs and is instead tasked with inferring them. **E)** Our goal is to infer the computation performed by a neural circuit, but we only have access to observed neural activity. We hope that the dynamics $\hat{𝐟}$ inferred by models in panels C,D match the true dynamics **f**, and will provide the basis for us to infer the computation performed by the circuit (green). **F-G)** Schematics of failure modes for models fit to 1BFF with supplied external inputs. **F)** Underfitting – Top row: True inputs **u** (orange/red) and true/inferred latent activity **z**/ $\hat{𝐳}$ (solid/dashed, respectively). Red shaded area shows the activity that the underfit model fails to capture. Bottom row: State-space diagram of an ideal DD model (gray dashed line) and underfit DD model (black solid line). **G)** Invented Features – Top row: Same as F, except DD model has extra latent dimension ${\hat{𝐳}}^{*}$, highlighted by a red shaded area. Bottom row: 3D state-space diagram of ideal DD model (gray dashed line) and DD model with invented features (solid black line). Ideal model and invented features model share fixed points (filled black circles, “A”), but the invented features model has an additional “invented” fixed point (white circle, “B”). This panel is a simplified, conceptual illustration of what an invented feature can look like for a short input sequence (here, a single pair of input pulses), and is not intended to depict the full dynamics under prolonged or repeated inputs. **H)** Schematic of the dynamical misattribution failure mode, which affects models that infer external inputs. Top row: Model-inferred inputs do not match the true inputs (highlighted by red shaded area), but accurately capture the latent activity without inventing features. Bottom row: State-space diagram with flow field for ideal DD model (left) and DD model with poor input accuracy (right). In this schematic, we only show the rising bit-flip for visual clarity. With inferred inputs, DD models are not obligated to learn any intrinsic dynamics.

**What do we mean by “neural computation”?** The computation level is concerned with the goal a system is trying to accomplish. Because brains evolved to generate adaptive behavior, it is impossible to fully understand a neural circuit without reference to a computational goal. We loosely formalize a neural computation as a mapping from inputs **u** to outputs **x** tuned to accomplish a specific behaviorally-relevant goal (e.g., memory, sensory integration, control). Importantly, these inputs and outputs do not necessarily relate exclusively to the external world: they can include communication with other brain areas (e.g., internal models, planning activity).

To make this concrete, consider the 1-bit flip-flop (1BFF) computation (Fig 1B, Computation): the output should reflect the sign of the most recent input pulse. Crucially, a computation specifies *what* must be done, but not *how*.

**How are algorithms built by neural dynamics?** We define an algorithm as a set of rules that, when followed, enact a particular computation. In the CtD framework, these algorithmic rules are built from the neural dynamics of a circuit. Formally, neuronal circuits learn a *D*-dimensional latent dynamical system $\dot{z}=𝐟(𝐳,𝐮)$ and corresponding output projection **x** = **h**(**z**) whose time-evolution approximates the input/output mapping 𝐮↦𝐱.

For the 1BFF computation, we show that a relatively simple dynamical system **f** with output projection **h** (in this case, the identity function) can accomplish the desired mapping via an input-dependent flow field (Fig 1B, Algorithm). Sufficiently large input pulses carry the state over a saddle point, which mediates the transition to a new attractor state.

**How are these dynamics implemented in neural activity?** In the brain, neural dynamics arise from the underlying biophysics of neural circuits, which in turn arises from the physical properties of individual neurons, such as their ion channels, synapses, neuromodulators, and cell types. Yet the dimensionality of neural population activity is typically far lower than the total number of neurons, suggesting that neural dynamics can instead be modeled by a low-dimensional dynamical system embedded in a high-dimensional neural activity space (N≫D) [19,20]. We denote the deterministic mapping from latent activity to neural firing rates as $𝐧=𝐠(𝐳):{\mathbb{R}}^{D}\to {\mathbb{R}}^{N}$ and refer to **g** as the (true) rate observation model [20–22]. Observed neural spike counts **y** are subsequently generated from a conditional observation distribution p(𝐲∣𝐧).

For the 1-bit memory system, we implemented **g** as a simple linear-exponential transformation of the one-dimensional latent state into two-dimensional neural rates **n**, then sampled neural spike counts **y** from an inhomogeneous Poisson process with those rates (Fig 1B, Implementation). Importantly, external inputs **u** are typically unobservable; they must be inferred from their effects on observable neural activity **y**.

#### 1.2.2 Climbing levels: From neurons to dynamics to computation.

**Data-driven modeling:** While an interpretable account of a neural circuit spans all three levels, we typically have direct access only to the implementation level – the recorded neural activity **y**. We need methods that can accurately infer algorithmic features – dynamics **f**, observation model **g**, latent activity **z** from these neural observations (Fig 1C and 1D, with supplied/inferred external inputs respectively). We use $\hat{\cdot }$ to denote model-inferred features (e.g., inferred dynamics $\hat{𝐟}$ versus ground-truth **f**). Models that can be trusted to infer dynamics accurately (i.e., $\hat{f}≃f$) could allow us to climb from implementation (neural activity) to algorithm (neural dynamics), and then, hopefully, from algorithm to computation (Fig 1E).

In recent years, a new class of “data-driven” (DD) models has emerged that are trained to reconstruct recorded neural activity as the product of inferred low-dimensional dynamics and observation models ($\hat{𝐟}$ and $\hat{𝐠}$, respectively) [7–13,22–29]. DD models are typically evaluated by how well they reconstruct simulated neural activity from synthetic systems with known ground-truth dynamics **f**. However, reconstruction accuracy can be a poor proxy for how faithfully a model captures the underlying dynamics, and commonly used synthetic systems lack many features fundamental to real neural circuits [14,23,30]. Moreover, the absence of a shared language of synthetic systems and performance criteria makes it difficult to compare published models or test new architectures systematically.

The primary goal of CtDToolkit is to provide a common, theoretically grounded foundation for model development and validation. It allows researchers both to benchmark models on standardized synthetic systems and to design customized datasets that mirror the structure, inputs, or feedback properties of their own experimental recordings, enabling targeted model development for specific neural systems. Together, these capabilities will accelerate progress toward DD models that can accurately and reliably infer neural dynamics from biological data.

**Synthetic systems as proxies for neural circuits:** Following the broader dynamical systems reconstruction (DSR) literature, many neural dynamics models have been validated on well-characterized dynamical systems such as the Lorenz and Arneodo systems [7,8,10,11,13,14,23,31,32]. These systems provide valuable benchmarks for generic dynamics reconstruction: their governing equations and ground-truth states are known, their nonlinear dynamics can present substantial modeling challenges, and their relatively compact state spaces facilitate visualization and interpretation.

However, many canonical DSR benchmarks differ from neural circuits in two respects that are particularly important for studying neural computation. First, they are autonomous or receive only limited external input, whereas neural activity reflects both intrinsic dynamics and inputs from the environment and other brain areas. Second, they are not constructed to implement a specified, goal-directed input–output transformation. Consequently, although these systems can test whether a model reconstructs generic nonlinear dynamics, they do not directly test whether it can distinguish input-driven activity from intrinsic dynamics or recover dynamics that implement a known computation.

Chaos and dimensionality are separate axes of variation: both task-performing and non-task-performing systems can, in principle, exhibit regular or chaotic dynamics and occupy low- or high-dimensional state spaces. We therefore view task-trained benchmarks as a complement to, rather than a replacement for, canonical DSR systems. By training dynamical systems to perform specified tasks, CtDToolkit introduces known computations and controlled external inputs while retaining full access to the ground-truth dynamics. The included tasks span a range of dynamical regimes, including the near-chaotic dynamics of ChaoticDelayedMatching.

Importantly, the same observed neural activity could in principle be generated by many different underlying assumptions–-for example, about the form of inputs, the structure of feedback, or the dimensionality of latent dynamics. As a result, the choices made when simulating a dataset can dramatically affect the DD models that are best at capturing the ground-truth dynamics. DD model developers must take care to select or construct synthetic datasets whose properties best match those of the biological data to which they ultimately wish to apply their models. To enable this, our framework is designed to incorporate new datasets tailored to particular experimental needs, ensuring that validation exercises remain relevant and informative for the systems under study.

In Section 2.3, we describe how we obtain proxy systems with these properties by training dynamics models to perform specific tasks; we call these models “task-trained” (TT) models to distinguish them from the “data-driven” (DD) models that are trained to reconstruct neural activity.

**Performance criteria:** DD models are typically trained to reconstruct recorded neural activity, but recent work has shown that even near-perfect reconstruction does not imply that inferred dynamics are an accurate estimate of the underlying system (i.e., $\hat{𝐧}≃𝐧$ does not imply $\hat{𝐟}≃𝐟$) [32–34]. To address this problem, we have identified three key performance criteria that can collectively provide a more holistic assessment of DD model performance: *reconstruction*, *simplicity*, and *input accuracy*.

In this section, we provide intuition for the role of these criteria in model selection, while in Section 2.4, we provide formal definitions of specific metrics for quantifying model performance on each criterion.

***Reconstruction:*** The first performance criterion is reconstruction, or the extent to which a model can re-generate neural activity from trials held-out from the training set. We note that this definition may differ from its usage in the broader field of dynamical systems identification, where it broadly refers to parity between the inferred and ground-truth systems. Poor reconstruction can be a symptom of an important mode of model failure called underfitting. Underfitting in this context refers to a model failing to capture the latent features **z** that underlie the observed neural activity patterns **y**, often because the model lacks computational capacity, is over-regularized, or has had insufficient time to train. In the last few years, reconstruction has improved dramatically as the field has moved from simple linear dynamics to more expressive model classes (e.g., stacked state-space models, transformers) [9,35–37].

In Fig 1F, we show an example of underfitting: a hypothetical DD model whose inferred latents $\hat{𝐳}$ accurately capture the true latent activity **z** for the positive bit-flip (0→1), but fail to capture it for the negative bit-flip (1→0).

***Simplicity:*** The second performance criterion is simplicity, with an associated failure mode of feature invention. Feature invention is the error-of-commission counterpart to underfitting’s error of omission: while underfit models miss real features, hallucinating models invent features that the true system lacks. Existing methods to quantify simplicity treat the latent activity **z** as the “feature” of interest, measuring how well the ground-truth system can predict the model-inferred latents $\hat{𝐳}$ [32]. In principle, the same criterion could instead compare the inferred dynamics as a whole, such as entire flow fields rather than latent activity alone [33]. Models with both optimal simplicity and optimal reconstruction have a 1:1 mapping between the model-inferred latent features and the features of the underlying system.

One particularly insidious form of feature invention occurs when the invented features actually lead to improvements in reconstruction on both training and validation datasets. When ranking DD models by their ability to reconstruct neural activity, it has been shown that models with invented features often *outperform* those without [14]. This suggests that not only is reconstruction insufficient for DD model selection, but relying on reconstruction alone may be actively misleading [33]. While some metrics to quantify model simplicity have been released [23,32], these methods require comparisons against the ground-truth system and therefore are not applicable to models trained on biological datasets. Of note, some attempts have been made to control invented features without reference to ground truth, e.g., by identifying the minimum-complexity regime in which independently trained models converge on consistent inferred features [33], or by constraining the model’s observation model $\hat{𝐠}$ to be injective [14].

We show an example of feature invention in Fig 1G. Though our hypothetical model perfectly captures the true latent activity **z**, it also infers an additional latent dimension ${\hat{𝐳}}^{*}$. This “invented” activity evolves such that when the bit turns off, the state returns to a location that is different from where it began the trial. Alone, reconstruction and simplicity can each be insufficient to judge DD model quality, but together they can provide interpretable accounts of DD model performance when external inputs are known.

***Input Accuracy:*** Now we consider the more realistic scenario in which the external inputs **u** are unknown and therefore must be inferred alongside the latent dynamics. The third performance criterion is input accuracy, or how well the model-inferred inputs $\hat{𝐮}$ match the true external inputs **u** to the system.

In Fig 1H, we show a hypothetical model that accurately infers the latent activity $\hat{𝐳}$ without inventing features, yet fails to capture the true dynamics **f**. Its inferred inputs $\hat{𝐮}$ can reconstruct the data without relying on any intrinsic dynamics, while producing exactly the same predictions as the true model. Since both models are equally supported by the evidence, they cannot be disambiguated from observations alone–-an instance of non-identifiability, the general problem in which distinct underlying systems produce indistinguishable observations. We call this particular failure mode dynamical misattribution. Because input accuracy cannot be evaluated on most biological datasets, this failure mode is particularly troubling: even when $\hat{𝐳}$ is accurate, $\hat{𝐟}$ can still be a poor estimate of the true **f**.

## 2 Methods: Overview of the Computation-through-Dynamics Toolkit

### 2.1 Key motivation

Our goal is to facilitate the development of data-driven (DD) models that can recover the unobserved latent activity **z**, dynamics **f**, observation model **g**, and external inputs **u** that underlie observable neural activity **y**. CtDToolkit supports this goal by addressing the two shortcomings described above–-the limitations of existing synthetic systems and the insufficiency of existing performance metrics–-within a single standardized, extensible codebase. Rather than crowning a single best model, it offers a structured process through which developers can iteratively test, refine, and tailor models to datasets with different properties, and share the resulting innovations with the community. Below, we walk through this process as three steps (Fig 2): selecting a task, training DD models, and comparing them against task-trained ground truth, with an optional path for generating new synthetic datasets.

**Fig 2:**
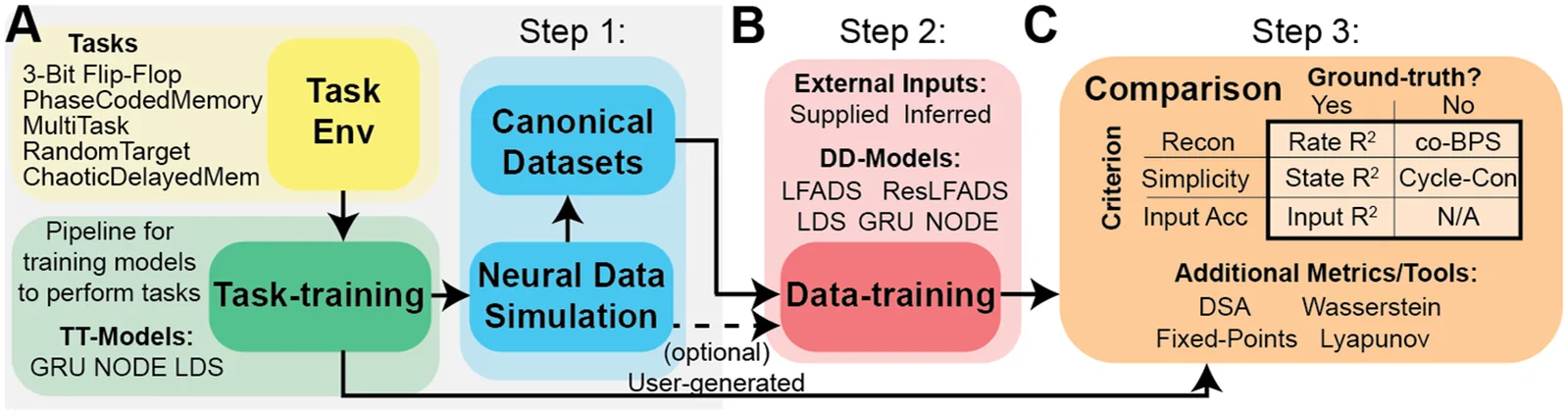
Features of the CtD Toolkit. **A**) Dataset generation pipeline- Task Environments (yellow) are taken as input to the task-training pipeline (green), which trains a dynamics model (available models: GRU, NODE, LDS) to perform the task. After training, the TT model is passed to a neural data simulator (cyan), which simulates spiking neural activity with configurable parameters. Users can either generate their own simulated datasets (dashed line) or use the pre-generated canonical datasets (solid line) **B)** Step 2: Data-training pipeline: Train a chosen DD model (available baseline models: LFADS, GRU, LDS, NODE) to reconstruct simulated neural activity, using either supplied or inferred inputs. Trained models are saved for analysis and comparison. **C**) Step 3: Model Comparisons- Top: Table of primary performance metrics, organized by criterion (rows), and need for ground-truth (columns). Bottom: Additional performance metrics/visualization tools.

### 2.2 Getting started with CtDToolkit

**Step 1: Select a task**: First, users must select a task to test their data-driven models.

At release, CtDToolkit provides pre-generated datasets for five tasks:

- *Three-Bit Flip-Flop*: Storing a 3-bit memory state that can be modified by external inputs.
- *MultiTask*: Performing a set of cognitive sub-tasks including category matching, sensory integration, etc.
- *RandomTarget*: Controlling the endpoint of a simplified biomechanical effector to instructed locations.
- *PhaseCodedMemory*: Remembering an input stimulus with reference to an input oscillation.
- *ChaoticDelayedMatching*: Comparing two sequentially-presented cues using near-chaotic dynamics.

Each canonical dataset includes everything needed for DD model training and evaluation, including simulated spiking activity and external inputs. We also provide the task-trained models from which the synthetic datasets were generated, allowing users to directly inspect the dynamics of the ground-truth system. Each dataset was chosen either to reflect a canonical property of biological neural circuits or to provide a simple, easily interpretable test case (Fig 2A); we expand on these choices in Section 2.3.

**Step 2: Train models on canonical datasets**: Within the CtDToolkit data-training pipeline, users can fit models on canonical datasets and easily perform large hyperparameter sweeps (Fig 2B). We support two training modes: supplied ground-truth external inputs or model-inferred external inputs. In addition to training user-defined models on synthetic data, users have access to a library of baseline models, including standard sequential auto-encoders and LFADS [10]. We define a common interface so that new models can be contributed to CtDToolkit, expanding the set of baseline models for future developers (S1 Text).

**Step 3: Compare task-trained and data-driven models**: CtDToolkit provides tools that facilitate the interpretation of task-trained and data-driven models, including metrics for quantifying performance on the criteria defined in Section 1.2.2. Some of the included metrics do not require ground truth and can be used to assess models trained on biological datasets (Fig 2C). CtDToolkit also provides methods for data handling, model inference, fixed-point finding, and visualizations that allow users to quickly and easily assess model performance and iterate during model development (Section 2.4).

**Optional: Generate new synthetic neural datasets**: We do not intend for any single synthetic dataset to serve as a definitive benchmark for all models. Instead, we envision researchers iteratively generating and refining synthetic datasets whose properties best match the biological data they aim to study. To support this process, CtDToolkit provides templates for custom tasks and TT model architectures that integrate with the existing task-training and data simulation pipelines. The simulation pipeline itself is configurable, enabling users to explore how design choices such as neuron count, noise model, or the true observation model **g** affect DD model performance (S1 Text) and evaluation by Toolkit metrics (Section 2.4).

### 2.3 Task environments

In contrast with previous validation datasets, CtDToolkit datasets were designed as biologically motivated synthetic benchmarks that capture selected challenges present in neural recordings while preserving full ground-truth access (see Section 1.2.2). These datasets were simulated from dynamical systems trained to perform specific tasks, defined in CtDToolkit as “task environments,” which provide simulated inputs and objective functions for quantifying TT model performance. Over the course of training, TT models learn dynamics that approximate the input/output mapping of the desired computation. For easy extensibility, task environment objects inherit from the Gymnasium Env class [38].

We include five task environments in CtDToolkit, chosen to provide a range in difficulty in 1) complexity of dynamics, 2) complexity of external inputs, and 3) present state of task interpretability. By *input complexity* we mean a qualitative descriptor of how amenable a task’s external inputs are to standard dynamical-systems analysis, rather than a formally defined scalar metric. We consider inputs to be *simpler* (i.e., lower complexity) when they are (i) piecewise constant over time and (ii) independent of the model’s outputs (open-loop). Piecewise constant inputs allow the dynamics to be analyzed under effectively stationary conditions within each epoch, making tools such as fixed point analysis directly applicable, whereas continuously varying or output-coupled inputs (e.g., in PhaseCodedMemory or RandomTarget) introduce time-dependent forcing that complicates interpretation of the intrinsic dynamics. We show the task schematics for 3BFF, MultiTask, and RandomTarget tasks in Fig 4, for the PhaseCodedMemory task in S4 Fig, and for the ChaoticDelayedMatching task in S5 Fig.

**Three-Bit Flip-Flop (3BFF)**: 3BFF is a 3-bit memory task with the goal of remembering the sign of the last input pulse on each of three noisy input channels (Fig 3A). We chose to include 3BFF in CtDToolkit because it has 1) simple dynamics, 2) simple inputs (Fig 3B), and 3) is well characterized by previous research [5]. TT models performing 3BFF typically exhibit low-dimensional activity organized into a cube, with stable fixed points at the vertices and unstable fixed points along the edges (Fig 3C).

**Fig 3:**
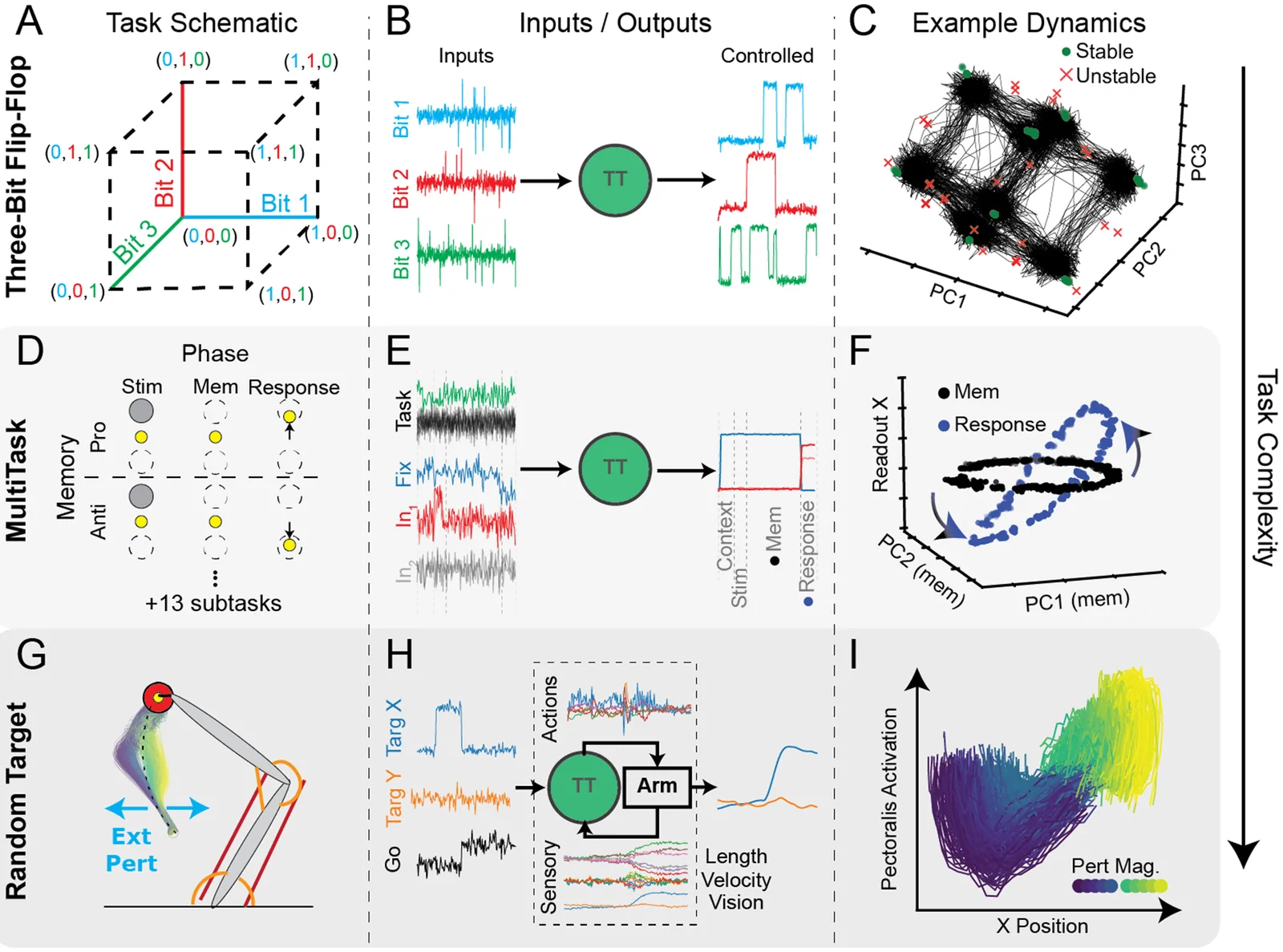
CtDToolkit Datasets have complex and interpretable dynamical features. **A**) Schematic of three-bit flip-flop (3BFF) task environment [5]. Three bits encoded according to inputs corresponding to 8 potential memory states. **B**) Inputs (left) and outputs (right) of the task-trained model for an example trial. **C**) Visualization of latent activity (black traces) and fixed points (FP) from canonical 3BFF TT network. Stable FPs (green circles) are found on vertices and unstable FPs (red ×s) along edges of the cube [39]. **D**) Schematic of 2 of 15 tasks in MultiTask environment (MemoryPro, MemoryAnti). Each task has different rules for how to generate outputs. For full details, see [40] **E**) Example of single trial inputs/outputs from canonical MultiTask TT-GRU performing “MemoryPro” task. Task phases indicated by dashed vertical lines. **F**) Example FP architectures during two phases of MemoryPro task (Mem1 (black) and Resp (blue)). Ring of FPs rotates during the response phase into dimensions of the model that affect the output, executing the correct response based on location of activity in the ring attractor. **G**) Schematic of RandomTarget environment [41]. The TT model was trained to control the effector endpoint (yellow circle) to acquire target (red circle) after a go-cue was provided. External forces (cyan) were applied to perturb the hand, random in timing, direction, and magnitude. We show the resulting kinematics of the hand when applying left/right bumps of variable magnitude to the hand during a reach away from the body. **H**) Example of single trial inputs/outputs for canonical TT-GRU performing RandomTarget. As RandomTarget is coupled, actions (muscle commands) and sensory signals (muscle kinematics, visual inputs) are transmitted between the TT-GRU and the arm. **I**) Latent activity in a behaviorally-relevant plane of canonical TT-GRU when responding to perturbation in **G.** X-axis: Dimension of TT latent activity with strongest linear relationship to x-position of hand. Y-axis: magnitude of projection of TT latent activity onto Pectoralis motor-potent dimension. Bump magnitude (left:blue/right:green-yellow) shown in inset color-map.

Intuitively, RNNs trained to perform 3BFF use stable fixed points to remember the current memory state, and unstable fixed points to guide input-driven transitions between memory states. With low-dimensional activity and well-characterized dynamics, the 3BFF dataset is intended to provide data-driven model developers with clear and relatively unambiguous feedback about model performance. As such, we consider 3BFF to be the introductory task, and recommend it as the first task on which data-driven model performance is evaluated.

**MultiTask**: MultiTask is a previously published task [40,42,43] consisting of 15 distinct cognitive sub-tasks that has been used to investigate shared representations and learning in task-trained networks (Fig 3D–3F). Networks trained on MultiTask have 1) moderately complex dynamics, 2) simple, but relatively high-dimensional inputs, and 3) shared dynamical structures that provide a foothold for interpreting how the computations are performed.

Inputs to MultiTask include a one-of-15 (one-hot) input set that indicates which task is engaged on each trial, 4 noisy sensory input channels, and one fixation input (Fig 3E). For each task, sensory inputs must be transformed into 3-dimensional outputs that follow a task-specific rule. Each task has piecewise inputs that change abruptly across phases– e.g., input presentation, delay period, etc. We show simplified versions of 2 example tasks and their phases in Fig 3D (more details in S1 Text and [40,42]).

Models trained to perform MultiTask learn “dynamical motifs”: shared structures that are reused across multiple tasks [40]. Because MultiTask networks receive piecewise constant external inputs, fixed point (FP) analysis can be used to reveal how the dynamics change across task phases. Previous research studying MultiTask has provided a clear demonstration of how input-dependent changes in FP structure can shed light on how computation is organized in a dynamical system.

To demonstrate how the dynamics of the MultiTask network can illuminate the underlying computation, we analyzed the FP structure of a Multitask TT model performing the MemoryPro task (Fig 3F). As shown previously [42], trained models exhibit a ring attractor of stable FPs that are used to memorize a continuous circular input. When the model was prompted to produce an output corresponding to the input it had seen previously [40], the FP ring rotated into a set of output dimensions that generated the correct response. With its complex and shared dynamical features and its piecewise constant inputs, MultiTask represents the current limits of our ability to interpret task-trained dynamics models, and therefore a challenging but tractable system for data-driven model validation.

**RandomTarget**: The RandomTarget task is based on planar arm reaching, a common experimental paradigm in motor neuroscience [44,45]. Here, a model must learn to control a 2 degree-of-freedom arm–-actuated by 6 Hill-type muscles and simulated with the MotorNet musculoskeletal modeling package [41]–-moving the arm’s endpoint to target locations sampled randomly from within its range of motion, while also correcting for intermittent bump perturbations applied to the hand (Fig 3G).

Models trained to perform this rich motor task have 1) intrinsic dynamics whose effects are difficult to disambiguate from those of their 2) high-dimensional, time-varying inputs, and 3) underlying computations that are not yet well-understood. The model receives both sensory (muscle lengths and velocities, hand endpoint position) and contextual (target position, go cue) inputs, and generates efferent commands that influence the force generated by each muscle. In contrast with previous tasks, this synthetic system is coupled to the environment, meaning that the inputs that it receives are affected by the motor commands it generates (and vice versa, see Fig 3H). As the inputs are constantly changing, this can make direct interpretation of the dynamics very difficult.

To find a signature of dynamical computations underlying movement corrections, we trained a model on RandomTarget and tested how it learned to correct for left/right perturbations in the middle of a reach away from the body (Fig 3G, colored trajectories). To visualize how sensory information is transformed into corrective muscle commands, we projected the TT model’s activity onto the plane defined by the pectoralis muscle activation (responsible for shoulder flexion) and the endpoint *x*-position. We found a stereotyped rotational pattern in this plane that varied smoothly with the perturbation magnitude and direction (Fig 3I, color gradient). TT model dynamics have apparently learned to perform goal-directed sensory-motor transformations that correct for perturbations to the desired hand trajectory; this structure resembles a feedback control system [46].

Unlike the other canonical datasets, the main challenge in RandomTarget is not modeling its dynamics, which are relatively simple, but rather distinguishing the model’s internal dynamics from the coupled dynamics of the TT model and the arm. As biological circuits also receive complex, time-varying inputs that likely reflect coupled dynamics, RandomTarget is also arguably most similar to datasets that might be collected experimentally.

**PhaseCodedMemory**: Cyclic, oscillatory dynamics are a common feature of neural recordings, and we wanted to include a task that lets DD models be tested on a dataset with this dynamical structure. Based on recent work investigating the relationship between oscillatory activity and memory [47], the PhaseCodedMemory (PCM) task simulates a computational mechanism for short-term memory called “phase-coding” that may be present in the hippocampus. Phase-coding models remember the content of input signals by encoding them in the relative phase of an internal oscillation to a reference input oscillation (in this case, an oscillatory signal similar to LFP), as opposed to by differences in mean firing rate across stimulus conditions (i.e., a “rate-coding” strategy). In addition to producing strongly cyclic activity, PCM represents computation in a brain area, the hippocampus (HC), from which recordings differ qualitatively from typical intracortical MEA recordings in both their yield and typical firing rates. The PCM task exhibits simple, interpretable dynamics, yet its time-dependent inputs introduce additional challenges absent from 3BFF. We discuss the differences between the PhaseCodedMemory dataset and the other canonical datasets in further detail in S1 Text.

**ChaoticDelayedMatching**: While the canonical datasets above were designed to emphasize the *regular* dynamics typical of task-trained networks (Section 1.2.2), chaotic dynamics constitute a large and well-studied class of systems that are potentially relevant to neural computation, particularly in the contexts of learning and sensory processing [48,49]. To extend the relevance of CtDToolkit to this regime and to better connect with the broader dynamical-systems reconstruction literature, we include ChaoticDelayedMatching, a delayed non-match-to-sample task based on work by Miconi [50]. Here, a recurrent network of tanh units initialized in a near-chaotic regime (via gain-scaled random recurrent weights) is trained to compare two sequentially-presented binary cues (A or B), separated by delay periods, and to report during a response window whether the cues match (A-A, B-B) or differ (A-B, B-A). This defines a simple and well-specified input-output mapping while requiring the network to maintain and transform information over time. The resulting dynamics exhibit key signatures of chaos in the weakly-driven regime, including sensitivity to initial conditions assessed via the perturbation-driven divergence of nearby trajectories (S5D and S5E Fig), yet still produce the correct task-relevant output during the response period–-reflecting stable computation embedded within high-dimensional, near-chaotic dynamics. We provide additional details on the task and task-trained model in S1 Text.

### 2.4 Metrics and visualizations

Recent research has shown that reconstruction can be an unreliable indicator of model quality [32,33]. CtDToolkit includes new metrics that provide a more holistic account of model performance on key criteria of reconstruction, simplicity, and input accuracy (Section 1.2.2). Some of these metrics (Rate *R*^2^, State *R*^2^, Input *R*^2^) require access to ground-truth, while others (co-smoothing bits-per-spike and cycle-consistency) can be applied even when the ground-truth is unknown. We note that all synthetic datasets in this work were generated with a linear–exponential observation model **g**, in which latent activity is linearly projected to log-firing-rates before exponentiation; unless otherwise noted, the metrics described below assume this linear mapping between latent states and log-rates.

Also included in CtDToolkit are several previously released methods: Dynamical Similarity Analysis (DSA [30]), which quantifies aspects of both reconstruction and simplicity; and two common metrics from the broader field of dynamical systems identification–-a distribution-matching metric based on the Wasserstein distance, and an estimate of the full Lyapunov spectrum that characterizes system stability and sensitivity to initial conditions. We show in Section 3 and S1 Text how these metrics can be used to identify common failure modes of DD models and guide model and hyperparameter selection. We used validation datasets for all metrics and visualizations to prevent overfitting from affecting our interpretations of DD model quality.

#### 2.4.1 Metrics comparing DD models to ground-truth.

**Rate** $R^{2}$: Rate *R*^2^ measures a DD model’s ability to capture features of the true system (i.e., firing rates). Rate *R*^2^ is obtained by finding the coefficient of determination between the true and predicted firing rates of each neuron in the dataset, yielding *N R*^2^ values, which we summarize with the variance-weighted mean across all neurons. By weighting by each neuron’s firing rate variance, Rate *R*^2^ is not artificially deflated by poorly predicted neurons that are only weakly modulated by the task. A Rate *R*^2^ value of 1 indicates perfect reconstruction of the underlying rates for all neurons. As Rate *R*^2^ requires access to ground-truth neural firing rates, it is not typically applicable for DD models trained on biological datasets.


$$\begin{array}{c} R_{\text{Rate}}^{2}=\frac{\sum_{i=1}^{N}\text{Var}(n_{i})R^{2}(n_{i},{\hat{n}}_{i})}{\sum_{i=1}^{N}\text{Var}(n_{i})} \end{array}$$ (1)

**State** $R^{2}$: The possibility of invented features means that a DD model’s reconstruction is an unreliable indicator of accurately inferred dynamics (see Fig 1G). The first CtDToolkit metric for quantifying model simplicity is called State *R*^2^ [32]. State *R*^2^ assesses the degree to which the inferred latent activity contains features that cannot be linearly explained by the ground-truth latent activity. State *R*^2^ is computed by fitting an affine transformation from the true hidden unit activity of the TT model ($z\in {\mathbb{R}}^{D}$) to the activity inferred by the DD model ($\hat{z}\in {\mathbb{R}}^{\hat{D}}$), and then computing the variance-weighted coefficient of determination. As State *R*^2^ requires access to ground-truth latent activity, it is not applicable for DD models trained on biological datasets.


$$\begin{array}{c} R_{\text{State}}^{2}=\frac{\sum_{i=1}^{\hat{D}}\text{Var}({\hat{Z}}_{i})R^{2}({\hat{Z}}_{i},AZ_{i}+b)}{\sum_{i=1}^{\hat{D}}\text{Var}({\hat{Z}}_{i})} \end{array}$$ (2)

**Input** $R^{2}$ As shown in Fig 1H, accurately inferred inputs are critical to ensure that a DD model’s inferred dynamics are trustworthy. We provide a metric called Input *R*^2^ that assesses the accuracy of these inferred inputs. Similar to State *R*^2^, Input *R*^2^ is calculated by fitting an affine transformation from inferred inputs ($\hat{U}\in {\mathbb{R}}^{d_{\hat{U}}}$) to true inputs ($U\in {\mathbb{R}}^{d_{U}}$), then computing the variance-weighted coefficient of determination across input dimensions. As Input *R*^2^ requires access to ground-truth external inputs, it is not applicable for DD models trained on biological datasets.


$$\begin{array}{c} R_{\text{Input}}^{2}=\frac{\sum_{i=1}^{d_{U}}\text{Var}(U_{i})R^{2}(U_{i},C{\hat{U}}_{i}+d)}{\sum_{i=1}^{d_{U}}\text{Var}(U_{i})} \end{array}$$ (3)

#### 2.4.2 Metrics assessing DD models without ground-truth.

Because dynamics and external inputs are often unobservable for biological systems, it is important that we develop metrics that can be applied even when we are unable to compare against ground-truth. In this section, we describe two metrics, co-BPS and cycle-consistency, that quantify DD model performance on the criteria of reconstruction and simplicity.

**Co-Smoothing Bits-per-Spike**: Co-smoothing bits-per-spike (co-BPS) is a previously released method to assess DD model reconstruction [37]. co-BPS quantifies how well spiking activity in a set of held-out neurons can be predicted by models that only have access to a set of held-in neurons, under an assumed Poisson observation model; it is positive when predicted held-out activity is more informative than each neuron’s mean firing rate, and does not require ground-truth firing rates. Co-smoothing is specific to autoencoder-based DD models that condition on observed spikes at inference time, where the held-in/held-out split prevents trivial identity mappings. For DD models that do not require spikes as input, a likelihood computed across all neurons on held-out trials uses more of the data and avoids sensitivity to the neuron split, and should be preferred. co-BPS is calculated as:


$$\begin{array}{c} \text{co-BPS}=\frac{1}{y_{\text{sp}}\log2}\left(ℒ(\hat{𝐧};{𝐲}^{ho})-ℒ(\bar{𝐧};{𝐲}^{ho})\right) \end{array}$$

where **y**^ho^ contains the observed spike counts of the held-out neurons, *y*_sp_ is their total number of spikes, ℒ is the Poisson log-likelihood summed over all held-out neurons and time points, $\hat{𝐧}$ contains the model-predicted firing rates for the held-out neurons, and $\bar{𝐧}$ contains each held-out neuron’s mean firing rate, broadcast across time. Thus, positive co-BPS indicates that the model predicts held-out activity better than the per-neuron mean-rate baseline [37].

**Cycle-Consistency**: Cycle-consistency estimates DD model simplicity *without* requiring ground-truth latent activity, by interrogating the relationship between a model’s inferred latent activity and its inferred firing rates.

The key idea is that a model can only invent features without paying a reconstruction penalty if its inferred observation model $\hat{𝐠}$ is *functionally non-injective* on the data–-that is, if some changes in latent activity produce no (or negligible) change in predicted rates. The canonical example is a linear observation model with a null space, in which latent activity can vary within the null space without affecting predicted neural activity (Fig 1G). The relevant property is functional injectivity of the *learned* mapping at the end of training, not whether the architecture imposes it as a hard constraint: constraining the observation model to be injective [14] is one way to encourage it, but unconstrained networks may also learn functionally injective mappings.

Cycle-consistency quantifies the functional injectivity of $\hat{𝐠}$ on held-out data and uses it as an indirect measure of feature invention: models with high cycle-consistency have few inferred latent features that are not reflected in their predicted neural activity. We emphasize that functional injectivity is necessary but not sufficient to rule out invented features–-a model may introduce latent structure that changes predicted rates only negligibly, escaping detection by spike-level metrics such as co-BPS–-so cycle-consistency is best treated as one diagnostic among several, complementary to ground-truth measures (State *R*^2^, Rate *R*^2^) where available.

Mathematically, cycle-consistency tests whether a linear model can re-generate the inferred latents $\hat{𝐳}$ from the inferred neural activity $\hat{𝐧}$. We first apply principal components analysis (PCA) to the inferred latents and inferred log-rates to sort dimensions by explained variance, then fit a linear observation model from the inferred latents to the inferred log-rates, apply singular-value thresholding to remove effectively-null dimensions (configurable; by default, those associated with <1% of the total variance) [51], and use its pseudo-inverse to re-generate the latents $\tilde{𝐳}$. We then report the variance-weighted *R*^2^ between inferred latents $\hat{𝐳}$ and re-generated latents $\tilde{𝐳}$:


$$\begin{array}{c} R_{\text{Cycle}}^{2}=1-\frac{\sum_{i}w_{i}({\hat{z}}_{i}-{\tilde{z}}_{i}{)}^{2}}{\sum_{i}w_{i}({\hat{z}}_{i}-\bar{z}{)}^{2}} \end{array}$$

Here $w_{i}$ are weights proportional to the variance of each latent dimension; because the metric uses only inferred variables, it can be computed without access to the ground-truth system.

This linear version assumes the canonical linear–exponential link from latent activity to rates; under other link functions or nonlinear manifolds it can mislead, so CtDToolkit also includes a previously published nonlinear version (nl_cycle_con) for arbitrary observation models [14] (S9 Fig and S1 Text).

**Distribution Matching (Wasserstein Distance):**

To connect CtDToolkit with the broader dynamical-systems identification literature, we include a distributional metric based on the empirical Wasserstein distance. Rather than comparing latent trajectories directly, it compares the distribution of *observed* validation activity to the distribution of model-predicted activity, after projecting both into a common, observation-derived low-dimensional space (optionally with a delay embedding to capture local temporal structure). Following Patel and Ott [52], we summarize the discrepancy as an average of per-dimension one-dimensional Wasserstein-1 distances, each normalized by the range of the reference data [15]; lower values indicate closer agreement. Full implementation details–-temporal binning and smoothing, PCA projection, delay embedding, and normalization–-are given in S1 Text.

Because this metric operates in the rate/observation space rather than the latent space, it is likely insensitive to invented features that fall in the null space of the inferred observation model $\hat{𝐠}$: such features leave predicted activity unchanged, and so do not register in a distributional comparison of observed and predicted activity.

#### 2.4.3 Additional metrics and visualization.

**Dynamical Similarity Analysis (DSA)**: DSA [30] is a nonlinear similarity metric that compares the spatiotemporal structure of two dynamical systems. Applied to CtDToolkit, DSA measures whether DD models reconstruct the core dynamic features of their TT model. More specifically, DSA captures whether these features (e.g., fixed points) are aligned in a one-to-one fashion and the DD model has neither invented superfluous features nor deleted relevant ones. DSA has three components (further details are in S1 Text): first, the method lifts the data into a higher dimensional space using kernels or delay embeddings [53,54]. Second, a linear system is fit to each embedding, resulting in dynamics matrices $A_{x},A_{y}$. Finally, these systems are compared using an extension of Procrustes Analysis [55]. In this paper, we used the angular form of the distance, which scales from 0 (most similar) to π/2 (most dissimilar). In practice, however the full range is not always used, and so relative distances are typically emphasized over absolute. As applied here, DSA compares the inferred dynamics of a DD model against the dynamics of the ground-truth TT model, and therefore requires access to the true latent activity; it is consequently applicable only to synthetic datasets where ground truth is available, and not to biological recordings.


$$\begin{array}{c} \text{DSA}(A_{x},A_{y})=\min_{C\in O(n)}||A_{x}-CA_{y}C^{-1}|{|}_{F} \end{array}$$ (4)

**Fixed-Point Visualization**: In addition to the quantitative metrics, CtDToolkit provides tools for qualitatively assessing the true and inferred dynamics through their fixed point structures. We distinguish *true fixed points*, where the flow is exactly zero (*f*(*z*,*u*) = *z*), from *functional fixed points*: regions of state space where the dynamics are sufficiently slow (or contracting) to appear stable over behaviorally relevant timescales and to permit linear approximation. In practice, the fixed-point finder below locates functional fixed points by minimizing (rather than exactly zeroing) the speed of the dynamics. The arrangement and stability of these fixed points provide key insights into the local behavior of the system. To find fixed points, we find locations in the model’s state space ($Z\in {\mathbb{R}}^{d_{z}}$) that minimize the magnitude of the kinetic energy of the system ($\Delta h^{2}\sim q$). CtDToolkit includes an extension of a previously-released fixed point finding toolkit [39] for use with both TT and DD models. Since input changes can alter fixed point structure [40,56], our modified fixed point finder accepts user-defined static inputs, allowing researchers to visualize how fixed point structures change as inputs change.

**Lyapunov Spectrum** The Lyapunov spectrum characterizes the long-term behavior of a dynamical system by quantifying how quickly nearby trajectories diverge or converge along different directions; it is a standard tool for comparing the stability and sensitivity-to-initial-conditions of an inferred model against a ground-truth system. Following [57], we estimate the full spectrum $\left\{\lambda_{1}\geq \cdots \geq \lambda_{D}\right\}$ of the discrete-time system ${𝐳}_{t+1}=f({𝐳}_{t},{𝐮}_{t})$ using the QR-decomposition method applied to Jacobians sampled along trajectories. The maximal exponent $\lambda_{\max}$ is the component most readily estimated from data and most directly compared across TT and DD models ($\lambda_{\max}>0$ indicates chaos; $\lambda_{\max}<0$, asymptotic stability), but the full spectrum is needed to distinguish, for example, a chaotic *attracting* set from a divergent one–-via summaries such as the sum of exponents $\sum_{i}\lambda_{i}$ (the mean rate of phase-space volume contraction) and the Kaplan–Yorke dimension. CtDToolkit provides compute_lyapunov_spectrum (and Analysis_TT.compute_lyapunov_spectrum) routines for this purpose. We validated the estimator on the canonical Lorenz and Rössler systems, recovering spectra that closely match their literature references (S10 Fig). Full algorithmic details and validation values are provided in S1 Text.

## 3 Results

### 3.1 Canonical datasets provide a library of biologically-motivated dynamical systems

**Task-training and dataset simulation pipeline:**

We first used a CtDToolkit task environment to generate training data–-external inputs, target outputs, and the additional information needed for training (S1 Text)–-and trained a task-trained (TT) dynamics model to produce outputs that minimize the task’s objective function (Fig 4A, left). By the end of training, each TT model had learned latent dynamics that performed the computation defined by its task environment.

**Fig 4:**
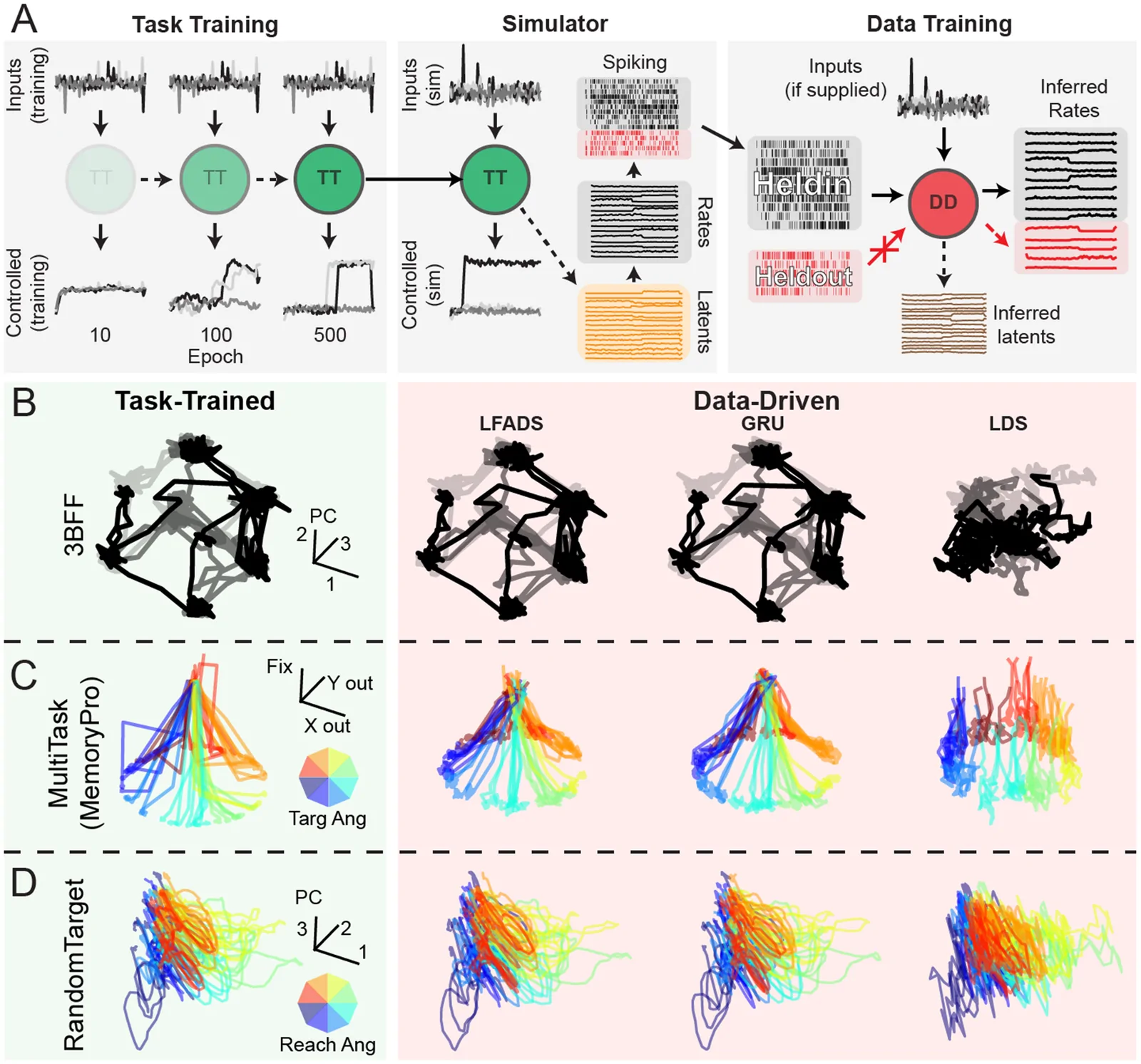
Task-trained and data-driven modeling pipeline, and example inferred latent activity for a subset of canonical datasets: A) Schematic of task-training, simulation, and data-training pipelines: (left) Task-training: model inputs converted to control signal by TT model dynamics. Over training epochs, TT models learn to generate control output that accomplishes the task. (middle) Fully-trained model provided as input to the simulator. Simulator was used to sample latent activity from the TT model, convert to rates, and simulate spiking using a Poisson noise process. (right) Simulated spiking split into held-in and held-out sets of neurons, and into training and validation trials. Held-in spiking is fed as input to the DD model, which is trained to infer rates for both held-in and held-out neurons. Hidden activity of the DD model is referred to as inferred latent activity. All metrics are computed on the validation trial set to prevent our results from being due to trivial overfitting. **B-D)** Example ground-truth latent activity (left column, green, without noise in input channels for improved visualization) and latent activity inferred by DD models (from left to right, LFADS, GRU, LDS) for three canonical datasets. DD model inferred latent activity was aligned with TT latent activity using an optimal affine transformation. **B)** Latent activity (top 3 PCs) for three representative trials (indicated by opacity) for 3BFF canonical dataset. **C)** Latent activity for representative trials of MemoryPro subtask for MultiTask canonical dataset. Color of each trace indicates the angle of the correct output response (inset color wheel). Latent activity was projected onto a 3D subspace defined by response 1 (x-axis), response 2 (y-axis), and fixation (z-axis) output dimensions. For DD models, we transformed the inferred latents to the space of the ground-truth latents via an optimal affine transformation, then projected through the ground-truth output mapping. **D)** Example inferred latent activity (top 3 PCs) for representative trials for RandomTarget canonical dataset. Color indicates the reach angle for each trial.

We then simulated neural datasets from the hidden-unit activity of the TT models (Fig 4A, middle; S1 Text). We fed the task-training trials to each TT model, recorded its hidden activity, and–-for most datasets–-sampled *N* hidden dimensions without replacement, scaled them, and used them as the rate parameter of a Poisson process. (The PhaseCodedMemory dataset is an exception: to emulate a larger, low-firing-rate hippocampal population, its neuron count exceeds the TT latent dimensionality, so some latent dimensions are sampled more than once with independent observation noise; S1 Text) For every dataset, this yielded spiking activity from *N* simulated neurons, which we then split into held-in and held-out neuron sets. These held-out neurons should not be confused with held-out validation trials; all performance metrics were computed on validation trials to avoid spurious results from overfitting.

We designed the 3BFF, MultiTask, RandomTarget, and ChaoticDelayedMatching datasets to have properties that resembled those of common intracortical multi-electrode array recordings (e.g., from Utah arrays), with *N* < 100 and moderately high firing rates. In contrast, the PhaseCodedMemory dataset was designed to emulate the properties of datasets recorded from CA1 neurons in the hippocampus, and was therefore composed of 600 neurons (500 held-in, 100 held-out) with relatively sparse activity (~2 Hz) (S4A–S4E Fig). We trained DD models to reconstruct held-in and held-out neural activity using only held-in neural activity (Fig 4A, right).

#### Key observations from canonical datasets:

To provide some intuition for how common DD models perform on the canonical datasets, we visualized the ground-truth and inferred latent activity for the tasks included in CtDToolkit and compiled the full performance-metric sweeps across model sizes for each: 3-Bit Flip-Flop (3BFF; S11 Fig), MultiTask (S12 Fig), RandomTarget (S13 Fig), PhaseCodedMemory (S14 Fig), and ChaoticDelayedMatching (S15 Fig). We provide descriptions of the three baseline DD models in S1 Text.

*3-Bit Flip-Flop (3BFF)*: For 3BFF (Fig 4B), we found that LFADS and GRU models inferred latent activity that closely resembled the top three principal components (PCs) of the ground-truth latent dynamics. The LDS model, however, lacked the computational capacity to capture even the relatively simple dynamics of 3BFF. In other words, LDS suffers from the failure mode of *underfitting* described in Section 1.2.2. We note that the LDS failure is more precisely described as *structural underfitting*: the class of representable (linear) dynamics simply does not contain the target system, regardless of parameter values. This contrasts with the capacity-limited underfitting of more expressive models (e.g., a NODE with too few latent dimensions), which can in principle represent the true dynamics but fail to do so with insufficient capacity.

*MultiTask*: In the MultiTask dataset (Fig 4C), we visualized the behavior of our DD models by projecting the inferred latent activity from MemoryPro trials onto three output dimensions (Fixation, X output, Y output) for each model. We found that both GRU and LFADS models qualitatively captured the structure of the ground-truth latent activity in this 3D subspace. However, the LDS model had significant errors in its projection onto output dimensions during the memory period. This suggests that LDS was unable to gate its response appropriately, resulting in premature output generation. Similar to 3BFF, these results indicate that LDS lacks computational capacity, and as a result also underfits the dynamics of the MultiTask dataset.

*RandomTarget*: Given its complex time-varying inputs, performance on RandomTarget (Fig 4D) is the most difficult to interpret. Examining the top three PCs of latent activity (color-coded by reach direction), all three models–-including the limited-capacity LDS–-appeared to capture the high-level structure of the ground-truth latents. This apparent success is deceptive: because every model was supplied the rich, time-varying ground-truth inputs (muscle lengths and velocities, vision, and goal/timing information) during both training and inference, much of that structure can be inherited directly from the inputs rather than generated by a model’s own intrinsic dynamics. That even LDS–-which underfit the far simpler 3BFF and MultiTask–-looks good here is precisely the warning: on RandomTarget, matching the ground-truth latents says little about whether a model has captured the underlying dynamics. The real challenge is to disentangle intrinsic dynamics from input-driven activity. This tight coupling between the TT network and another dynamical system makes RandomTarget a more realistic and conceptually challenging addition to CtDToolkit than alternative datasets [46].

*PhaseCodedMemory*: We show the performance of one model class (LFADS) on the PhaseCodedMemory dataset in S16 Fig. We found that the LFADS model inferred latent activity that pointed towards a “rate-coding” strategy of memory, in contrast with the “phase-coding” strategy of the dynamics of the ground-truth TT model. This contrast is also visible geometrically: in the TT model the latent-space and rate-space principal components share the same structure, whereas in the DD models they diverge–-the inferred latents separate the two stimulus conditions even though the reconstructed rate-space structure does not. Notably, this divergence emerges even as reconstruction *improves* with model capacity: across the latent-size sweep, Rate *R*^2^ increases (from 0.68 to 0.77) while State *R*^2^ degrades (from 0.97 to 0.92), so a practitioner selecting models on reconstruction alone would be most confident in precisely the regime where the inferred latent geometry is most misleading (S1 Text). This result illustrates how interpreting a DD model’s inferred latent dynamics at face value–-without cross-checking against a ground truth or the simplicity metrics advocated here–-could lead researchers to incorrect conclusions about how computation is performed in the brain, even when the observed activity is reconstructed well.

*ChaoticDelayedMatching*: ChaoticDelayedMatching extends the canonical set to the near-chaotic regime, in which task-relevant computation is embedded in high-dimensional, sensitive-to-initial-conditions dynamics rather than the regular, attractor-driven dynamics of the other canonical datasets. ChaoticDelayedMatching thus provides a setting in which the reconstruction and dynamical-stability metrics in CtDToolkit (S1 Text) can be exercised on a system whose autonomous dynamics are qualitatively closer to those of the cortical networks the toolkit ultimately aims to model.

### 3.2 Reconstruction and simplicity metrics guide model selection by identifying common failure modes

Next, we sought to demonstrate that CtDToolkit metrics can provide actionable feedback for improving DD models. We trained DD Neural ODE (NODE) models [58] to reconstruct synthetic spiking activity from our canonical 3BFF TT model (S1 Text). Because a NODE’s latent dimensionality controls the expressivity of its dynamics, we trained 5 randomly initialized models at each hidden size $\hat{D}\in [2,3,5,8,16,32,64]$ (35 models total), all supplied with ground-truth external inputs; this lets us study how model capacity drives distinct failure modes.

We emphasize that latent dimensionality is only one of many hyperparameters that affect model performance: regularization strength, input penalties, training duration, architecture, and optimization settings can all influence whether an expressive model underfits, faithfully recovers the dynamics, or invents spurious features. For example, the number of training epochs can itself act as a control on model complexity: prior work has shown it can dramatically affect the fidelity of the inferred dynamical landscape even while generalization performance remains high [33].

**Reconstruction:** We measured each model’s reconstruction accuracy with Rate *R*^2^ and co-BPS (as described in Section 2.4; Fig 5A). Inspecting the inferred firing rate of a single held-out neuron, we found that while DD NODE with 2 dimensions (NODE-2) was able to capture some aspects of the true firing rate, its reconstruction was substantially worse than a DD NODE with 8 hidden units (NODE-8, Fig 5B, dark blue, green, respectively). Models with a very small number of hidden units apparently had insufficient capacity, and therefore suffered from *underfitting*.

**Fig 5:**
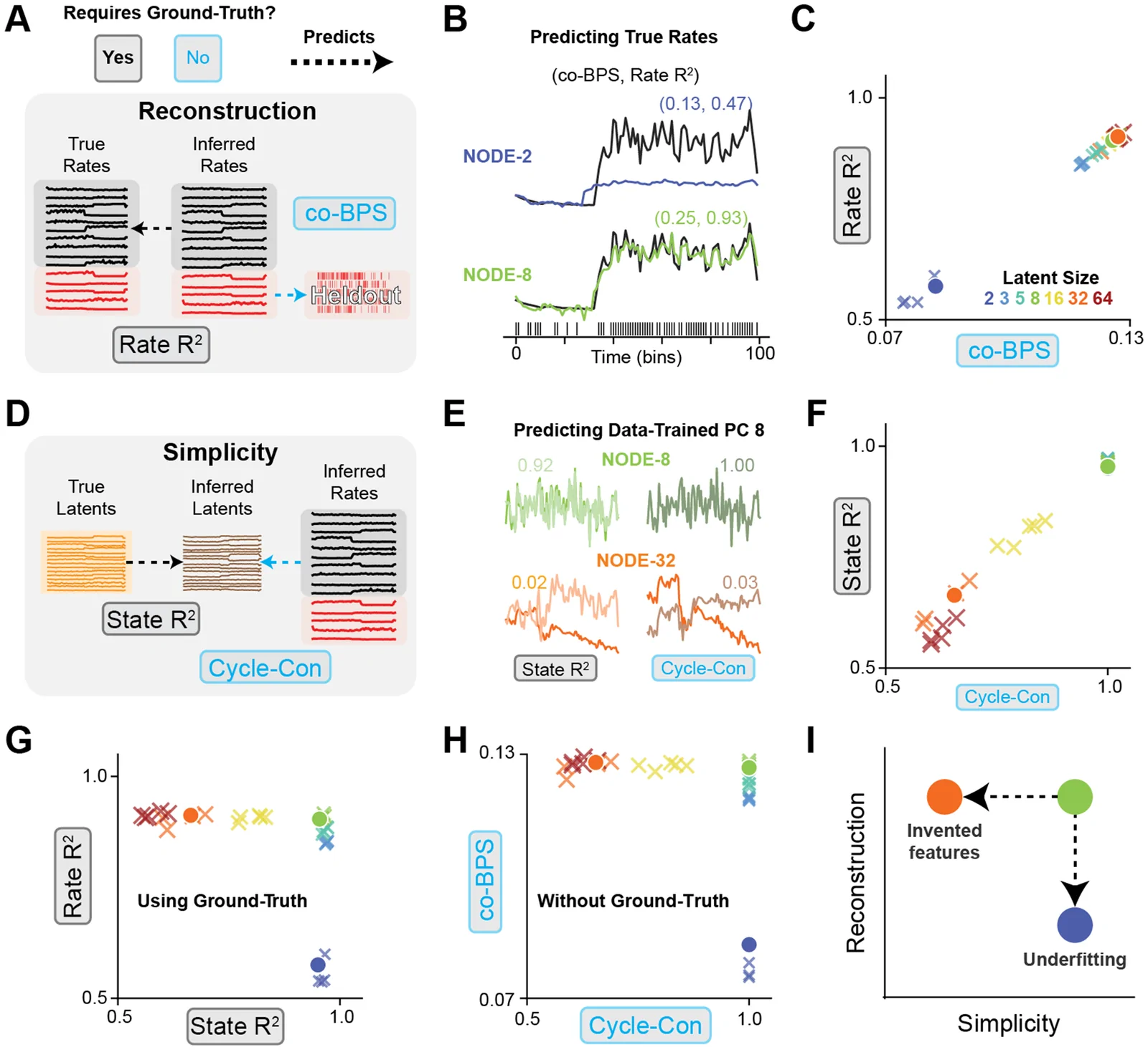
Reconstruction and simplicity metrics for data-driven modeling of 3BFF dataset. **A)** Schematic of reconstruction metrics: Rate *R*^2^ measures prediction accuracy of inferred rates vs. true rates. co-BPS measures accuracy of held-out spiking predictions. **B**) Single-trial predicted firing rate of one held-out neuron from models NODE-2 (2 hidden unit NODE, dark blue) and NODE-8 (8 hidden unit NODE, green). Performance indicated by (Rate *R*^2^, co-BPS) inset. **C)** Scatter plot of Rate *R*^2^ vs. co-BPS for NODE models ranging from 2 to 64 hidden units. Inset legend color indicates hidden size. Circles indicate example models in panel B, Xs indicate other randomly initialized models with the indicated latent dimensionality. **D)** Schematic of simplicity metrics: State *R*^2^ measures prediction accuracy of inferred latents from true latents, cycle-con measures accuracy of inferred latents from inferred rates. **E)** Inferred latent activity PC8 (green = NODE-8, orange = NODE-32), and linear prediction from true latents (left, State *R*^2^) or inferred rates (right, cycle-con). **F)** Scatter plot of State *R*^2^ vs. cycle-con for same models in panel C, indicated by colored markers. **G)** Scatter plot of Rate *R*^2^ vs. State *R*^2^, measuring reconstruction and simplicity with access to ground-truth. **H)** Scatter plot of co-BPS vs. cycle-con, showing metrics of reconstruction and simplicity that can be computed without access to ground truth. **I)** Schematic of underfitting (dark blue, NODE-2) and invented features (orange, NODE-32) failure modes, for comparison with panels G, **H.**

To confirm that our metric for quantifying reconstruction without access to ground-truth firing rates (co-BPS) gave similar results, we plotted the performance of our 35 models on these two metrics and found a strong linear relationship between Rate *R*^2^ and co-BPS (Fig 5C). The co-smoothing metric used to quantify reconstruction in previous benchmarks [37] seems to accurately capture reconstruction, even in the absence of ground-truth.

**Simplicity:** We assessed the simplicity of each model to determine the extent to which it had invented features (Fig 5D). To do this, we visualized the inferred latent activity in a low-variance dimension (8th principal component) for two DD models (Fig 5E, green: NODE-8, orange: NODE-32), along with linear predictions of this dimension from the true latents (State *R*^2^, left column) and inferred rates (Cycle-Con, right column). We found that the 8th PC of NODE-32 was predicted poorly compared to the 8th PC of NODE-8. The presence of features in the inferred latent activity that cannot be predicted from the ground-truth suggests that the more expressive models tended to invent features that didn’t exist in the underlying system.

To confirm that our metric for quantifying simplicity without access to ground-truth latents (cycle-consistency) gave similar results to our ground-truth simplicity metric (State *R*^2^), we plotted the performance of our 35 models on these metrics and, like our reconstruction metrics, found a strong linear relationship (Fig 5F). Additionally, we found that as NODE hidden size increased, there was a consistent decline in the simplicity for both metrics, evidence that more expressive models can suffer from the failure mode of *feature invention*.

**Model selection across multiple criteria:** We plotted the two criteria together, showing metrics that rely on access to ground-truth (Fig 5G) and metrics that do not (Fig 5H). Both sets of metrics qualitatively recapitulate the failure modes described by our performance criteria presented in Fig 1. Models with insufficient expressivity suffered from underfitting, while models that were too expressive invented features (Fig 5I). We found similar results when we evaluated the performance of DD models trained on the NBFF, MultiTask, RandomTarget, PhaseCodedMemory, and ChaoticDelayedMatching canonical datasets (see S11–S15 Figs).

**Complementary metrics:** We also computed two metrics from the broader dynamical-systems identification literature–the Wasserstein distance and the Lyapunov spectrum–-for all DD models (S11C Fig). The Wasserstein distance gave a ground-truth-free distributional comparison between model-predicted and observed activity across latent sizes; in practice it generally tracked reconstruction performance and was insensitive to invented features, consistent with its operating in the rate/observation space. The Lyapunov spectrum, in contrast, characterized the stability of each model’s inferred dynamics directly from its flow field. Estimating the spectrum for both TT and DD models, we found that the DD models whose maximal Lyapunov exponent best matched the ground-truth TT estimate were those with 8–16 latent dimensions: precisely the regime in which reconstruction had saturated and simplicity was beginning to degrade. The Lyapunov spectrum thus offers a complementary axis for model selection: rather than comparing inferred activity patterns (as the reconstruction and simplicity metrics do), it compares the inferred *dynamics* themselves, and here it independently pointed toward the same well-balanced models. Together, these metrics give users additional tools for diagnosing DD model failures.

Nonlinearities in the mapping between two topologically identical systems could lead some metrics to incorrectly diagnose a model with invented features. We caution that geometric differences cannot always be safely ignored: recent work shows that the geometry of inferred activity can itself reflect genuinely distinct task solutions rather than trivial reparameterizations [59]. For this reason, CtDToolkit includes both linear and nonlinear metrics as complementary measures of model similarity, leaving the choice of whether to treat geometric differences as meaningful to the user.

### 3.3 Input $R^{2}$ helps diagnose models that fail by dynamical misattribution

The experiments above assumed known ground-truth inputs, which biological neural datasets rarely provide. A further CtDToolkit metric, Input *R*^2^, quantifies how accurately a model infers these unseen external inputs. Throughout this work we assume the dimensionality of the external inputs is known; this lets us evaluate inferred dynamics without the additional ambiguity of input-dimensionality estimation, which we regard as an important related problem left for future work.

A common architecture for input inference (originally introduced in LFADS [10]) is shown in Fig 6A. Neural activity is provided to two separate modules, the controller and the generator, which produce the inferred inputs and underlying dynamics respectively. Input inference is in general an ill-posed problem because observations alone are insufficient to distinguish a purely input-driven system from a purely autonomous one. Thus, the form of inferred inputs is heavily influenced by model hyperparameters. In the case of LFADS, two critical choices are the prior distribution of the inferred inputs and the magnitude of the penalty for divergence from that prior.

**Fig 6:**
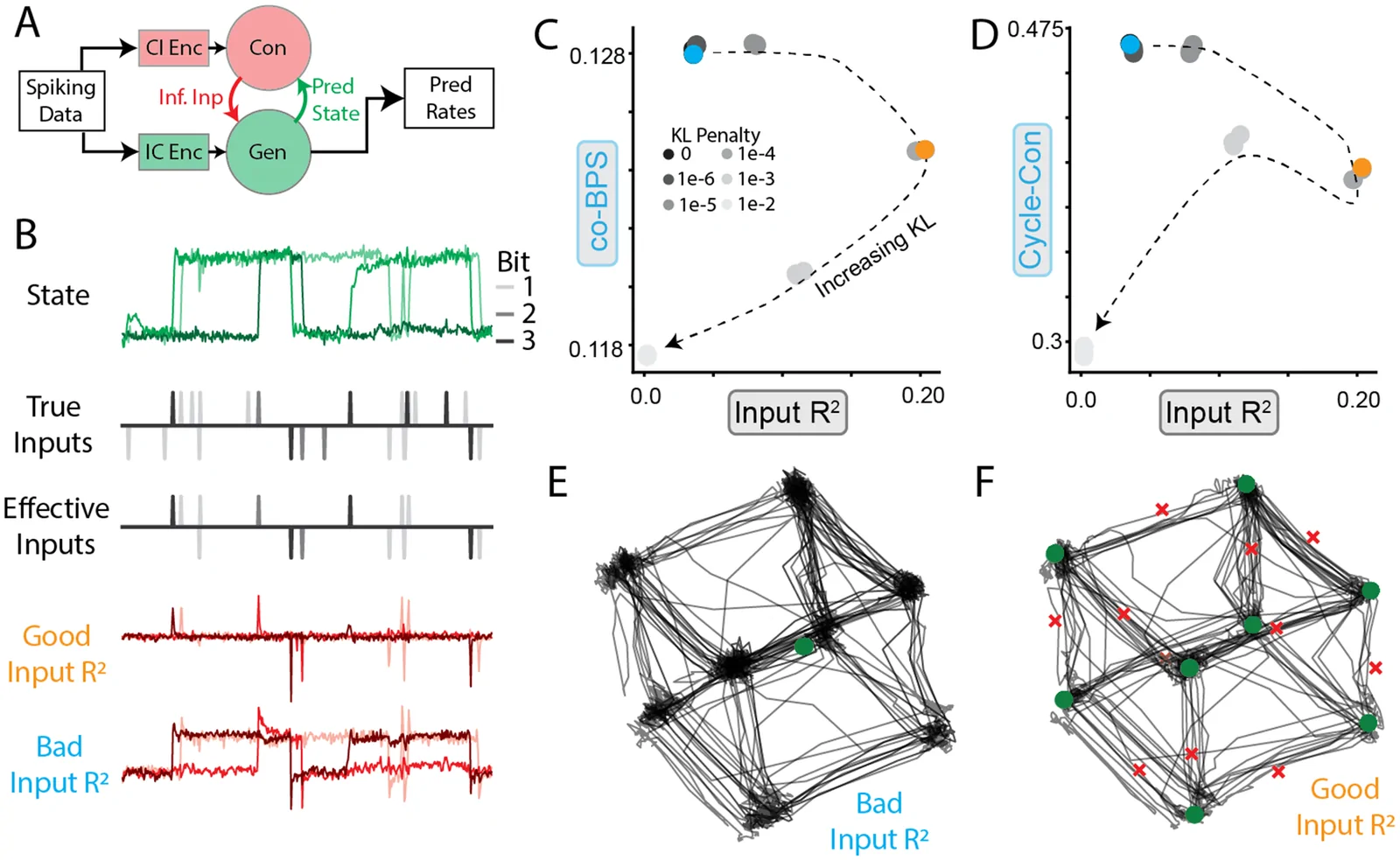
Inferred inputs affect inferred dynamics. **A**) Schematic of common input inference procedure for DD models. IC Enc, CI Enc denote Initial Condition encoder, Controller Input encoder, respectively. Ideally, the generator only models the intrinsic dynamics of the circuit and the controller only predicts inputs when inputs are actually present. **B**. Example activity from a single trial of 3BFF task. Shading indicates bit number. Top row: Output of TT-GRU. Rows 2-3: True and effective inputs (inputs that are expected to affect the state). Rows 4-5: Inferred inputs for two example models with “good” (orange) and “bad” (cyan) input inference. **C**) Scatter plot of co-BPS vs. Input *R*^2^ for models with increasing KL penalty (indicated by shading/ dashed line). Models depicted in B shown in orange/cyan. **D**) Same as C, except cycle-consistency vs. Input *R*^2^. **E**) Inferred FP architecture of model colored in cyan in **(C)**. This model infers no FPs at the vertices of the cube, or any unstable FPs. Note that these are input-determined fixed points, computed under the relevant static inputs to the model (the dynamics, and hence the fixed point structure, are a function of the inputs), rather than fixed points of the autonomous (zero-input) system. **F**) Same as E, except for orange model in **(C)**. Stable (unstable) FPs are depicted as green circles (red ×s). Compare to ground-truth FP structure in Fig 3C.

To validate that using Input *R*^2^ to select hyperparameters leads to accurate inferred inputs and dynamics, we fit multiple LFADS models with different weights on the prior distribution of inferred inputs (Student’s *t*-distribution with *df* = 5, which promotes sparse inputs) to data from the 3BFF canonical dataset. We compared the inferred inputs to both the true inputs and the “effective” inputs (i.e., inputs that would change the sign of the bit, as opposed to redundantly indicating the current sign, Fig 6B, top three rows). Some models inferred inputs that qualitatively matched the effective inputs, while other models’ inferred inputs resembled the state of the network.

Plotting the models’ co-BPS and cycle-consistency against the Input *R*^2^ (Fig 6C and 6D) demonstrated that many models with high co-BPS and cycle-consistency had extremely poor input inference. As the KL penalty increased, Input *R*^2^ increased until the KL penalty became high enough that the model began to suffer from underfitting (Fig 6C). Interestingly, we found that models with both high and low Input *R*^2^ could find inferred activity that was almost identical to the true system (Fig 6E and 6F, black traces). However, fixed-point analysis revealed that only models with high Input *R*^2^ reproduced the expected FP structure (Fig 6E and 6F, ○, × markers), while those with low Input *R*^2^ failed to capture any of the relevant FPs. This suggests that the model with low KL penalty suffered from the failure mode of *dynamical misattribution*, in which the controller takes over the relevant dynamics from the generator (Fig 1H).

Together, these results indicate that reconstruction and simplicity alone may be insufficient when input inference is considered. Reconstruction, simplicity, and input accuracy each provide a piece of the puzzle for selecting data-driven models that can accurately infer dynamics from recorded neural activity. However, we caution that the hyperparameters found to produce good Input *R*^2^ on this dataset are unlikely to work well on datasets with different properties; since Input *R*^2^ relies upon knowledge of the ground-truth, this presents a major unresolved challenge for the field of data-driven dynamical modeling. Multi-area recordings and perturbations will likely be essential for resolving the ambiguities associated with external input inference.

## 4 Discussion, limitations, and future directions

The Computation-through-Dynamics Toolkit (CtDToolkit) represents a significant step towards evaluating goal-directed neural computations through neural dynamics modeling. By offering standardized datasets and interpretable metrics, CtDToolkit provides researchers with tools to validate data-driven (DD) models, even when both dynamics and inputs must be inferred. Its modular and extensible design invites contributions from the community, promoting continued evolution to address emerging challenges.

### 4.1 Related approaches

The Neural Latents Benchmark (NLB) [37] demonstrated the value of standardized datasets and a shared evaluation metric (co-BPS) for comparing latent-variable models of neural population activity, and it catalyzed substantial methodological progress in the field. A key distinction is that NLB evaluates models against held-out neural activity, whereas CtDToolkit’s synthetic datasets provide access to the ground-truth latent dynamics and inputs, enabling us to directly assess whether inferred dynamics match the true system rather than only whether neural activity is well reconstructed. We therefore view CtDToolkit as complementary to NLB: NLB grounds evaluation in real recordings, while CtDToolkit provides the ground-truth control needed to validate that reconstruction- and simplicity-based metrics actually track dynamical accuracy.

A related lesson from NLB is that presenting a single headline metric to optimize against can, over time, lead researchers to focus on incrementally improving the number rather than on understanding the computations the brain performs. This risk is an instance of Goodhart’s law–-once a measure becomes a target, it can cease to be a good measure. We designed CtDToolkit to guard against this tendency by reporting a suite of complementary criteria (reconstruction, simplicity, and input accuracy) rather than a single score, so that no one metric can be optimized in isolation without exposing trade-offs that a single number would obscure.

NLB also taught us that users tended to focus on a small subset of “workhorse” datasets rather than engaging broadly with the full range of available benchmarks. This suggested that including an extensive collection of diverse datasets could potentially dilute efforts across many standards. Here, we prioritize a smaller, curated set of validated datasets to help the field consolidate around common standards of model evaluation, accepting that this approach may limit applicability to neural recordings with substantially different characteristics or computational demands. We hope that researchers will continue to expand the collection of datasets by submitting their tasks and models to CtDToolkit following the procedure described in S1 Text.

Selecting DD models that recover the true dynamics can be approached in complementary ways, and CtDToolkit deliberately commits to one: a *metric-based* approach that supplies interpretable metrics flagging whether a model has fallen into a characteristic failure mode (e.g., underfitting), which can then be used to compare and select among candidates. A complementary strategy instead seeks *consistent* solutions–-training multiple models and keeping those whose inferred features agree, or identifying the minimum-complexity regime in which solutions become reproducible across independently trained models [33]. Both target the same goal, so we expect models favored by one to fare well under the other; we adopt the metric-based approach here, but encourage readers to consider consistency-based alternatives where appropriate.

More broadly, we see CtDToolkit as an opportunity to build bridges between the neural-dynamics modeling community and the broader field of dynamical systems reconstruction (DSR), much of which has developed largely outside of neuroscience [15–17,52]–-with insight flowing in both directions. From DSR, neural-dynamics modeling stands to inherit a mature body of experience validating inferred dynamics against ground truth using measures of *invariant* structure. These include geometric measures, such as state-space (Kullback–Leibler) divergence [16,28] and Wasserstein distance between distributions of visited states [52], as well as temporal measures, such as power-spectrum or autocorrelation agreement [16,17]. Such measures are typically computed in a shared, observation-derived space without requiring ground-truth latents or trial-by-trial alignment. Our Wasserstein distribution-matching metric brings this perspective into CtDToolkit, and incorporating the complementary temporal measures is a natural next step. In the other direction, the neural setting confronts DSR with challenges that its canonical (autonomous, non-neural) benchmarks largely lack–-unknown external inputs, feedback, and explicit goal-directed computation–-and CtDToolkit packages these as concrete, ground-truthed benchmark systems on which DSR methods can be stress-tested and extended. By offering a shared platform and common metrics, CtDToolkit can help the two communities exchange both tools and problems, which we see as a promising avenue for progress.

### 4.2 Limitations

Despite its strengths, CtDToolkit faces limitations rooted in our incomplete understanding of neural dynamics. First, the TT systems used here are rate-based: neural spikes are produced downstream by an observation model rather than by the task-performing network itself. A natural next step would be to incorporate spiking neural networks as TT systems, allowing the benchmark ground truth to include membrane, synaptic, and spike-timing dynamics. Such datasets would test when rate-based descriptions are sufficient and broaden the toolkit’s applicability to temporally coded computation. Other extensions could incorporate diffuse neuromodulatory influences. Second, our datasets and most of our metrics assume a linear observation model mapping latent dynamics to neural activity. While CtDToolkit includes the non-linear DSA and nl_cycle_con methods [14,30], further work is needed to assess how deviations from linearity affect model performance and to develop additional metrics that are robust to these deviations [20,21].

A further limitation is that all of our metrics are computed on inferred latent trajectories and firing rates rather than on the inferred flow field directly. Trajectory- and rate-based metrics can be confounded by observation noise and input-driven variability, whereas an error metric defined between the inferred and ground-truth flow fields would avoid these confounds. Such a comparison is not straightforward for the GRU-based TT architectures used here, but it is feasible for architectures whose vector field can be evaluated directly (e.g., low-rank RNNs). We therefore view direct comparison between inferred and ground-truth flow fields, enabled by appropriate TT model architectures, as a valuable direction for future work.

Input inference remains a major challenge. Although metrics like co-BPS and cycle-consistency estimate reconstruction and simplicity without ground-truth, CtDToolkit lacks an analogous metric for input accuracy. Simulated perturbations and new metrics for quantifying input inference accuracy could help address this issue. We consider this to be a major focus of future work in this area.

Looking forward, CtDToolkit’s extensibility offers pathways for addressing these limitations. Contributions such as richer datasets, novel metrics, and improved nonlinear methods can enhance the utility of the toolkit. With continued community collaboration, CtDToolkit can drive innovations that deepen our understanding of neural computation and help bridge the gap between models and biological systems. In summary, CtDToolkit not only provides a foundation for evaluating neural dynamics models but also a launchpad for broader investigations into neural computation. As the field advances, CtDToolkit has the potential to play a key role in shaping tools and frameworks that uncover fundamental principles of brain function.

## Supporting information

S1 Text Supplementary methods and implementation details. (PDF)

S1 Table Parameters for the task-training and simulation datasets for 3BFF. (CSV)

S2 Table NoisyGRU model parameters (3BFF). (CSV)

S3 Table MultiTask phase length by trial type: [min, max] in bins. (CSV)

S4 Table Task-training dataset parameters (MultiTask). (CSV)

S5 Table NoisyGRU model parameters (MultiTask). (CSV)

S6 Table Task-training dataset parameters (RandomTarget): all timings are in bins (10 ms). (CSV)

S7 Table NoisyGRU model parameters (RandomTarget). (CSV)

S8 Table Parameters for the task-training and simulation datasets for PhaseCodedMemory. (CSV)

S9 Table NoisyGRU model parameters (PhaseCodedMemory). (CSV)

S10 Table Canonical dataset simulation parameters. (CSV)

S11 Table Hyperparameters for data-driven models (NODE-D) from Fig 5. (CSV)

S12 Table Hyperparameters for data-driven LFADS models in Fig 6. (CSV)

S13 Table Hyperparameters for the DSA fits and fixed-point finding in S8 Fig. (CSV)

S14 Table Hyperparameters for the NODE-based SAE latent-size sweeps in the compiled-metrics figures (NBFF, MultiTask, RandomTarget, ChaoticDelayedMatching). (CSV)

S15 Table Hyperparameters for the LFADS latent-size sweep on PhaseCodedMemory in the compiled-metrics figure. (CSV)

S1 Fig Schematic of task-training pipeline. Here we show the general procedure for running task training with the structure of the objects that appear in the code, and their interactions, shown graphically. First, the data that define the inputs/outputs of a task must be generated. If the dataset has already been generated, it can be found in /content/datasets/tt; otherwise, the datamodule will call the method generate_dataset() from the TaskEnvironment. The task data will then be split into training and validation datasets by the DataModule. In the case of coupled environments that require feedback at each step (e.g., RandomTarget), the TaskEnvironment is also loaded. CtDToolkit uses the TaskWrapper to coordinate training and saving, so each of these objects are passed together to the TaskWrapper which runs the task-training pipeline described in S1 Text. When training is complete, the DataModule, TaskEnvironment, and task-trained model are saved to /content/trained_models.
(PNG)

S2 Fig Example trial for 3BFF. Example input pulses, target states, and task-trained model outputs for one 3BFF trial.
(PNG)

S3 Fig Example trials for MultiTask. We show one example trial for each task in MultiTask, organized in columns by trial category. Each trial has four subplots, with the task name indicated to the left of each row. Each subplot shows a 2D signal over time as a scatter plot, color coded by task phase. The left two plots indicate the inputs to stimulus modalities 1 and 2, respectively. The third plot indicates the model output on its two response channels. The rightmost plot indicates the target output.
(PNG)

S4 FigPhaseCodedMemory task-trained model and simulated data.**A)** Schematic of the PhaseCodedMemory task. The model is trained to generate an output oscillation shifted by 0.2π for input channel A (purple, Stim A) or by 1.2π for input channel B (cyan, Stim B), relative to the dashed reference oscillation. **B)** Two example TT-model trials that differ only in the stimulated input channel. **C)** Top three PCs of post-stimulus TT-model latent activity; the two conditions form overlapping, primarily rotated rings consistent with phase coding. **D)** Example TT-network single-unit activity phase-shifted between stimulus conditions. **E)** Ground-truth firing rates and Poisson-simulated spiking activity for the synthetic PhaseCodedMemory dataset. An example data-driven model fit and validation on this dataset is shown in S16 Fig.(PNG)

S5 Fig ChaoticDelayedMatching task-trained model and chaotic signatures. **A)** Delayed non-match-to-sample task structure with example cue inputs and target output. **B)** One task-trained latent trajectory for each of the four trial types, colored by trial phase. **C)** Task-trained model output for an example trial and an ensemble of small initial-condition perturbations; the outputs converge to the correct response. **D)** The corresponding initial-condition perturbations in the top two and top three PCs of latent activity. **E)** Trial-averaged growth of the normalized perturbation norm in the full latent space (purple) and output space (blue). Latent perturbations expand while output perturbations contract, showing stable computation embedded within near-chaotic dynamics.
(PDF)

S6 Fig Neural data simulation pipeline. We use a separate directory for task data used to generate simulated neural data (sim) compared to running task training (tt). The task data are loaded into a DataModule as a means of curating the trials passed through the trained model to obtain hidden-unit activations. In coupled environments, the TaskEnvironment is loaded to enable feedback at each step. Once all trials have been processed by the task-trained model, the hidden-unit activations are used by the Neural Data Simulator to produce simulated neural spiking activity. The simulated data are saved to dd, where they can be accessed later when running the data-training pipeline.
(PNG)

S7 Fig High-level schematic of data-training pipeline. The simulated neural data file is loaded from dd by the prepare_data() and setup() methods of the DataModule, and packaged into training and validation dataloaders. These dataloaders are used to train the data-driven model, an implementation of a LightningModule. The Trainer also accepts user-defined callbacks and loggers. After training is complete, the data-driven model and the corresponding datamodule are saved to /content/trained_models.
(PNG)

S8 FigDSA and fixed-point structure for NODE and GRU models across latent size (3BFF).**A)** DSA dissimilarity matrices comparing the ground-truth 3BFF TT model and data-driven NODE and GRU models across latent sizes, on a shared color scale. The boxed bottom row marks TT-vs-model dissimilarities, excluding the TT-vs-TT self-comparison. **B)** DSA dissimilarity to the TT model versus latent size for NODE and GRU models. **C)** Inferred fixed points over latent trajectories for the $\hat{D}=8$ and $\hat{D}=64$ NODE and GRU models. All model latents and fixed points are linearly projected onto the ground-truth 3-bit task state for a common orientation, and fixed points are computed under zero external input.(PDF)

S9 FigNonlinear cycle-consistency metric for DD-NODE models trained on the NBFF canonical dataset.**A)** nl_cycle_con curves (*R*^2^ vs. injected noise standard deviation) for DD-NODE models across latent sizes and random seeds. **B)** Scatter plot comparing nonlinear cycle consistency, evaluated at σ=0.05, against linear cycle consistency. **C)** Training diagnostics for the learned inverse MLP used by the metric: training and validation MSE loss versus epoch. Training and validation losses track closely across all latent sizes, indicating that the inverse mapping is not overfit.(PNG)

S10 FigValidation of the CtDToolkit Lyapunov-spectrum estimator on canonical chaotic systems.The full Lyapunov spectrum was estimated for the standard Lorenz and Rössler systems by integrating the vector field with a fourth-order Runge–Kutta scheme, building discrete-time Jacobians by finite differences, and applying compute_lyapunov_spectrum. Points and error bars show the mean ± s.d. of the estimated exponents across trajectories; red crosses show literature reference values. Panel titles report the maximal exponent $\lambda_{\max}$ and the sum $\sum_{i}\lambda_{i}$.(PDF)

S11 Fig Compiled metrics for DD-NODE models trained on the NBFF canonical dataset. **A)** Reconstruction metrics for DD-NODEs fit to the NBFF canonical dataset: Rate *R*^2^ and co-BPS. **B)** Simplicity metrics for the same models: State *R*^2^ and cycle consistency. **C)** Dynamical-systems-inspired metrics: Wasserstein distance and estimated maximal Lyapunov exponent.
(PNG)

S12 Fig Compiled metrics for DD-NODE models trained on the MultiTask canonical dataset. **A)** Reconstruction metrics for DD-NODEs fit to the MultiTask canonical dataset: Rate *R*^2^ and co-BPS. **B)** Simplicity metrics for the same models: State *R*^2^ and cycle consistency. **C)** Dynamical-systems-inspired metrics: Wasserstein distance and estimated maximal Lyapunov exponent.
(PNG)

S13 Fig Compiled metrics for DD-NODE models trained on the RandomTarget canonical dataset. **A)** Reconstruction metrics for DD-NODEs fit to the RandomTarget canonical dataset: Rate *R*^2^ and co-BPS. **B)** Simplicity metrics for the same models: State *R*^2^ and cycle consistency. **C)** Dynamical-systems-inspired metrics: Wasserstein distance and estimated maximal Lyapunov exponent.
(PNG)

S14 Fig Compiled metrics for LFADS models trained on the PhaseCodedMemory canonical dataset. **A)** Reconstruction metrics for LFADS models fit to the PhaseCodedMemory canonical dataset: Rate *R*^2^ and co-BPS. **B)** Simplicity metrics for the same models: State *R*^2^ and cycle consistency. **C)** Dynamical-systems-inspired metrics: Wasserstein distance and estimated maximal Lyapunov exponent.
(PNG)

S15 Fig Compiled metrics for DD-NODE models trained on the ChaoticDelayedMatching canonical dataset. **A)** Reconstruction metrics for DD-NODEs fit to the ChaoticDelayedMatching canonical dataset: Rate *R*^2^ and co-BPS. **B)** Simplicity metrics for the same models: State *R*^2^ and cycle consistency. **C)** Dynamical-systems-inspired metrics: Wasserstein distance and estimated maximal Lyapunov exponent.
(PNG)

S16 Fig Data-driven model validation on PhaseCodedMemory. Each row corresponds to one model: the ground-truth TT system and LFADS DD models of increasing latent dimensionality. Columns show firing-rate rasters, top PCs of response-period activity in firing-rate space, and top PCs of response-period activity in latent space. Reconstruction improves with latent size while State *R*^2^ degrades, and the DD models do not recover the qualitative phase-coding geometry of the TT model.
(PNG)

S17 Fig Objects for user-defined tasks and TT models. Required attributes and methods for implementing a user-defined TaskEnvironment and task-trained (TT) model.
(PNG)
