# Supplementary material for: Computation-through-Dynamics Toolkit: Simulated datasets and quality metrics for dynamical models of neural activity
Source: PLoS Comput Biol. 2026 Oct 15;22(10):e1014755. doi: 10.1371/journal.pcbi.1014755 (PMC13712701; doi:10.1371/journal.pcbi.1014755)
Supplement: Supplementary file 1 [file pcbi.1014755.s001.pdf]

S1 Text. Supplementary methods and implementation details

A Computation-through-Dynamics Toolkit:

We envision two classes of CtDToolkit users:

1. **DD model/metric developer:** This group will train their data-driven models on the canonical datasets and use the metrics provided to assess the model performance. We expect this user group to be primarily interested in adding their own data-driven models and metrics to the code-base, and less interested in modifying the existing tasks or simulating their own neural data. This group is referred to as "DD-Developers".

2. **TT model developer:** This group is primarily interested in adding their own tasks to the CtDToolkit and/or simulating neural data with different characteristics (noise models, observation models, etc.). We have provided a template class that these users can follow to incorporate new tasks into CtD, and describe the resources available to these users in Section F.1.

As described in Section 2 and shown in Fig 2A, CtD has three major components:

1. **Generating simulated neural datasets:** We provide the task environments and logic necessary to produce datasets from each of the tasks in CtD. We also include access to pre-trained models so that users can directly simulate neural data corresponding to these tasks.

2. **Training data-driven models:** We provide a pipeline for training neural dynamics models on simulated neural data produced by a task-trained model.

3. **Comparing model performance:** We provide several metrics for analyzing and comparing neural dynamics model performance, as well as the supporting code for easily logging and visualizing such results.

A.1 Overview of DD-Developer process flow

We provide five canonical datasets for DD-Developers. Each synthetic dataset was generated from a TT model trained to perform one of the included tasks. DD-Developers can get started with training their models on the canonical datasets by following a few simple steps:

1. **Clone the GitHub repository:** Navigate to our GitHub page and clone the repository. This repository includes task-training and data-training pipelines, comparison metrics, and five pre-trained models, which are used to generate the canonical datasets.

2. **Set up the environment:** Follow the instructions in the README to set up the environment for the CtD Toolkit.

3. **Generate the canonical datasets:** Users can generate the canonical datasets using pre-trained models for each task by running the script `/examples/gen_datasets.py`. This script generates the canonical datasets by 1) generating inputs and outputs from a saved task-environment, 2) running the pre-trained TT model on this dataset, 3) simulating neural activity from the hidden activity of the pre-trained model (saved in `/content/datasets/dd`). More information on the task-environments and task-training pipeline can be found in Section B.

4. **Train models:** Use `/examples/run_data_training.py` to train models on the canonical datasets. Users can choose the model class (LFADS, SAE), the canonical dataset (NBFF, MultiTask, RandomTarget, PhaseCodedMemory), and the input mode (supplied or inferred). Running this script without changing any parameters will start a run that will replicate the results from Fig 5. More information on data-driven models can be found in Section D.

5. **Compare trained models:** Once the models are done training, users can load the saved models into Analysis_DD objects (`/ctd/comparison/analysis/dd`), which can be compiled in a Comparison object (`/ctd/comparison/comparison.py`). The Comparison object provides useful tools for visualizing and computing metrics across trained models. More information on the available comparisons can be found in Section E, and a useful example notebook can be found at `/examples/compare_tt_dd_models.ipynb`.

B Canonical Datasets and Task Training:

In this section, we give details on the task environments, models and hyperparameters that were used to generate the pre-trained models for each task. We first describe the high-level Task Environment structure, and then describe how it was used for each canonical dataset. Information on using the pre-trained models to generate synthetic datasets is given in Section C. We summarize the training pipeline in S1 Fig.

B.1 Task Environments

We use the TaskEnvironment structure to generate the task-training dataset for the task-trained model. TaskEnvironments inherit from the Gymnasium.Environment class. Each TaskEnvironment defines the logic of a given task and implements the function `generate_dataset()`, which returns a dictionary that contains the inputs, outputs, and other information necessary to train a model to perform the task. For TT model developers who wish to modify or add TaskEnvironments, we describe more requirements for TaskEnvironments in Section F.1.

The `generate_dataset()` function is the heart of the TaskEnvironment. This function returns a dictionary with the following entries:

- `ics`: (shape: `n_samples` × `D`). The initial conditions of the task environment (e.g., joint angles at trial start, the state of the bits in 3BFF).
- `inputs`: (shape: `n_samples` × `timesteps` × `n_inputs_pre`). The inputs to the model that can be pre-computed (e.g., inputs pulses for 3BFF).
- `inputs_to_env`: (shape: `n_samples` × `timesteps` × `n_ext_inputs`). Any inputs that are applied to the environment rather than passed directly to the TT model. The bump perturbations in the `RandomTarget` task are the only examples of this type of input in the provided datasets.
- `targets`: (shape: `n_samples` × `timesteps` × `n_targets`). The target values for the TT model (e.g., the true state of the 3BFF).
- `true_inputs`: (shape: `n_samples` × `timesteps` × `n_inputs`). This contains the noiseless ground truth inputs for visualization and training.
- `conds`: (shape: `n_samples` × `1`). Condition indices for each trial. Used to re-weight the loss contributions of different trial types and for batching during training (used only in MultiTask).

Throughout, time is discretized into fixed-width *bins*: all per-trial quantities (inputs, outputs, latent states, and spikes) and all timings reported below are expressed in these bins, and the number of bins per trial is set by each task's `n_timesteps` parameter. Importantly, the task-trained (TT) model, the neural simulator, and the data-driven (DD) models all operate on this same binning—the simulator emits spikes on the bins of the TT latent trajectory, and DD models are fit to those spikes without any rebinning or resampling. The TT and DD models therefore use an identical time step within a given dataset. The physical duration of a bin is task-dependent. For tasks tied to a physical process we set an explicit bin width: 10 ms for RandomTarget (matching the MotorNet effector integration step), 20 ms for MultiTask, 2 ms for PhaseCodedMemory (a 0.8 s trial divided into 400 bins), and 1 ms for ChaoticDelayedMatching (so that the leaky-integration time constant $\tau = 30\,\text{ms}$ corresponds to the model's leak factor $\alpha = \mathrm{d}t/\tau = 1/30$). For 3BFF, the underlying dynamical system is scale-free, so time is expressed in dimensionless bins (equivalently, arbitrary time steps) with no physical unit.

Each TaskEnvironment also includes the loss function that is used to train models to perform the task. We describe the loss function for each of the canonical datasets in more detail below.

B.1.1 Three-Bit Flip-Flop (3BFF)

General Description The simplest of the canonical datasets is the 3BFF dataset, which is a 3 bit memory task described first in [1]. The task takes inputs $\mathbf{u}_t \in \mathbb{R}^3$ representing pulse signals on three channels and produces outputs $\mathbf{x}_t \in \mathbb{R}^3$ corresponding to the binary memory states of each channel. Our implementation can be found in `ctd/task_modeling/task_env/task_env.py` in the NBitFlipFlop class. Below, we describe the environment parameters and loss function:

Environment Parameters The NBitFlipFlop class takes the parameters described below. Values used in the canonical 3BFF dataset are given in Table S1.

- `n_timesteps`: The length of each trial in bins.
- `noise`: Standard deviation of Gaussian noise to be added to the inputs. This noise is pre-computed and the same every time a given trial is presented.
- `n`: The number of bits in the NBitFlipFlop (Default: 3).
- `switch_prob`: The probability of an input pulse on a given timestep.
- `transition_blind`: The number of bins after an input pulse that the loss is zeroed out (described in *Loss function* below).

Trial Structure Trials begin with all states set to zero. Each timestep has a small probability of a positive or negative input pulse on each channel. Positive inputs push the state from zero to one, negative inputs push the state from one to zero, while positive (negative) inputs have no effect when the state is one (zero). We show an example trial of 3BFF in S2 Fig.

Loss Function During training, we penalize the mean squared error between the model output and the desired output. To avoid interference from initial transient effects, we mask the losses during the first 5 bins of each trial. Additionally, we apply a masking period (default: 4 bins, defined by `transition_blind`) following each input pulse. We found that this masking helped the model learn smoother dynamics that cleanly transitioned from one state to another without large discontinuous jumps in the state. See `/ctd/task_modeling/task_env/loss_func.py` for full implementation details.

$$\mathcal{L}_{3BFF} = \frac{1}{T}\sum_{t=6}^{T} \mathbf{M}_t \cdot ||\mathbf{x}_t - \hat{\mathbf{x}}_t||^2$$

where $\mathbf{x}_t$ is the target (desired) output and $\hat{\mathbf{x}}_t$ is the model-predicted output at time t, T is the trial length in bins, and $\mathbf{M}_t$ is the masking function, where t_{pulse} denotes the time (in bins) of an input pulse:

$$\mathbf{M}_t = \begin{cases} 0 & \text{if } t \leq 5 \text{ or } t \in [t_{pulse}+1, t_{pulse}+4] \\ 1 & \text{otherwise} \end{cases}$$

Task-Trained Model (NoisyGRU) For the canonical 3BFF dataset, we used a modified Gated Recurrent Unit (GRU) network called a NoisyGRU. NoisyGRU adds a configurable intrinsic noise to the state at each time step and to the initial hidden state of the network on each trial. The noise at each time step was drawn from a standard normal distribution with mean zero and isotropic variance of 0.01, while the initial conditions were drawn from a standard normal with mean μ and variance of 0.05, where μ was a trainable parameter. This ensured that the model learned to stably encode memory regardless of semi-random initial conditions. Full hyperparameters are in Table S2. We provide the NoisyGRU model, fully-trained on 3BFF, in the `content/trained_models/task-trained/tt_3bff/` folder of the CtDToolkit repository.

The full state-update equations of the NoisyGRU are:

$$\mathbf{h}_0 = \mu + \eta; \quad \eta \sim \mathcal{N}(0, 0.05) \tag{1}$$
$$\mathbf{z}_t = \sigma(\mathbf{W}_z \mathbf{u}_t + \mathbf{U}_z \mathbf{h}_{t-1} + \mathbf{b}_z) \tag{2}$$
$$\mathbf{r}_t = \sigma(\mathbf{W}_r \mathbf{u}_t + \mathbf{U}_r \mathbf{h}_{t-1} + \mathbf{b}_r) \tag{3}$$
$$\tilde{\mathbf{h}}_t = \tanh(\mathbf{W}_h \mathbf{u}_t + \mathbf{U}_h (\mathbf{r}_t \odot \mathbf{h}_{t-1}) + \mathbf{b}_h + \xi_t); \quad \xi_t \sim \mathcal{N}(0, 0.01) \tag{4}$$
$$\mathbf{h}_t = (1 - \mathbf{z}_t) \odot \mathbf{h}_{t-1} + \mathbf{z}_t \odot \tilde{\mathbf{h}}_t \tag{5}$$

Where $\mathbf{u}_t$ is the task input at time step t, $\mathbf{h}_0$ is the initial hidden state with noise, $\mathbf{z}_t$ is the update gate at time step t, $\mathbf{r}_t$ is the reset gate at time step t, $\tilde{\mathbf{h}}_t$ is the candidate hidden state at time step t, $\mathbf{h}_t$ is the final hidden state at time step t, η and ξ_t are the noise terms drawn from normal distributions, $\sigma(\cdot)$ is the sigmoid function, and $\odot$ represents the element-wise (Hadamard) product. The trainable parameters are the input weight matrices $\mathbf{W}_z, \mathbf{W}_r, \mathbf{W}_h$, the recurrent weight matrices $\mathbf{U}_z, \mathbf{U}_r, \mathbf{U}_h$, the gate biases $\mathbf{b}_z, \mathbf{b}_r, \mathbf{b}_h$, and the mean initial state μ.

B.1.2 MultiTask

General Description Our dataset of "intermediate" difficulty was generated using the previously described MultiTask task [2, 3], which is composed of 15 cognitive tasks that have overlapping task requirements (e.g., memory, directional responses, multiple stimulus modalities). To perform MultiTask, a model takes inputs $\mathbf{u}_t \in \mathbb{R}^{20}$ (1 fixation input, 2 inputs for stimulus modality 1, 2 inputs for stimulus modality 2, and 15 one-hot task inputs) and produces outputs $\hat{\mathbf{x}}_t \in \mathbb{R}^3$ (1 fixation, 2 response channels). The tasks can be broken down into three categories: *response*, *decision making*, and *matching*.

Response: During Response tasks, inputs were received on the 2 input dimensions associated with stimulus modality 1, and the model was asked to respond by producing output either in the direction of the stimulus ("Pro") or in the opposite direction ("Anti"). Different tasks required that the model respond at different points during the trial; for "React", the model was trained to respond immediately upon receiving the stimulus. For "Delay", the model was trained to respond after a short delay period in which the model continues to see the stimulus. For "Memory", the model was trained to respond after a short period when the stimulus was turned off upon receiving a go-cue. With "Pro/Anti" versions of the "React/Delay/Memory" tasks, there are 6 total tasks in the Response category.

Decision Making: Decision Making tasks were designed to emulate systems that make decisions about the content of noisy inputs. In one version of the task ("IntMod"), we presented two stimuli to a single input modality, and the model was trained to move in the direction of the larger magnitude stimulus. In a second version ("ContextInt"), we presented stimuli to both modalities in each stimulation period, and models were trained to ignore one of the modalities based on the rule input. In the final version of the task, the model was trained to incorporate information from both modalities equally before deciding which was larger ("MultiModal"). With "IntMod1", "IntMod2", "ContextInt1", "ContextInt2", and "ContextIntMultimodal", there were a total of 5 decision making tasks included in MultiTask.

Matching: The final class of tasks are Matching tasks. In two of these tasks, the model must produce output that indicates whether consecutive stimuli provided to the first input modality were the same direction ("Match") or opposite direction ("Non-Match") of one another, responding to the second stimulus location if so. The final two Matching tasks ("MatchCat", "MatchCatAnti") required the model to respond only if both stimuli took on values in the range $[0, \pi)$ or $[\pi, 2\pi)$.

We show an example trial of each task in S3 Fig, grouped by the task type. Lengths of each phase of the tasks are drawn from a min and max length shown in Table S3. Additional implementation details are drawn from [3]. Full task code can be found at `/ctd/task_modeling/task_env/multitask.py`.

Environment Parameters The MultiTask class takes the parameters described below. The values for each parameter used when generating the canonical Multitask dataset are given in Table S4.

- `n_timesteps`: The maximum length across trials in bins.
- `noise`: Standard deviation of Gaussian noise to be added to the inputs.
- `num_targets`: The number of targets in the task (distributed uniformly on the circumference of the unit circle.)

Of note, all trials were 320 bins long (the longest possible trial given the lengths in Table S4). When feeding trials to our task-trained model, we simulated for the full 320 bins, then truncated the trials to the appropriate length for all subsequent loss computation and analysis.

Trial Structure Each MultiTask trial proceeds through up to five epochs—a context (fixation) period, a first stimulus (Stim1), a memory delay (Mem1), a second stimulus (Stim2), and a response period—although which epochs are present and their durations depend on the task type. The duration of each epoch is drawn per trial from the [min, max] ranges in Table S3, where a zero range indicates that the epoch is absent for that task type.

Loss Function The MultiTask loss function was to minimize the MSE of the task-trained model outputs against the desired outputs. Because trials were of variable length, we masked the MSE loss to only include time-bins that are within each trial's total length (i.e., the sum of the randomly drawn intervals from Table S3). We also masked the loss to exclude contributions from the first 5 bins of each trial, and the first 5 bins after the response period begins. Additionally, we re-weighted the loss contribution of the response period so that performance during this period was weighted 5 times more than the rest of the trial. This was done in accordance with [3] to help with training speed and performance.

We computed the performance of our task-trained models using the criteria described in [3], which finds the percentage of trials where the model responds in the appropriate direction (i.e. the angle was correct to within an error of $< \pi/10$ radians), or on trials without a desired response, that the model does not respond. As with the previous work, we found that our TT models consistently achieved performance that exceeded 80% success rate on the validation dataset for all tasks.

$$\mathcal{L}_{MultiTask} = \frac{1}{|T_{valid}|} \sum_{t \in T_{valid}} \mathbf{W}_t \cdot ||\mathbf{x}_t - \hat{\mathbf{x}}_t||^2 + \lambda_{L2} ||\mathbf{h}_t||^2$$

where:

$$\mathbf{W}_t = \begin{cases} 0 & \text{if } t \leq 5 \text{ or } t \in \text{first 5 bins of response period} \\ 5 & \text{if } t \in \text{response period} \\ 1 & \text{otherwise} \end{cases}$$

and T_{valid} includes only time-bins within each trial's actual length, $\mathbf{h}_t$ is the model's hidden activity, and $\lambda_{L2} = 10^{-6}$.

Task-Trained Model (NoisyGRU) For our canonical MultiTask dataset, we used a NoisyGRU (described in Section B.1.1) with the hyperparameters shown in Table S5. Training batches were composed of trials from only one task. We also added a loss term that penalized the squared hidden activity that pressured activity to live near the origin, leading to simpler fixed-point structures (similar to a loss contribution used in [3]). Relevant hyperparameters for the canonical Task-trained MultiTask model are given in Table S5.

B.1.3 RandomTarget

General Description In the RandomTarget task, a TT model is trained to control a 2 degree-of-freedom arm model actuated by 6 Hill-type muscles (analogous to pectoralis, deltoid, triceps long, triceps lat, biceps, and brachialis). By controlling the muscle activations $\mathbf{a}_t \in \mathbb{R}^6$ of the arm model, the TT model must move the effector endpoint to various target locations sampled randomly from within the arm's range of motion. TT models trained on this task receive inputs $\mathbf{u}_t \in \mathbb{R}^{17}$ consisting of both sensory (muscle lengths and velocities, hand endpoint position; $\mathbf{u}_t^{sens} \in \mathbb{R}^{14}$) and contextual (target position, go cue; $\mathbf{u}_t^{ctx} \in \mathbb{R}^3$) information.

Environment Parameters We chose parameters similar to those used in [4] for the two-link arm model. We summarize the parameters in Table S6. So that the model couldn't anticipate the go cue and reach prior to the end of the delay period, 20% of training trials were "catch" trials in which the go-cue was never provided. During training the timing of the target presentation and go cue were random (target presentation occurred sometime in an interval of [100, 300] ms after trial start and the go cue occurred randomly between 50 ms after target presentation and the end of the trial, which lasted a total of 1.55 seconds). When simulating the neural data, the target onset and go-cue were consistent across trials (300 and 700 ms after the start of the trial, respectively), but bump timing was still random. For consistency with typical neural datasets of this type, our simulated data had consistent timings so that the go-cue occurred at a consistent alignment point rather than at a random point in the trial. When we simulated the data, we trimmed the first 5 bins of each trial to ensure that the activity of the network was stable at the starting location before the reaching behavior began.

We briefly describe important parameters of the RandomTarget task here:

- `n_timesteps`: The length of each trial in bins.
- `action_noise`: Standard deviation of Gaussian noise added to the actions output by the model (i.e., motor noise).
- `proprioception_noise`: Standard deviation of Gaussian noise added to the proprioceptive feedback (muscle lengths and velocities)
- `vision_noise`: Standard deviation of Gaussian noise added to the visual feedback (endpoint position)
- `context_input_noise`: Standard deviation of Gaussian noise added to the context inputs (target position and go-cue).
- `proprioception_delay`: Simulated conduction delay of proprioceptive feedback (bins)
- `vision_delay`: Simulated conduction delay of visual feedback (bins)

Trial Structure Each trial began with the effector endpoint at a random location in the workspace (Fig 3G), and was composed of 3 phases 1) Context: the model held at the start location. 2) Delay: A target position (x/y) was presented to two of the context inputs of the task-trained model. 3) Reach: The target information was removed and a step-function go-cue was presented, cueing the model to reach to the target location and hold there until the end of the trial. Because information about the target location was not present after the go-cue, the TT model was obligated to remember the target location while making the reach. Additionally, we applied external bump perturbations to the hand at random times in the trial, which were randomly directed and of a variable magnitude. This resulted in reaches with and without corrective movements, thereby increasing the diversity and nature of computation required to complete the task successfully.

Loss Function We trained TT models to minimize the mean squared error between the effector endpoint and the desired endpoint (i.e. the start location prior to the go-cue, and the target location after the go cue). To encourage strategies with minimal energy expenditure, the TT model was also trained to minimize an L_2 norm of the muscle activations. To improve training stability, we incrementally increased the window of loss included in the training. After 200 epochs, the entire trial contributed to the training loss equally.

$$\mathcal{L}_{RandomTarget} = \lambda_{position}\mathcal{L}_{position} + \lambda_{energy}\mathcal{L}_{energy}$$

where:

$$\mathcal{L}_{position} = \frac{1}{T}\sum_{t=1}^{T}||\mathbf{p}_t - \hat{\mathbf{p}}_t||^2$$

$$\mathcal{L}_{energy} = \frac{1}{T}\sum_{t=1}^{T}||\mathbf{a}_t||^2$$

where $\mathbf{p}_t$ is the effector endpoint position, $\hat{\mathbf{p}}_t$ is the desired endpoint position (start location before the go-cue, target location after), and $\lambda_{position}$ and λ_{energy} are the weights on the position and energy terms, both set to 1.0 for the canonical task-trained model.

Task-Trained Model (NoisyGRU) For the canonical RandomTarget dataset, we used a NoisyGRU model (described in Section B.1.1) with the hyperparameters shown in Table S7. As in Section B.1.2, we also penalized the L_2 norm of the hidden activity of this model to force the activity to live closer to the origin, which produces more interesting dynamical structures.

B.1.4 PhaseCodedMemory

General Description A second simple task included in CtDToolkit is the PhaseCodedMemory task, based on recent work investigating short-term memory in the hippocampus [5]. The task receives inputs $\mathbf{u}_t \in \mathbb{R}^3$ consisting of a reference oscillation and two stimulus channels, and produces a scalar output $x_t \in \mathbb{R}$ representing the phase-shifted oscillation encoding stimulus identity. Our implementation can be found in `ctd/task_modeling/task_env/phase_coded_memory.py` in the PhaseCodedMemory class. In S4 Fig we show the structure of inputs and outputs of the model in more detail:

Environment Parameters The PhaseCodedMemory class takes the parameters described below. Values used in the canonical PhaseCodedMemory dataset are given in Table S8.

- `n_timesteps`: The length of each trial in bins.
- `noise`: Standard deviation of Gaussian noise to be added to the inputs. This noise is pre-computed and the same every time a given trial is presented.
- `post_stim_l2_weight`: Scale of L2 penalty applied to the latent activity (required for model to converge to phase-coding strategy).
- `lat_l2_full`: Boolean. Whether to apply the latent L2 to the full trial, or just the post-stimulus period.
- `pred_loss_full`: Boolean. Whether to apply the prediction loss to the full trial or just to the post-stimulus period.

Trial Structure Trials begin with the reference oscillation with a initial phase drawn from a uniform random distribution from $[0, 2\pi]$. This oscillation is provided to the TT model on input channel 1. After a delay (drawn from a uniform random distribution from $[60, 120]$ bins from the start of the trial), a stimulus appears on either input channel 2 or 3 (Stim A, B, respectively). This stimulus is maintained for a duration drawn from a uniform random distrubtion ranging from $[60, 80]$ bins. We show example trials of PhaseCodedMemory in S4B Fig.

Loss Function During training, we penalize the mean squared error between the model output and the desired output in the period after the stimulus offset. Additionally, as previous research found that full-rank RNN models require regularization pressure to converge to a phase-coding solution, we applied an L2 penalty to the latent activity in the post stimulus offset window (see `post_stim_l2_weight`). See `/ctd/task_modeling/task_env/loss_func.py` for full implementation details.

$$\mathcal{L}_{PCM} = \mathcal{L}_{pred} + \lambda \mathcal{L}_{reg}$$

where:

$$\mathcal{L}_{pred} = \frac{1}{|T_{post}|} \sum_{t \in T_{post}} ||x_t - \hat{x}_t||^2$$

$$\mathcal{L}_{reg} = \frac{1}{|T_{post}|} \sum_{t \in T_{post}} ||\mathbf{h}_t||^2$$

and T_{post} represents the post-stimulus period, $\mathbf{h}_t$ is the model's latent activity, and λ = `post_stim_l2_weight`.

Task-Trained Model (NoisyGRU) For the canonical PhaseCodedMemory dataset, we used the same class of NoisyGRU as for the 3BFF task. The noise at each time step was drawn from a standard normal distribution with mean zero and isotropic variance of 0.01, while the initial conditions were drawn from a standard normal with mean μ and variance of 0.05, where μ was a trainable parameter. This ensured that the model learned to stably encode memory regardless of semi-random initial conditions. Full hyperparameters are in Table S9. We provide the NoisyGRU model, fully-trained on PhaseCodedMemory task, in the `content/trained_models/task-trained/tt_PhaseCodedMemory/` folder of the CtDToolkit repository.

B.1.5 ChaoticDelayedMatching

General Description ChaoticDelayedMatching extends CtDToolkit to the near-chaotic dynamical regime while retaining a well-specified computation. The task is a delayed non-match-to-sample paradigm based on [6]. The task receives inputs $\mathbf{u}_t \in \mathbb{R}^2$ consisting of two cue channels (Cue A and Cue B) and produces a scalar output $x_t \in \mathbb{R}$ that reports, during a response window, whether the two cues presented on a given trial matched (A-A or B-B, target -1) or differed (A-B or B-A, target $+1$). Our implementation can be found in `/ctd/task_modeling/task_env/chaotic_delayed_matching.py` in the ChaoticDelayedMatching class. In S5 Fig we show the task structure and the chaotic signatures of the task-trained dynamics.

Environment Parameters The ChaoticDelayedMatching environment takes the following parameters (canonical values in parentheses):

- `n_timesteps`: total trial length in bins (1050).
- `noise`: standard deviation of the Gaussian noise added to the two cue input channels (0.1).
- `cue_scale`: amplitude of the cue pulses (1.0).
- `baseline_range`, `cue1_range`, `delay1_range`, `cue2_range`, `delay2_range`, `response_range`: the [min, max] duration (in bins) of each trial epoch, drawn per trial; for the canonical dataset these are fixed at 50, 200, 200, 200, 200, and 200 bins, respectively.

Trial Structure Each trial lasts 1050 bins (1 ms per bin) and is divided into six contiguous epochs: a baseline period (50 bins), the first cue (200 bins), a first delay (200 bins), the second cue (200 bins), a second delay (200 bins), and a response period (200 bins). On each cue epoch, a pulse is presented on either the Cue A or Cue B channel. Gaussian input noise (standard deviation 0.1) is added to the cue channels. We show example trials of ChaoticDelayedMatching in S5A Fig.

Loss Function During training, we penalize the mean absolute error between the model output and the target value, computed on the trial-averaged output within the response window. See `/ctd/task_modeling/task_env/loss_func.py` (ChaoticDelayedMatchingLoss) for full implementation details.

Task-Trained Model (ChaoticRateRNN) In contrast with the NoisyGRU used for the other canonical datasets, ChaoticDelayedMatching is solved by a leaky-integration tanh rate RNN (ChaoticRateRNN) whose recurrent weights are initialized with a gain $g = 1.5$, placing the autonomous network in a near-chaotic regime [7]. The model has a latent size of 200, a leak factor $\alpha = 1/30$, no bias terms, and fixed (non-trained) random input weights; only the recurrent weights and the linear readout are trained. We simulate neural activity from 96 units (64 held-in, 32 held-out) using an exponential rectifying nonlinearity and pseudo-Poisson observation noise. We provide the task-trained model in the `/pretrained/20260320_ChaoticDelayedMatching_Final/` folder of the CtDToolkit repository.

C Neural Data Simulation

Here we provide additional details on the process and implementation of the neural data simulation pipeline and provide a schematic in S6 Fig.

C.1 Simulator

Sampling simulated neurons Synthetic neural data used in CtDToolkit is simulated from hidden unit activity of a task trained model. Initially, the requested number of simulated neurons is assigned to hidden units of the task-trained model via a one-hot readout matrix. When the requested neuron count N does not exceed the latent dimensionality D, units are sampled without replacement (one neuron per latent dimension). When $N > D$, the readout matrix is built by stacking $\lceil N/D \rceil$ independent random permutations of the latent indices and retaining the first N columns, so that some latent dimensions are observed by more than one simulated neuron. Because each simulated neuron is then passed through the observation nonlinearity and sampled independently (e.g. as an independent Poisson draw), these shared latent dimensions yield distinct noisy "views" of the same underlying rate rather than identical duplicate neurons. This is why, for example, the PhaseCodedMemory dataset can supply $500 + 100 = 600$ simulated neurons (Table S10) from a task-trained model with $D = 512$ latent units: with $\lceil 600/512 \rceil = 2$ stacked permutations, the first 600 of 1024 indices are retained, so 88 latent dimensions are observed by two neurons each.

Firing-rate normalization The activity of these sampled units is then standardized using one of two procedures. By default, we compute a *single* global mean and a *single* global standard deviation across all trials, time points, and sampled units, and transform the activity by subtracting the global mean and dividing by the product of `fr_scaling` and the global standard deviation. Because every unit is normalized by the same two scalars (rather than by unit-specific statistics), this transformation does not alter the relative magnitudes of activity across units inherited from the latent states of the TT model. Alternatively, when `target_mean_rates` is specified, the global normalization is skipped; instead, per-unit additive biases are fit so that, after the rectifying nonlinearity, each unit attains a specified target mean firing rate (this pathway is used here only for the PhaseCodedMemory dataset, for which we targeted low average firing rates).

Spike generation The activity is then rectified and used to define a stochastic process from which spiking events are sampled over the course of the trial. For all of our experiments, the simulated spiking data was sampled from a Poisson process, but for flexibility we include options for sampling from distributions that are under-dispersed or over-dispersed compared to Poisson, such as the Binomial and Negative Binomial distributions, respectively.

Held-out neurons The co-smoothing bits-per-spike metric requires a set of held-out neurons in addition to a held-in set. The number of held-out neurons varies by dataset—for example, 10 for 3BFF, MultiTask, and RandomTarget, and 100 for PhaseCodedMemory (Table S10)—each sampled from the TT network in addition to the held-in set for computing co-BPS.

The neural data simulator is called after the TT model is trained, as part of the Task Training Pipeline (more information in Sections B and F). The Simulator has parameters that control the number of artificial neurons to sample, firing rate scaling, and how hidden activity is embedded into neural rates. Parameters for the neural data simulator across tasks can be seen in Table S10.

The general API for the Simulator is as follows:

Variables:

- `neuron_dict`: dictionary containing arguments about how many neurons should be in the held-in/held-out datasets.
  - `n_neurons_heldin`: Number of held-in neurons to feed into DD models
  - `n_neurons_heldout`: Number of held-out neurons to be predicted by the DD model (in addition to held-in).
- `embed_dict` : dictionary containing arguments related to how the hidden unit activity of the task-trained model is processed into firing rates. Example keys include:
  - `rect_func` : The type of function to use for rectifying the hidden unit activity (e.g. The rate parameter of the Poisson distribution must be greater than zero.)

- `fr_scaling` : Scaling factor applied to firing rates. Higher values of `fr_scaling` lead to lower firing rates.
- `target_mean_rates` : (optional) Alternative to fr_scaling, in which firing rates are optimized such that the mean firing rate of the neurons match a desired target.

- `noise_dict` : dictionary containing arguments related to how spiking events are sampled from the simulated firing rates. Example keys include:
  - `obs_noise` : *String*. For all datasets included in CtDToolkit, we used a generalization of the Poisson distribution that considers the dispersion of the probability density function, controlled by a dispersion factor given by `dispersion`.
  - `dispersion` : Parameter controlling the level of dispersion of the probability density function. For `dispersion` = 1.0, the distribution is Poisson.

Methods:

`generate_simulated_data()`: Uses task-trained model to generate simulated neural responses, as well as collect additional information about the task and trials from the datamodule used to train the TT model.

Arguments:

- `task_trained_model` : Task Wrapper (described in Section F) object for the TT model after it has been fit to the task data from `datamodule`.
- `datamodule` : PyTorch Lightning Data Module for serving the task input/output data to the TT model.
- `seed` : Makes data generation deterministic for reproducibility (e.g. sampling which TT model hidden units to turn into neurons, sampling of spiking events, etc.)

`simulate_neural_data()`: Calls `generate_simulated_data()`, compiles all fields relevant for data-driven modeling, and saves them as HDF5 files.

Arguments:

- `task_trained_model` : Task Wrapper (described in Section F) object for the TT model after it has been fit to the task data from `datamodule`.
- `datamodule` : PyTorch Lightning datamodule for serving the task input/output data to the TT model.
- `run_tag` : filename for saving the simulated data to the project, written in `/examples/run_task_training.py`
- `subfolder` : optional subfolder to save data somewhere more specific inside the project, specified by overrides passed to the `train()` function in `ctd/task_modeling/task_train_prep.py`
- `dataset_path` : highest level save path, acquired from `path_dict` in `examples/run_task_training.py`

Note that the majority of the above arguments are set at a higher level in the task training scripts and passed through to the simulator. Any direct interactions with the simulator are via configuration files.

D Utilizing the Data-Training Pipeline

D.1 Training Pipeline Overview

The data training pipeline allows users to deploy models of neural dynamics on the simulated neural data produced by task-trained models. Our design allows for modular training and saving of data-driven model checkpoints and performance metrics, and supports end-to-end model training using PyTorch Lightning.

We use hydra [8] to instantiate configuration files for default model parameters, and Ray-tune [9] for hyperparameter tuning. We recommend using the template provided in `ctd/data_modeling/` for adding new data-driven models or modifying existing models. See Section D.2 for details and see S7 Fig for a schematic.

A basic DD user can begin training data-driven models on the canonical datasets using the script `/examples/run_data_training.py`. At the top of this script, users must select from the following choices:

1. Choose the data-driven model class (SAE or LFADS).

2. Choose a model architecture
(from `/ctd/data_modeling/configs/{MODEL_CLASS}/{MODEL}`)

3. Choose a canonical dataset (options are "NBFF", "PhaseCodedMemory", "MultiTask", or "RandomTarget").

4. Select whether to infer inputs or supply them to the model (`INFER_INPUTS` flag).

For hyperparameter sweeping, users can modify variables in `SEARCH_SPACE` dictionary to perform hyperparameter sweeps (e.g., `tune.grid_search`) on model parameters. See [9] for additional choices for hyperparameter sweeping.

D.2 Custom DD Model Interface

To make the addition of user-defined models more straightforward and streamlined, we provide a template class called `TemplateSAE` which can be found at `ctd/data_modeling/models/SAE/template.py`. This template inherits from the Lightning Module object in PyTorch Lightning, and has the following methods and required named outputs:

Required Methods:

- `forward(data,inputs)` : Defines the series of model operations that act on the data in an end-to-end pass through of the model. Outputs `log-rates` and `latents`.

- `configure_optimizers()` : Handles instantiation of optimizer. This includes determining how different subsets of parameters are trained, as defined by dictionary arguments passed to the user's optimizer of choice. Must return a PyTorch `Optimizer` object.

- `training_step(batch, batch_idx)` : Definition of the entire end-to-end pass-through of the model, including the computation of the loss and logging of any intermediate metrics at each training step. Receives batches of data from the training dataloader defined in the `datamodule`. Outputs loss on training set for the current step.

- `validation_step(batch, batch_idx)` : Definition of how the model is evaluated on a validation set. This method often parallels `training_step()`, except no gradients are computed. Receives batches of data from the training dataloader defined in the `datamodule`. Outputs loss on validation set for the current step.

This class parallels the SAE baseline, defining all required methods and named outputs in a manner consistent with the rest of the toolkit. Users interested in defining their own models can fill out this template, including any additional custom methods as needed. Provided the user adds the corresponding config files to their repository (mentioned above in Section D.1), they should be able to directly train and analyze their models using the CtDToolkit infrastructure!

D.3 Baseline Models

As part of CtDToolkit, we provide implementations and/or comparisons for several baselines from across the literature. In the following subsections, we give brief descriptions of each approach, how hyperparameters were selected, and further motivation as to why each method was considered for CtDToolkit.

D.3.1 Model Class: Generic Sequential Autoencoder (SAE)

Gated Recurrent Unit (GRU) Our implementation of a GRU SAE can be found at `ctd/data_modeling/models/SAE/dyn_models_GRU.py`. It consists of the following components:

- **Encoder:** A bidirectional GRU processes input sequences to produce hidden states, capturing information from both past and future sequences.

- **Dropout Layers:** Applied to hidden states and initial conditions to prevent overfitting.
- **Initial Condition Linear Layer:** Maps concatenated hidden states from encoder GRU to initial condition for the decoder GRU.
- **Decoder:** An RNN with a GRU cell processes initial conditions and input sequences to produce latent states for each trial.
- **Readout Layer:** A linear map from the latent state $\mathbf{z}_t$ to per-neuron log-firing-rates, $\log \hat{\boldsymbol{\lambda}}_t = \mathbf{W}_{\text{out}}\mathbf{z}_t + \mathbf{b}_{\text{out}}$. Firing rates are then obtained by exponentiating, $\hat{\boldsymbol{\lambda}}_t = \exp(\log \hat{\boldsymbol{\lambda}}_t)$, which guarantees non-negativity (the canonical linear–exponential observation model).

The forward pass involves:

1. Passing the data through the encoder to get hidden states.
2. Applying dropout and linear mapping hidden states to the latent space.
3. Using the decoder to produce latent states from initial conditions and supplied inputs.
4. Mapping latent states to log-rates via the linear readout layer, then exponentiating to obtain non-negative firing rates.

The model uses a Poisson negative log-likelihood loss function to compute the difference between these predicted rates and the target spikes, with the potential for alternative loss contributions such as weight decay, etc. The Adam optimizer is used for training, with learning rate adjustments and weight decay to regularize the model.

Linear Dynamical System (LDS) We used a simplified implementation of an LDS to illustrate the utility of the metrics included in CtDToolkit. For this, we replaced the GRU cell from Section D.3.1 with a cell that could only model simple linear dynamics, according the equations:

$$h_t = Ah_{t-1} + Bu_t + b_h + b_x \tag{6}$$

Where:

- h_t is the hidden state at time step t.
- h_{t-1} is the hidden state at the previous time step.
- u_t is the input at time step t.
- A is a trainable weight matrix for the hidden state update.
- B is a trainable weight matrix for the input affecting the hidden state.
- b_h is a trainable bias vector for the hidden state update.
- b_x is a trainable bias vector for the input affecting the hidden state.

Neural ODE (NODE) Our implementation of a SAE class based on Neural Ordinary Differential Equations (Neural ODEs [10]) can be found at `ctd/data_modeling/models/SAE/dyn_models.py`. It consists of the following components:

- **Encoder:** A bidirectional GRU processes input sequences to produce hidden states, capturing information from both past and future sequences.
- **Dropout Layers:** Applied to hidden states and initial conditions to prevent overfitting.
- **Initial Condition Linear Layer:** Maps concatenated hidden states from encoder to initial condition for the decoder.

- **Decoder:** An RNN with a Neural ODE cell processes initial conditions and input sequences to produce latent states. We describe the NODEcell in more detail below.
- **Readout Layer:** Linear mapping from latent states to output data dimensions.

The equations that define the state update for the NODE cell are:

$$h_{t+1} = h_t + \beta f(h_t, u_t) \tag{7}$$

Where:

- h_t is the hidden state at time step t.
- u_t is the input at time step t.
- f is a function representing the change in hidden state, modeled by a Multilayer Perceptron (MLP) with a total of `vf_num_layers` layers that have `vf_hidden_size` hidden units each, and uses ReLU activations between layers.
- β is a scalar that improves training stability (set to 0.1).

Unlike typical Neural ODE models, we did not backpropagate the gradients through an ODE solver; instead, we did single-step Euler updates and used standard backpropagation-through-time to train the model. This approach has been shown to produce similar results on synthetic neural datasets as the ODE-solver-based training [11, 12]. Both forward pass and training procedure are the same as in the GRU model (Section D.3.1).

Hyperparameters for the NODE-based SAEs shown in Fig 5C can be found in Table S11.

D.3.2 Model Class: Latent Factor Analysis via Dynamical Systems (LFADS)

Latent Factor Analysis via Dynamical Systems (LFADS) [13] is a popular model that uses a latent dynamics model to reconstruct neural activity. LFADS is a sequential autoencoder that uses variational training to learn the dynamics that generate a set of spiking observations. LFADS uses the same reconstruction objective as the SAE models in Section D.3.1, except LFADS also penalizes the KL divergence of the initial condition (IC) encoder against a Gaussian prior. For the CtD Toolkit we used an open source implementation of LFADS [14] for its modularity and extensibility, which we consider desirable to have compatible with CtDToolkit and available for users as part of the toolkit. Full implementation details are given in [14].

For variants of the SAE that are used to infer inputs, there is an additional bidirectional GRU encoder (referred to as the controller-input encoder, or CI Encoder) that outputs a sequence that is passed to another GRU referred to as the "controller". The controller is responsible for producing time-varying inputs to the generator based on feedback from the generator at the preceding timestep, and the output of the CI encoder. The weights of the controller and CI encoder are updated to optimize the same loss as the rest of the components of LFADS, while also being directly penalized by the KL divergence against a user-chosen prior distribution. In the experiments shown in Fig 6, we use a Student's-t distribution ($df = 5$) to encourage the controller to infer sparse inputs. Alternative choices supported in CtDToolkit include an Autoregressive Gaussian as well as standard Gaussian priors. Custom priors can be easily added by users. In total, the loss for training an LFADS model in CtDToolkit is given by $\mathcal{L}$ in Equation 8 as follows:

$$\mathcal{L} = \mathcal{L}_{\text{Poisson}} + \lambda_{\text{con}}\mathcal{L}_{\text{con}}^{\text{KL}} + \lambda_{\text{IC}}\mathcal{L}_{\text{IC}}^{\text{KL}} \tag{8}$$

Here, $\mathcal{L}_{\text{Poisson}}$ is the Poisson negative log-likelihood reconstruction loss, $\mathcal{L}_{\text{con}}^{\text{KL}}$ and $\mathcal{L}_{\text{IC}}^{\text{KL}}$ are the KL-divergence penalties on the controller and initial-condition (IC) encoders against their priors, and λ_{con} and λ_{IC} are their respective weights, with $\lambda_{\text{IC}} = 0$ when inputs were supplied. We considered the LFADS model class as a baseline due to its customizability, enabling researchers to have an accessible but flexible model available for their initial experiments and to use for gaining intuition about Computation through Dynamics.

We provide hyperparameters for the LFADS models included in Fig 6 in Table S12. The "KL Penalty" in Section 3.3 and Fig 6 refers to the parameter `kl_co_scale`, where we tested how varying this parameter affected the quality of inferred dynamics.

We also include an LFADS variant that uses a NODE generator in place of the standard GRU RNN. NODE-based SAEs have been found to improve the ability of dynamics models to infer accurate latent activity [12, 15]. This model provides users with an experimental model with which they can begin probing how the generator might affect performance on the included CtDToolkit metrics. Switching from GRU to NODE-based LFADS is controlled by the gen_type parameter, which takes in a string (default: "RNN"). Changing this parameter to "NODE" changes the generator class to a Neural ODE. We provide a default configuration file (ResLFADS – for residual LFADS) so that users can easily test the effects of a NODE-based generator on the canonical datasets.

Low firing-rate cold start (PhaseCodedMemory). By default, the LFADS rate readout uses a fan-in linear layer initialized with zero bias and Gaussian weights of standard deviation $1/\sqrt{d_{\text{fac}}}$. Under the Poisson exp-link, this produces an initial predicted rate of ~ 1 spike/bin per neuron with a log-rate standard deviation of ~ 1 nat. For high firing-rate datasets (e.g. 3BFF, MultiTask, RandomTarget, ChaoticDelayedMatching) this initialization is well-conditioned. For low firing-rate datasets (e.g. PhaseCodedMemory, which simulates hippocampal CA1 neurons at $\sim 2\,\text{Hz}$; $\sim 4 \times 10^{-3}$ spikes/bin) it places the network many natural-log units above the data, and the resulting large $\exp(\cdot)$ pre-activations make Poisson NLL gradients numerically unstable during the first epochs of training. We therefore provide two LFADS configuration options for cold-starting the rate readout, used in the PhaseCodedMemory configurations and disabled (default) on all other canonical datasets: (i) `rate_bias_init` (bool), which at the start of fresh training sets the readout's per-neuron bias to $\log(\bar{y}_n)$ where $\bar{y}_n$ is the empirical mean spike count of neuron n on the training set; and (ii) `rate_weight_init_scale` (float, default 1.0), which multiplies the readout weight matrix at initialization. With the bias initialized at $\log(\bar{y}_n)$ and the weight matrix shrunk by a factor s, each neuron's predicted rate begins at its marginal mean with $\mathcal{O}(s)$ log-rate variability around it, which we found to substantially stabilize training on PhaseCodedMemory. The PCM canonical configurations (`PhaseCodedMemory_LFADS.yaml`, `PhaseCodedMemory_InjLFADS.yaml`) use `rate_bias_init: True` and `rate_weight_init_scale: 0.1`. Both flags are guarded to the Poisson exp link, take effect only at the start of fresh training, and have no effect when loading a saved model for inference or continued training.

E Analysis and Comparison Methods

E.1 Analysis Objects

Task-Trained Analysis We provide an object that abstracts the data handling away from the task-trained models or datamodules. This class `Analysis_TT` can be found at `ctd/comparison/analysis/tt/tt.py`, and contains helpful functions to obtain latent activity, compute fixed-points, and plot trials. Example usage of the `Analysis_TT` object can be found in `examples/compare_dd_tt_models.ipynb`. In addition, task-specific analysis objects can be found in the subfolder `ctd/comparison/analysis/tt/tasks`. These include functions to compute fixed-points during specific tasks and phases in MultiTask, among other useful features.

Data-Driven Analysis We also provide an analogous object for data-driven models called `Analysis_DD`, located at `ctd/comparison/analysis/dd/dd.py`. By passing the model class ("SAE" or "LFADS"), this model standardizes the functions used to obtain hidden activity, predicted rates, and other metrics from data-driven models of any class included in CtDToolkit. This object also allows for easy fixed-point computation and visualization.

Comparison The final object included in CtDToolkit is the `Comparison` object, located at `ctd/comparison/comparison.py`. Using `load_analysis()`, this object takes in `Analysis` objects (either task-trained or data-driven) and allows automated comparisons of metrics across models. Example usage of the `Comparison` object can be seen in `examples/compare_tt_dd_models.ipynb`.

E.2 Fixed Point Finding

As discussed in Section 2.4.3, fixed point structure and character can provide an informative qualitative picture of the neural dynamics, and possibly even their goal orientation. We seek to find the points in state space that minimize the magnitude of the kinetic energy of the dynamics, given by $\Delta h^2 \sim q$ in Equation 9. Because the estimated flow field is essentially never exactly zero to mathematical precision, we instead retain points whose speed falls below a chosen threshold; these slow regions act as *functional fixed points* over the timescales relevant to the behavior, rather than true fixed points where the flow vanishes exactly. The dynamics near such points are sufficiently slow to enable characterization of the dynamics through linearization. The location and stability of each of these fixed points gives us insight into the organization and nature of the features and motifs of the dynamics.

To perform fixed point finding, we use an extension of an existing fixed point finding package [16]. Using ADAM to perform stochastic gradient descent, we optimize the objective given in Equation 9.

$$\arg\min_{z \in Z} q(z,u) \ ; \ q(z,u) = \|f(z,u) - z\|_2 \tag{9}$$

Where $q(z,u)$ represents the magnitude of the state update at state z given inputs u. Only fixed points with q less than a threshold (depending on specific model) are retained for further analysis.

We characterize fixed point stability by the magnitude and sign of the eigenvalues of the Jacobian of the state transition function, evaluated at each fixed point. In a discrete dynamical system, if all eigenvalues have magnitudes within the unit circle, the fixed point is stable; otherwise, it is unstable.

E.3 Dynamical Similarity Analysis (DSA)

DSA is a method that combines data-driven techniques inspired from Koopman Operator Theory with Statistical Shape Analysis to identify, from data, whether or not two dynamical systems are equivalent. Two dynamical systems f and g are equivalent, or topologically conjugate, if there exists a homeomorphism ϕ that maps one system to another as follows:

$$f = \phi^{-1} \circ g \circ \phi \tag{10}$$

A natural similarity metric thus could be $\min_\phi \left| f(x) - \phi^{-1} \circ g(y) \circ \phi \right|_F$, averaged over samples from x and y recorded in a one-to-one fashion. Unfortunately, two problems exist with this direct approach. First, ϕ can be arbitrarily nonlinear and hence hard to optimize for, especially with insufficient data. Second, the two systems could be recorded independently, which may make it difficult to align trajectories across systems. DSA circumvents these two problems by mapping each system to an arbitrarily high-dimensional linear system. Conjugacies between linear systems are themselves linear maps, hence the similarity metric becomes Procrustes Analysis over Vector Fields (PAVF):

$$\mathrm{d}(A_x, A_y) = \min_C ||A_x - C A_y C^{-1}||_F \tag{11}$$

Here, A_x and A_y are the dynamics matrices of the linear systems fit to the high-dimensional embeddings of the two systems being compared, and C is a change-of-basis matrix. Typically in DSA, C is optimized over the orthogonal group, $O(n)$, rather than the more general group of invertible matrices $GL(n)$. This is because the metric over $O(n)$ is a proper metric, which has yet to be proven for $GL(n)$, and $O(n)$ is a compact group. Importantly, [17] showed that this method can be used to identify conjugacy even when ϕ was highly nonlinear. In this paper, we used the angular form of the distance, which scales from 0 (most similar) to $\pi/2$ (most dissimilar). In practice, however the full range is not always used, and so relative distances are typically emphasized over absolute.

According to Koopman Operator Theory [18], dynamical systems can be represented linearly by mapping them into a high- (oftentimes infinite-)dimensional space. Methods that approximate the Koopman operator include Dynamic Mode Decomposition, which is used in DSA [19]. Koopman Operator Theory suggests that operator eigenspectra can characterize conjugacy [20]; accordingly, other work has used the 2-Wasserstein distance between operator eigenvalues to compare dynamical systems [21], but we found that the PAVF better differentiated the models studied here.

E.3.1 Picking Hyperparameters

In practice, at least two main hyperparameters are required in delay-embedded DSA, which we chose to use here for its speed. First, the number of delays must be chosen to create the high-dimensional embedding–we swept a large range and did not find a significant change in the results. Second, low-rank regression is used to fit the dynamics matrices, hence the rank must be chosen. Here, we chose to reduce dimensionality of each of our systems first with PCA to the top 5 components, for which previous work suggests choosing a full rank dynamics matrix. However, if intuition is lacking, predictivity metrics can be used to help select hyperparameters.

E.3.2 Comparing DSA and fixed-point structure across model capacity

To illustrate how dynamical similarity and fixed-point structure depend on data-driven model capacity, we fit NODE- and GRU-based SAEs to the canonical 3BFF dataset across latent sizes $\hat{D} \in \{2, 3, 5, 8, 16, 32, 64\}$ (a single representative seed per size for the matrices and fixed-point panels) and compared each to the ground-truth 3BFF TT model using DSA and fixed-point finding (S8 Fig). Comparing the boxed DSA-to-TT row of the cross-comparison matrices (S8A Fig), replotted against latent size in S8B Fig, reveals qualitatively different trends for the two model families: NODE dynamics are most TT-like at intermediate capacity ($\hat{D} \approx$ 8–16) and become *more* dissimilar at high capacity, whereas GRU dissimilarity decreases monotonically and plateaus. This mirrors the fixed-point structure (S8C Fig): the $\hat{D} = 8$ NODE recovers a clean cube of stable and unstable fixed points, while the $\hat{D} = 64$ NODE develops a tangled, off-manifold structure, and both GRU models retain the expected cube. The DSA and fixed-point-finding hyperparameters used for this figure are given in Table S13, and the figure is produced by `examples/figures/supplementary/make_figure_dsa.py`.

E.4 Nonlinear Cycle Consistency

An alternative nonlinear cycle-consistency metric [11] has been published for datasets with arbitrary nonlinear observation models; below we give its definition and our implementation. Let $\hat{\mathbf{z}}_t \in \mathbb{R}^{\hat{d}}$ denote the inferred latent variables at time t, and let $\hat{\mathbf{n}}_t \in \mathbb{R}^N$ denote the corresponding predicted firing rates produced by the forward inferred observation model $\hat{\mathbf{g}} : \mathbb{R}^{\hat{d}} \to \mathbb{R}^N$ (i.e., $\hat{\mathbf{n}}_t = \hat{\mathbf{g}}(\hat{\mathbf{z}}_t)$). *Notation: noise-perturbed quantities are written with a tilde and a superscript* (σ) *(e.g.,* $\tilde{\mathbf{n}}_t^{(\sigma)}$*), while baseline inferred quantities retain hats (e.g.,* $\hat{\mathbf{z}}_t$*).*

1. Learned generalized inverse We first train a nonlinear mapping $g_\phi^\dagger : \mathbb{R}^N \to \mathbb{R}^{\hat{d}}$ (an MLP parameterized by ϕ) to approximate an inverse of the observation model $\hat{\mathbf{g}}$ from firing rates to latent variables. The parameters ϕ are optimized to minimize MSE on a training set:

$$\phi^\star = \arg\min_\phi \mathbb{E}_{t \in \mathcal{D}_{\text{train}}} \Big[\| g_\phi^\dagger(\hat{\mathbf{n}}_t) - \hat{\mathbf{z}}_t \|_2^2 \Big].$$

Here $g_\phi^\dagger$ is a *learned* generalized inverse of $\hat{\mathbf{g}}$; it need not coincide with a true inverse and is trained independently from $\hat{\mathbf{g}}$.

2. Noise injection. After training, for each validation sample, we inject independent Gaussian noise into the predicted rates, scaled by each neuron's empirical variability:

$$\tilde{\mathbf{n}}_t^{(\sigma)} = \hat{\mathbf{n}}_t + \boldsymbol{\epsilon}, \qquad \boldsymbol{\epsilon} \sim \mathcal{N}\big(0, \operatorname{diag}(\sigma^2 \operatorname{std}_1^2, \ldots, \sigma^2 \operatorname{std}_N^2)\big),$$

where std_i is the standard deviation of neuron i's log-rates in the training data. This ensures perturbations are proportional to each neuron's natural firing-rate scale.

3. Latent reconstruction. We apply the trained generalized inverse to the perturbed rates to obtain reconstructed latents:

$$\tilde{\mathbf{z}}_t^{(\sigma)} = g_{\phi^\star}^\dagger(\tilde{\mathbf{n}}_t^{(\sigma)}).$$

4. Quantification of degradation. For each noise level σ, we evaluate the coefficient of determination (R^2) between reconstructed and original inferred latents on the validation set:

$$R^2(\sigma) \;=\; 1 - \frac{\sum_t \|\hat{\mathbf{z}}_t - \tilde{\mathbf{z}}_t^{(\sigma)}\|_2^2}{\sum_t \|\hat{\mathbf{z}}_t - \bar{\hat{\mathbf{z}}}\|_2^2}, \qquad \bar{\hat{\mathbf{z}}} = \tfrac{1}{T}\sum_t \hat{\mathbf{z}}_t.$$

The `nl_cycle_con` curve is $R^2(\sigma)$ as a function of the noise magnitude σ. Intuitively, if the forward observation model $\hat{\mathbf{g}} : \hat{\mathbf{z}} \mapsto \hat{\mathbf{n}}$ is non-injective, small perturbations in rate space ($\hat{\mathbf{n}}$-space) can yield disproportionately large deviations in reconstructed latents, causing rapid degradation of $R^2(\sigma)$.

We show in S9A Fig example `nl_cycle_con` curves for $\sigma \in [0, 0.01, 0.05, 0.1, 0.25, 0.5]$, and the relationship between linear and nonlinear cycle consistency in S9B Fig (nl_cycle_con evaluated at $\sigma = 0.05$).

MLP hyperparameters and overfitting controls. The learned generalized inverse $g_\phi^\dagger$ is a multilayer perceptron with two hidden layers of 64 units each and ReLU activations, mapping inferred firing rates to inferred latents. It is trained to minimize the MSE between predicted and inferred latents using the Adam optimizer (learning rate 10^{-3}, weight decay 10^{-4}) for up to 1000 epochs in full-batch mode. To guard against overfitting of the inverse mapping, the inferred latents and rates are split into training and validation sets and the metric employs early stopping: the validation MSE is monitored each epoch, training halts when the validation loss fails to improve by at least 10^{-5} for 50 consecutive epochs, and the best-validation parameters are then restored. Because a brittle, overfit inverse could itself amplify small rate perturbations and produce rapid degradation of $R^2(\sigma)$ that could be mistaken for a non-injective observation model, we recommend confirming that the training and validation losses track closely (i.e., a small generalization gap) before interpreting the `nl_cycle_con` curve. For the results shown here, the inverse MLP was trained with early stopping on validation loss as described above, and we observed no evidence of overfitting: the training and validation loss trajectories tracked closely across all latent sizes (S9C Fig). All of these hyperparameters are exposed in the metric configuration and can be adjusted by the user.

E.5 Distribution Matching (Wasserstein Distance)

The metric takes as input training and validation trajectories from the reference data and the corresponding model-predicted activity. Because raw spikes can produce noisy empirical distributions, trajectories may first be temporally binned and/or smoothed. Optionally, we fit principal components analysis (PCA) using only the training observations and project both the reference activity and model predictions into this same PCA space; when PCA is disabled, the comparison is carried out directly in the original observation channels. When used, this projection ensures that the comparison is performed in a shared observation-derived coordinate system rather than in the potentially arbitrary latent coordinates of the model.

Optionally, we construct a delay embedding of the PCA-projected trajectories. For a trajectory $\mathbf{x}_{1:T}$, with delay lag τ and number of delays K, the delay-embedded state at time t is given by

$$\tilde{\mathbf{x}}_t = [\mathbf{x}_t, \mathbf{x}_{t-\tau}, \mathbf{x}_{t-2\tau}, \ldots, \mathbf{x}_{t-K\tau}]\,. \tag{12}$$

This embedding augments each sample with recent temporal history, allowing the metric to compare distributions over local trajectory segments rather than instantaneous activity alone. After optional PCA projection and delay embedding, the validation trajectories are flattened across trials and time to produce two point clouds: $\tilde{\mathbf{X}}^{\text{val}}$ for the reference activity and $\tilde{\mathbf{Y}}^{\text{val}}$ for the model-predicted activity. For computational efficiency, each point cloud may be randomly subsampled to a fixed maximum number of points before the distributional comparison.

Following the marginal CDF-based approach, we compute a one-dimensional Wasserstein-1 distance independently for each dimension of the flattened point clouds. For dimension d, this distance is

$$W_1^{(d)} = \int_{-\infty}^{\infty} \left| F_{\tilde{\mathbf{X}}}^{(d)}(z) - F_{\tilde{\mathbf{Y}}}^{(d)}(z) \right| dz, \tag{13}$$

where $F_{\tilde{\mathbf{X}}}^{(d)}$ and $F_{\tilde{\mathbf{Y}}}^{(d)}$ are the empirical cumulative distribution functions of the reference and model-predicted samples along dimension d. Equivalently, in one dimension this distance can be computed by comparing the empirical quantile functions of the two distributions.

To make the metric comparable across dimensions with different scales, we normalize each dimension by the range of the reference validation samples:

$$\hat{W}_1^{(d)} = \frac{2W_1^{(d)}}{\max_i \tilde{X}_{i,d}^{\text{val}} - \min_i \tilde{X}_{i,d}^{\text{val}} + \epsilon}. \tag{14}$$

Here $\epsilon = 10^{-12}$ is a small constant that prevents division by zero. The final metric is the average normalized Wasserstein distance across the D dimensions of the comparison space (i.e., the flattened, PCA-projected, and delay-embedded point clouds):

$$\mathrm{W}_{\mathrm{CDF}} = \frac{1}{D}\sum_{d=1}^{D} \hat{W}_1^{(d)}. \tag{15}$$

Lower values indicate that the model-predicted activity more closely matches the empirical distribution of the reference validation data in the observation-derived comparison space.

Importantly, this is not a joint multivariate optimal transport distance. Instead, it is an average of one-dimensional Wasserstein distances computed independently across dimensions. This makes the metric computationally efficient and robust in moderate-dimensional spaces, but it also means that correlations between dimensions are not directly assessed except insofar as they are reflected in the PCA and delay-embedding coordinates. Additionally, because samples are flattened across trials and time before the distributional comparison, this metric is order-agnostic: it compares the distribution of visited states or delay-embedded trajectory segments, but does not directly compare trial-by-trial temporal alignment or the underlying flow field.

E.6 Lyapunov Spectrum

Jacobian Computation For each time step t and sampled trajectory, we compute the Jacobian matrices:

$$J_z^{(t)} = \left.\frac{\partial f}{\partial \mathbf{z}}\right|_{(\mathbf{z}_t,\mathbf{u}_t)} \in \mathbb{R}^{D\times D} \tag{16}$$

$$\tag{17}$$

where D is the dimensionality of the state space. These Jacobians are computed using automatic differentiation over multiple sampled trajectories.

Lyapunov Exponent Estimation The Lyapunov exponents are estimated using the QR decomposition algorithm (adapted from [22]). We initialize an orthonormal matrix $Q_0 = I_D$ and iteratively apply the following steps:

1. Compute the matrix product: $M_t = J_z^{(t)} Q_{t-1}$
2. QR decomposition: $M_t = Q_t R_t$ where Q_t is orthonormal and R_t is upper triangular
3. Ensure positive diagonal elements: $R_t \leftarrow \mathrm{sign}(\mathrm{diag}(R_t)) \cdot R_t$, $Q_t \leftarrow Q_t \cdot \mathrm{sign}(\mathrm{diag}(R_t))$
4. Accumulate logarithmic growth rates: $\lambda_i^{(t)} = \lambda_i^{(t-1)} + \ln(R_{ii}^{(t)})$

After T time steps, the Lyapunov exponents are estimated as:

$$\lambda_i = \frac{1}{T\cdot\Delta t}\sum_{t=1}^{T} \ln(R_{ii}^{(t)})$$

where Δt is the discrete time step size.

We take the maximal Lyapunov exponent $\lambda_{\max} = \max_i \lambda_i$ as our primary stability metric. This quantity governs the overall stability of the dynamical system:

- $\lambda_{\max} < 0$: System is asymptotically stable (perturbations decay exponentially)

- $\lambda_{\max} = 0$: System is marginally stable
- $\lambda_{\max} > 0$: System exhibits chaotic behavior (perturbations grow exponentially)

We report the maximal Lyapunov exponent as our primary stability metric because it is the component most directly estimable from data, and therefore the one most readily compared across TT and DD models. The maximal exponent alone, however, does not fully characterize stability: the complete Lyapunov spectrum is required to distinguish, for example, a chaotic *attracting* set (positive maximal exponent but negative sum of exponents) from a divergent one. CtDToolkit therefore provides a `compute_lyapunov_spectrum` routine (and a corresponding `Analysis_TT.compute_lyapunov_spectrum` method) that returns the full ordered set of exponents $\{\lambda_1 \geq \cdots \geq \lambda_D\}$ together with summary statistics, including their sum $\sum_i \lambda_i$ (the mean rate of phase-space volume contraction) and the Kaplan–Yorke (Lyapunov) dimension. We validated this estimator on the canonical Lorenz and Rössler systems, recovering spectra that closely match their literature references (S10 Fig). For the Lorenz attractor, the estimated spectrum $[+0.91, -0.01, -14.56]$ reproduces the reference $[+0.906, 0, -14.572]$, with $\lambda_{\max} > 0$ (chaos) and $\sum_i \lambda_i \approx -13.7$ (an attracting set; Kaplan–Yorke dimension ≈ 2.06). For the Rössler attractor, the estimated spectrum $[+0.07, +0.01, -5.48]$ likewise matches the reference $[+0.071, 0, -5.394]$. The worked example is provided at `examples/figures/supplementary/lyapunov_spectrum_lorenz.py`.

E.7 Example Performance of DD Models on CtDToolkit Datasets

To provide a demonstration of the performance metrics applied to each dataset, we replicated the experiment shown in Fig 5 for 3BFF/NBFF, MultiTask, RandomTarget, PhaseCodedMemory, and ChaoticDelayedMatching. For each dataset, we trained DD models across a sweep of latent dimensionalities, using five random seeds per latent size (NODE-based SAE models for 3BFF/NBFF, MultiTask, RandomTarget, and ChaoticDelayedMatching; LFADS models for PhaseCodedMemory). The sweeps contain seven latent sizes for 3BFF/NBFF, six latent sizes for MultiTask, and eight latent sizes each for RandomTarget, PhaseCodedMemory, and ChaoticDelayedMatching. The figures below show the completed sweeps for all five datasets. After training was complete, we used Analysis and Comparison objects to evaluate the performance of all trained models on 6 metrics: 1) Rate R^2, 2) State R^2, 3) co-BPS, 4) Cycle-Consistency, 5) Wasserstein distance, and 6) estimated maximal Lyapunov exponent. The model and training hyperparameters for these sweeps are compiled in Table S14 (NODE-based SAE sweeps for 3BFF/NBFF, MultiTask, RandomTarget, and ChaoticDelayedMatching) and Table S15 (LFADS sweep for PhaseCodedMemory).

E.8 DD Model Validation on PhaseCodedMemory

To complement the canonical metric sweeps above, we examine an example data-driven (DD) model fit on the cyclic PhaseCodedMemory dataset. This example illustrates how CtDToolkit metrics and visualizations can be used to assess whether a DD model has recovered the correct dynamical strategy of the underlying TT system.

PhaseCodedMemory: LFADS recovers a rate-coding rather than phase-coding solution. We trained LFADS models across a range of latent dimensionalities (8D, 16D, and 128D) on the canonical PhaseCodedMemory dataset (S4 Fig) and compared the inferred firing rates and latent activity against the ground-truth TT model (S16 Fig). Larger models achieved better reconstruction of the simulated firing rates (Rate R^2 increasing from 0.68 at 8D to 0.77 at 128D), but at the cost of a degrading State R^2 (decreasing from 0.97 to 0.92), indicating that their inferred latents drift further from the ground-truth TT latent structure even as they fit the observed rates more closely. Critically, at every latent size the inferred dynamics failed to capture the qualitative phase-coding strategy of the TT model: whereas the ground-truth Stim A and Stim B trajectories form overlapping, rotated rings in both the rate-space and latent-space PCs, the DD models inferred latents in which the two stimulus conditions are largely *separated*, consistent with a rate-coding rather than phase-coding strategy. This demonstrates how DD-inferred dynamics, when interpreted without reference to ground truth, can lead to qualitatively incorrect conclusions about the underlying neural computation, even for models that reconstruct the observed activity well. We additionally swept LFADS models across latent dimensionalities on this dataset and compiled the full suite of CtDToolkit metrics as a function of latent size (S14 Fig).

F Utilizing the Task-Training Pipeline

We used PyTorch Lightning to modularize the code-base to allow for easy additions and extensions from users. The CtDToolkit pipeline defines five objects designed to simplify the experience for the user. These objects are:

1. TaskEnvironment: The task environment (described in Section F.1, schematic in S17 Fig), contains the task logic for a given task. TaskEnvironment inherits from Gym.Environment [23], meaning that new TaskEnvironments must implement the `step()` and `reset()` functions. Additionally, it must implement the `generate_dataset()` function, which generates a dictionary of inputs and desired outputs for the task-trained model.

2. TT model: Inherits from nn.Module [24]. This object (schematic in S17 Fig) defines the model that will be trained to perform the task. This object must implement the `forward()` method, which takes in the input and the hidden state at time t to output the hidden state at time $t + 1$. Additionally, this object must implement the `init_model()` method and (optionally), the `init_hidden()` method.

3. TaskWrapper: Inherits from LightningModule [25]. This object takes in the TaskEnvironment and the TT model and handles the training and validation loops. This object should not need to be modified by the typical user.

4. TTDatamodule: Inherits from LightningDataModule [25]. This object handles the data generation from the TaskEnvironment and batching during training and validation. This object should not need to be modified by the typical user.

5. Simulator: This object handles the generation of synthetic neural activity from the trained TT model. The `__init__()` method of this object takes in two dictionaries, `noise_dict` and `embed_dict` (the simulator configuration name for observation-model settings), which provide settings for the noise model used to sample spiking events from the simulated firing rates and the nonlinear rectification/observation model of the task-trained model's hidden unit activity, respectively. The Simulator object should not need to be modified by the typical user. We provide more detail on the Simulator in Section C.

F.1 Task Environment Requirements

Our task environments adhere to a general format that is modular and easily extensible, allowing researchers to add new tasks to CtDToolkit without major code restructuring. TaskEnvironments are subclasses of Gymnasium Gym environments [23]. In total, new task environments must meet the following requirements:

Required Variables:

- `dataset_name`: *string*. Used as an identifier when saving and loading datasets in the task-training and data-training pipelines.

Input/Outputs: These variables define the input/output spaces of the task. All are built using Gymnasium Space objects.

- `action_space`: *Gymnasium Space*. Defines the allowable range of actions to be taken in the environment. `3BFF`, `PhaseCodedMemory`, `MultiTask`, and `RandomTarget` have 3,1, 3, and 6, respectively.

- `observation_space`: *Gymnasium Space*. Defines the allowable range of "sensory" inputs that the environment provides. `3BFF`, `PhaseCodedMemory`, `MultiTask`, and `RandomTarget` have 3,3, 20, and 14, respectively.

- `context_inputs`: *Gymnasium Space*. Defines the allowable range of "context" inputs to the model. Only `RandomTarget` currently has non-zero context inputs (3).

Additional Variables:

- `noise`: *float*. Describes the standard deviation of Gaussian noise to be added to the inputs in the pre-generated dataset.

- `n_timesteps`: *int*. Provides the trial length for the TaskEnvironment (in bins).
- `coupled_env`: *bool*. Indicates whether the actions need to be provided to the environment to generate the next step of sensory inputs (i.e., if the controller and environment are coupled). Only `True` for `RandomTarget` for canonical datasets.
- `loss_func`: A CtDToolkit class that takes in a `loss_dict` and computes the loss for the environment. See `/ctd/task_modeling/task_env/loss_func.py` for implementations of each `TaskEnvironment` loss.

Required Methods:

- `step(self, action)`:
  Inherited from Gym. Should implement the state update for a given `TaskEnvironment`.
- `reset(self)`: Inherited from Gym. Should reset required variables to restart trial (e.g., set initial states, etc.).
- `generate_dataset(self, n_samples)`:
  Defines how many trials should be generated from the dataset; for `MultiTask`, `n_samples` defines the number of trials to be generated for each sub-task. This method should return a dictionary containing the fields given in Section B.1.

We provide a template TaskEnvironment in `/ctd/task_modeling/task_env/task_env.py`

F.2 How to Submit a CtDToolkit Dataset

1. **Fork the repository.** Create a personal fork of the `CtDToolkit` GitHub repository.
2. **Implement a new task environment.** In your fork, add a new task environment that conforms to the Toolkit's task-environment API (e.g., initialization, reset, step, observation, reward functions), described above.
3. **Train a task-trained model.** Train a model to perform the task within your new environment. Save both (i) the trained model and (ii) a simulator object capable of generating simulated spiking activity. To use the Analysis and Comparison object methods, the TT model must adhere to the template provided in `/ctd/task_modeling/model/tt_template.py`
4. **Validate model performance.** Demonstrate that the trained model can *adequately perform the task* (e.g., achieving appropriate behavioral performance or reward thresholds) and produces realistic neural activity (e.g., firing-rate statistics or spiking patterns). Summarize these results for review.
5. **Register the dataset.** Modify the `gen_datasets.py` script to add your dataset, linking it to the saved model and simulator object. This ensures your dataset is integrated into the Toolkit's dataset generation pipeline.
6. **(Recommended) Add documentation.** Provide a short "dataset card" including a description of the task, training details, validation results, and an example usage snippet.
7. **Submit a pull request.** Open a pull request (PR) to the main branch of `CtDToolkit`, briefly describing the task, what makes the dataset novel or useful, and including your validation summary.
8. **Review and merge.** The maintainers will review the PR to ensure correctness, novelty, and scientific utility. Once approved, the PR is merged, and the dataset becomes available as part of the official `CtDToolkit` test-set collection.

G Compute Resources

We used an internal computing cluster with a total of 30 Nvidia GeForce RTX 2080 Ti GPUs for model training. The task-training pipeline took 0.5, 8, and 4 hours to train for the 3BFF, MultiTask, and RandomTarget models, respectively. The RandomTarget task does not parallelize, due to its serial nature, so we did not use GPUs to train that model. For the data-driven models, both the SAE and LFADS models each took less than 1 hour to train; we trained a total of 95 data-driven models across all figures. With 2 models training on each GPU, all experiments took <150 GPU-hours. FP finding was fast, requiring 1 minute for each model.

H Open-source Packages Used

- `torch` [24] (BSD license)
- `pytorch-lightning` [25] (Apache 2.0 license)
- `ray.tune` [9] (Apache 2.0 license)
- `lfads-torch` [14] (Apache 2.0 license)
- `hydra` [8] (MIT license)

References

[1] Sussillo D, Barak O. Opening the black box: Low-dimensional dynamics in high-dimensional recurrent neural networks. Neural Computation. 2013;25(3):626-49. Available from: http://www.mitpressjournals.org/doi/10.1162/NECO_a_00409. doi:10.1162/NECO_a_00409.

[2] Yang GR, Joglekar MR, Song HF, Newsome WT, Wang XJ. Task representations in neural networks trained to perform many cognitive tasks. Nature Neuroscience. 2019;22(2):297-306. Available from: http://www.nature.com/articles/s41593-018-0310-2. doi:10.1038/s41593-018-0310-2.

[3] Driscoll L, Shenoy K, Sussillo D. Flexible multitask computation in recurrent networks utilizes shared dynamical motifs. bioRxiv;. Pages: 2022.08.15.503870 Section: New Results. Available from: https://www.biorxiv.org/content/10.1101/2022.08.15.503870v1. doi:10.1101/2022.08.15.503870.

[4] Codol O, Michaels JA, Kashefi M, Pruszynski JA, Gribble PL. MotorNet: a Python toolbox for controlling differentiable biomechanical effectors with artificial neural networks. eLife. 2024;12. Publisher: eLife Sciences Publications Limited. Available from: https://elifesciences.org/reviewed-preprints/88591. doi:10.7554/eLife.88591.2.

[5] Pals M, Macke JH, Barak O. Trained recurrent neural networks develop phase-locked limit cycles in a working memory task. PLoS Comput Biol. 2024 Feb;20(2):e1011852.

[6] Miconi T. Biologically plausible learning in recurrent neural networks reproduces neural dynamics observed during cognitive tasks. eLife. 2017;6:e20899. doi:10.7554/eLife.20899.

[7] Sussillo D, Abbott LF. Generating coherent patterns of activity from chaotic neural networks. Neuron. 2009;63(4):544-57.

[8] Yadan O. Hydra - A framework for elegantly configuring complex applications; 2019. Github. Available from: https://github.com/facebookresearch/hydra.

[9] Liaw R, Liang E, Nishihara R, Moritz P, Gonzalez JE, Stoica I. Tune: A Research Platform for Distributed Model Selection and Training. arXiv;. Available from: http://arxiv.org/abs/1807.05118. arXiv:1807.05118 [cs, stat].

[10] Chen RTQ, Rubanova Y, Bettencourt J, Duvenaud D. Neural Ordinary Differential Equations. In: Advances in Neural Information Processing Systems. vol. 109; 2018. p. 31-60. Available from: https://arxiv.org/abs/1806.07366v5. arXiv:1806.07366.

[11] Versteeg C, Sedler AR, McCart JD, Pandarinath C. Expressive dynamics models with nonlinear injective readouts enable reliable recovery of latent features from neural activity. In: Sanborn S, Shewmake C, Azeglio S, Miolane N, editors. Proceedings of the 2nd NeurIPS Workshop on Symmetry and Geometry in Neural Representations. vol. 228 of Proceedings of Machine Learning Research. PMLR; 2024. p. 255-78. Available from: https://proceedings.mlr.press/v228/versteeg24a.html.

[12] Sedler AR, Versteeg C, Pandarinath C. Expressive architectures enhance interpretability of dynamics-based neural population models. arXiv; 2022. Available from: https://arxiv.org/abs/2212.03771v1. doi:10.48550/arXiv.2212.03771.

[13] Pandarinath C, O'Shea DJ, Collins J, Jozefowicz R, Stavisky SD, Kao JC, et al. Inferring single-trial neural population dynamics using sequential auto-encoders. Nat Methods. 2018;15(10):805-15.

[14] Sedler AR, Pandarinath C. lfads-torch: A modular and extensible implementation of latent factor analysis via dynamical systems; 2023. arXiv:2309.01230.

[15] Kim TD, Luo TZ, Pillow JW, Brody CD. Inferring latent dynamics underlying neural population activity via neural differential equations. In: International Conference on Machine Learning. PMLR; 2021. p. 5551-61.

[16] Golub MD, Sussillo D. FixedPointFinder: A Tensorflow toolbox for identifying and characterizing fixed points in recurrent neural networks. Journal of Open Source Software. 2018;3(31):1003. Available from: https://joss.theoj.org/papers/10.21105/joss.01003. doi:10.21105/joss.01003.

[17] Ostrow M, Eisen AJ, Kozachkov L, Fiete IR. Beyond Geometry: Comparing the Temporal Structure of Computation in Neural Circuits with Dynamical Similarity Analysis. In: Thirty-seventh Conference on Neural Information Processing Systems; 2023. Available from: https://openreview.net/forum?id=7blSUMwe7R.

[18] Mezić I. Spectral properties of dynamical systems, model reduction and decompositions. Nonlinear Dynamics. 2005;41:309-25.

[19] Brunton SL, Brunton BW, Proctor JL, Kaiser E, Kutz JN. Chaos as an Intermittently Forced Linear System. Nature Communications. 2017;8(1):19.

[20] Budišić M, Mohr R, Mezić I. Applied Koopmanism. Chaos: An Interdisciplinary Journal of Nonlinear Science. 2012 Dec;22(4):047510.

[21] Redman WT, Bello-Rivas JM, Fonoberova M, Mohr R, Kevrekidis IG, Mezić I. Identifying Equivalent Training Dynamics; 2024. arXiv:2302.09160.

[22] Eisen AJ, Ostrow M, Chandra S, Kozachkov L, Miller EK, Fiete IR. Characterizing control between interacting subsystems with deep Jacobian estimation; 2025. Available from: https://arxiv.org/abs/2507.01946. arXiv:2507.01946.

[23] Towers M, Terry JK, Kwiatkowski A, Balis JU, Cola Gd, Deleu T, et al.. Gymnasium. Zenodo; 2023. Available from: https://zenodo.org/record/8127025. doi:10.5281/zenodo.8127026.

[24] Paszke A, Gross S, Massa F, Lerer A, Bradbury J, Chanan G, et al. PyTorch: An Imperative Style, High-Performance Deep Learning Library. arXiv; 2019. arXiv:1912.01703. Type: article. Available from: http://arxiv.org/abs/1912.01703. arXiv:1912.01703 [cs, stat]. doi:10.48550/arXiv.1912.01703.

[25] Falcon W, The PyTorch Lightning team. PyTorch Lightning; 2019. Available from: https://github.com/Lightning-AI/lightning. doi:10.5281/zenodo.3828935.
