# Supplementary material for: Computation-through-Dynamics Toolkit: Simulated datasets and quality metrics for dynamical models of neural activity
Source: PLoS Comput Biol. 2026 Oct 15;22(10):e1014755. doi: 10.1371/journal.pcbi.1014755 (PMC13712701; doi:10.1371/journal.pcbi.1014755)
Supplement: Supplementary file 2 [file pcbi.1014755.s021.pdf]

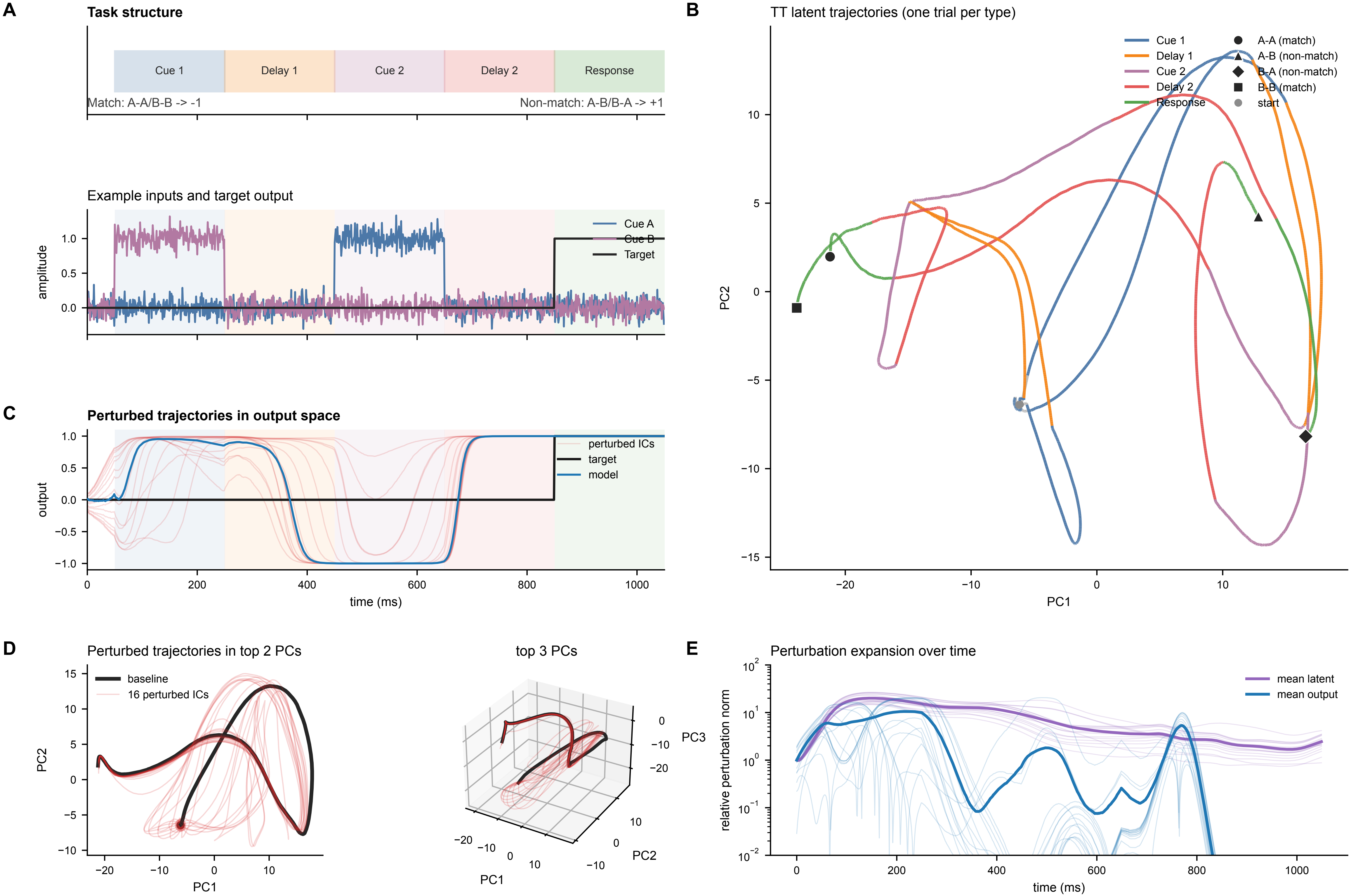

A
Task structure
Cue 1
Delay 1
Cue 2
Delay 2
Response
Match: A-A/B-B -> -1
Non-match: A-B/B-A -> +1
Example inputs and target output
amplitude
Cue A
Cue B
Target
B
TT latent trajectories (one trial per type)
Cue 1
Delay 1
Cue 2
Delay 2
Response
A-A (match)
A-B (non-match)
B-A (non-match)
B-B (match)
start
PC1
PC2
C
Perturbed trajectories in output space
perturbed ICs
target
model
output
time (ms)
D
Perturbed trajectories in top 2 PCs
baseline
16 perturbed ICs
PC1
PC2
top 3 PCs
PC1
PC2
PC3
E
Perturbation expansion over time
mean latent
mean output
relative perturbation norm
time (ms)
