# Supplementary material for: Computation-through-Dynamics Toolkit: Simulated datasets and quality metrics for dynamical models of neural activity
Source: PLoS Comput Biol. 2026 Oct 15;22(10):e1014755. doi: 10.1371/journal.pcbi.1014755 (PMC13712701; doi:10.1371/journal.pcbi.1014755)
Supplement: Supplementary file 3 [file pcbi.1014755.s024.pdf]

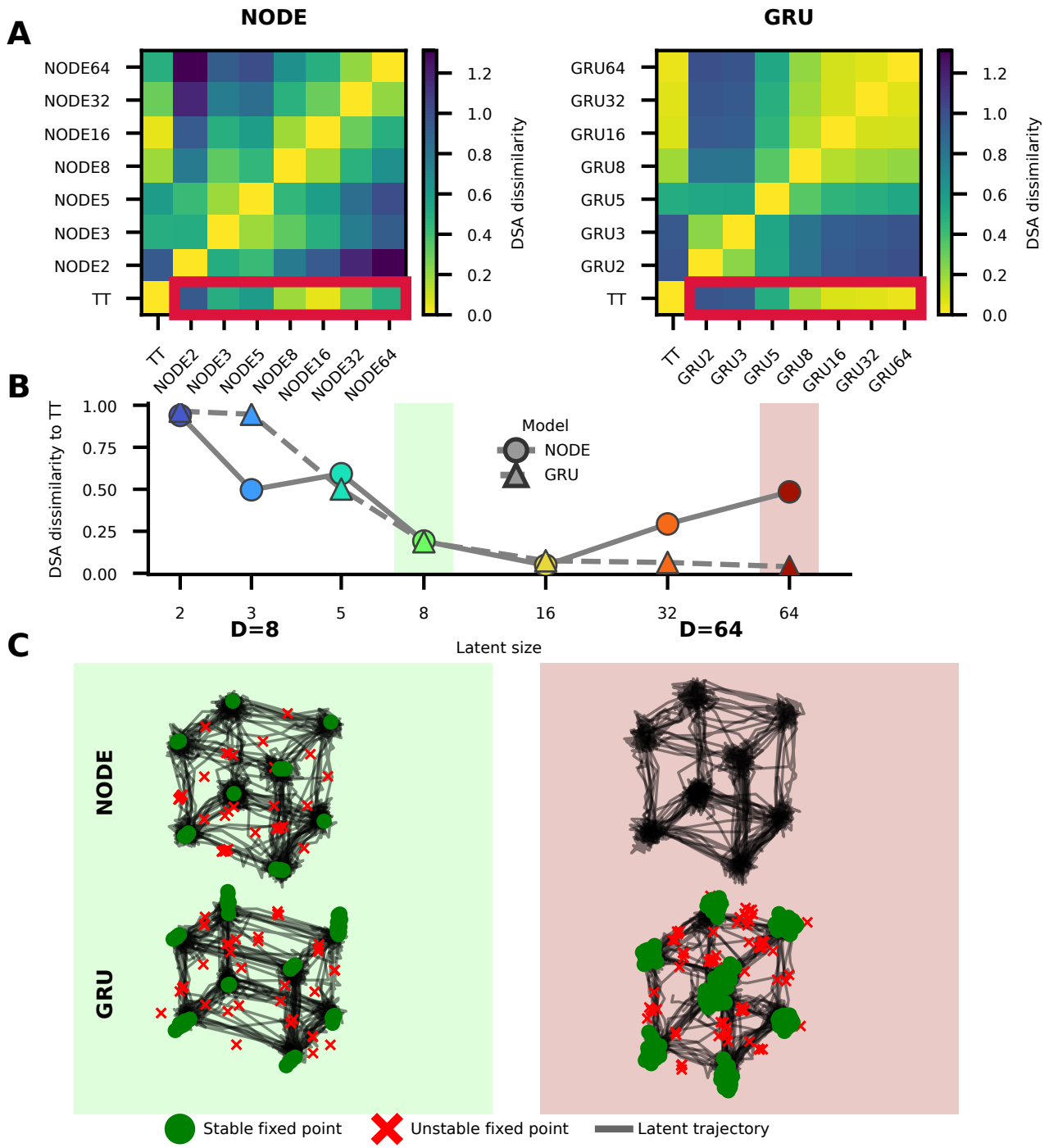
A
NODE
GRU
NODE64
NODE32
NODE16
NODE8
NODE5
NODE3
NODE2
TT
GRU64
GRU32
GRU16
GRU8
GRU5
GRU3
GRU2
DSA dissimilarity
0.0
0.2
0.4
0.6
0.8
1.0
1.2
B
DSA dissimilarity to TT
Model
NODE
GRU
1.00
0.75
0.50
0.25
0.00
2
3
5
8
16
32
64
Latent size
C
D=8
D=64
NODE
GRU
Stable fixed point
Unstable fixed point
Latent trajectory
