# Supplementary material for: Computation-through-Dynamics Toolkit: Simulated datasets and quality metrics for dynamical models of neural activity
Source: PLoS Comput Biol. 2026 Oct 15;22(10):e1014755. doi: 10.1371/journal.pcbi.1014755 (PMC13712701; doi:10.1371/journal.pcbi.1014755)
Supplement: Supplementary file 4 [file pcbi.1014755.s026.pdf]

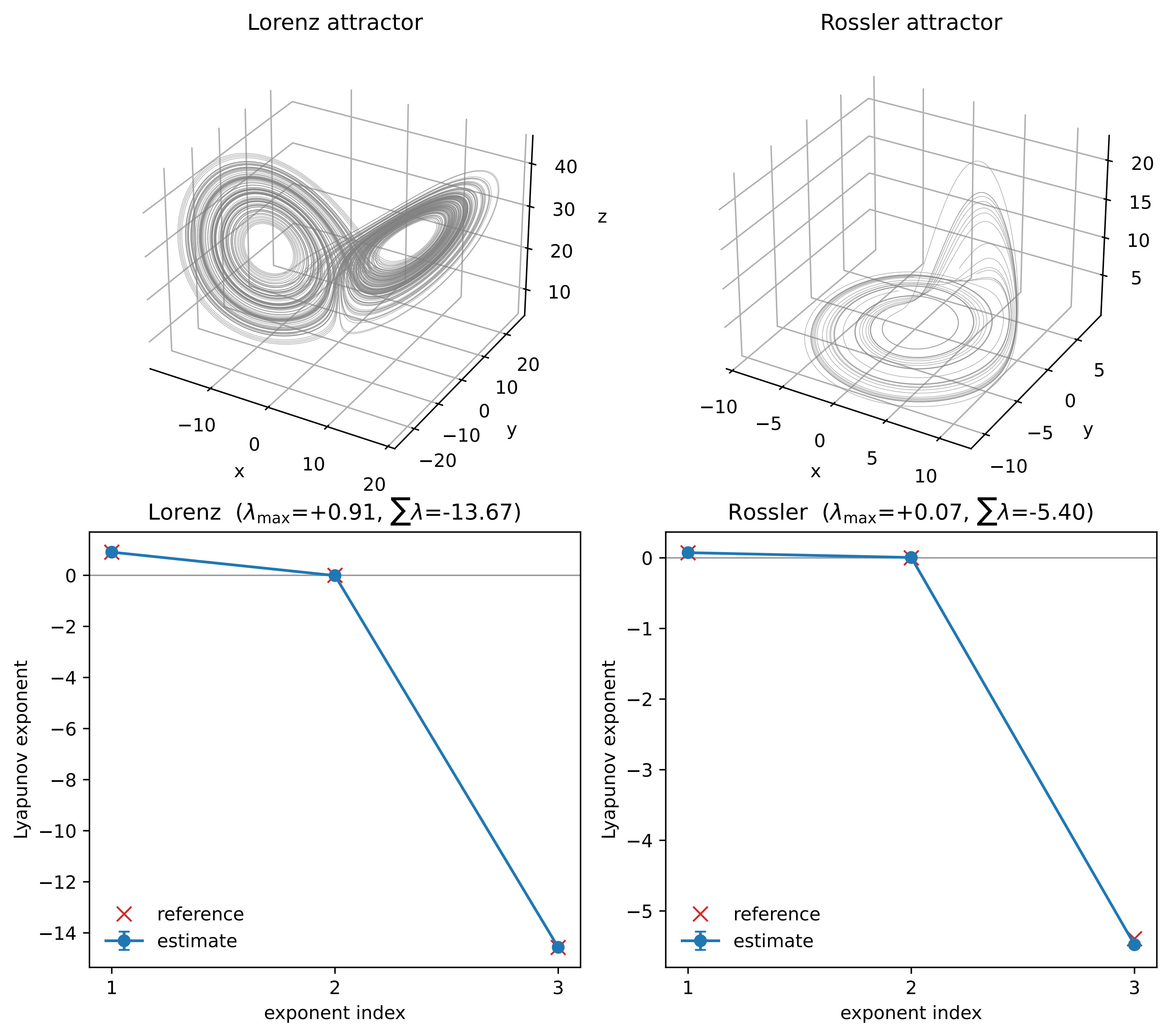

Lorenz attractor
Rossler attractor
x
y
z
Lorenz (λ_{max}=+0.91, $\sum\lambda$=-13.67)
Rossler (λ_{max}=+0.07, $\sum\lambda$=-5.40)
Lyapunov exponent
exponent index
reference
estimate
