# Supplementary material for: Computation-through-Dynamics Toolkit: Simulated datasets and quality metrics for dynamical models of neural activity
Source: PLoS Comput Biol. 2026 Oct 15;22(10):e1014755. doi: 10.1371/journal.pcbi.1014755 (PMC13712701; doi:10.1371/journal.pcbi.1014755)
Supplement: Supplementary file 5 [file pcbi.1014755.s035.pdf]

Response to Reviewers

Computation-through-Dynamics Toolkit (PLOS resubmission)

May 31, 2026

We thank the editor and reviewers for their careful reading and constructive feedback. Below we reproduce each comment *(in shaded italics)* followed by our response. Manuscript references (e.g. "Fig. 5", "Appendix D") point to the revised manuscript and are generated automatically from its labels. Changes in the manuscript are summarized after each response.

Editor

Thank you for submitting your manuscript to PLOS Computational Biology. After careful consideration, we feel that it has merit but does not fully meet PLOS Computational Biology's publication criteria as it currently stands. Therefore, we invite you to submit a revised version of the manuscript that addresses the points raised during the review process.
Both the reviewers and I appreciate the considerable revisions that improve the clarity of your manuscript, and we are also all in agreement that the aim of your project is timely and important. However, the are some remaining issues that need to be taken care of to increase the impact of your work.

Response: We thank the editor and both reviewers for their thoughtful and constructive engagement, and for the encouraging assessment that the work is timely and important. We have revised the manuscript to address each of the remaining issues raised during review. The most substantial changes are summarized below; point-by-point responses follow in the reviewer sections.

New datasets and tooling:

- Added a new chaotic benchmark dataset, ChaoticDelayedMatching, providing an example with near-chaotic dynamics (Reviewer 1, point 1).
- Added full Lyapunov-spectrum tooling, validated against the canonical Lorenz and Rössler attractors (Reviewer 1, p. 12/13).

Metrics and methodology:

- Replaced MMD with the Wasserstein distance as our primary geometry-based metric, consistent with established practice in the dynamical-systems literature (Reviewer 1, point 2).
- Clarified the scope and limitations of the injectivity argument, emphasizing that it is one modeling constraint among several rather than a guarantee of correct inference (Reviewer 1, point 3; Reviewer 2, point 3).

Framing, terminology, and corrections:

- Expanded the discussion to connect CtDToolkit to the broader dynamical-systems reconstruction literature (Reviewer 1, minor points).
- Sharpened terminology throughout (e.g. "observation model" for the latent-to-rate mapping, "biologically motivated" models, precise use of "dynamics").
- Corrected the technical inconsistencies identified in the figures and regenerated the affected panels from a common source (Reviewer 2, point 5).

Presentation and readability:

- Performed a thorough clarity and concision pass over the main text (abstract through discussion), tightening wording and removing redundancy, and reorganized the metric results and the discussion into clearly labeled subsections for easier navigation.
- Standardized mathematical notation throughout—e.g. $\mathbf{g}/\hat{\mathbf{g}}$ for the true/inferred observation model, $\mathbf{x}/\hat{\mathbf{x}}$ for target/predicted outputs, and $\mathbf{u}$ for inputs—and expanded symbol definitions throughout the appendix equations.
- Gave the appendix task descriptions a uniform structure (general description, environment parameters, trial structure, loss function, and task-trained model) and improved the appendix layout so that each task, metric, and section starts cleanly and figures appear alongside the text that describes them.

We believe these changes meaningfully strengthen the manuscript.

Reviewer 1

This is a revised version of a previously submitted manuscript by Versteeg et al. on a toolbox combining benchmark tasks, datasets, and measures for assessing dynamical models of neural computation inferred from data.
I'm still generally in favor of this work and appreciate the initiative to create such a benchmark, as well as the thorough revision. I think the paper did improve at lot.
I still have a couple of concerns, however, some from the previous version that I still don't find adequately addressed:

1) The authors added PhaseCodedMemory as an example with simple oscillatory activity, which is appreciated. Yet, there is still not a single example with chaotic activity, although there is mounting evidence that the brain operates in a chaotic regime (almost unavoidable for networks operating in a balanced regime, see for instance famous Nature paper by London et al. 2010). The authors argue that chaotic activity would not offer the reliability and robustness needed for neural information processing, but I disagree as chaotic attractors still have a reproducible statistical structure. They also argue that previous work testing models for inferring dynamical systems focused on poor proxies for dynamics in neural systems, namely mainly examples of 3d chaotic attractors. First of all, I think this misses the point a bit. While I fully agree that benchmarks closer to neuroscience are needed, I believe that for any method developed for DS

identification it needs to be established first that it can indeed faithfully recover the dynamical structure of a variety of systems with different types of dynamical regimes and attractor geometries. And many of the papers cited which then aim to apply this to neuroscience often do go further and examine biologically more plausible scenarios or real data.
*Second, the example benchmarks suggested by the authors are also all quite artificial. It may even be debatable whether and in which sense they are more realistic than a 3d chaotic attractor: Its chaotic dynamics may still be closer to the *dynamics* observed in the nervous system than a model just exhibiting fixed points (like 3BFF), i.e. constant firing rates in the limit. In fact, none of the benchmarks suggested were created from biophysically detailed or spiking neuron model simulations, they are all artificial high-level examples, and in that sense may not be physiologically realistic at all. The aspect that they perform computation on a task is important, however.*
Bottom line: I still think the paper would benefit from inclusion of at least one chaotic example. There are many such neuro-examples out there, like in recent work of Brunel or older work by van Vreeswijk & Sompolinsky. I also think the phrasing needs to be more cautious in some places, in line with the points above, otherwise the paper sometimes sends a bit of a wrong message in my mind.

Response: We agree that chaotic dynamics represent a large and well-studied set of systems that are potentially highly relevant to neural computation, especially in the contexts of learning and sensory processing. Including a dataset that reflects chaotic dynamics could extend the relevance of CtDToolkit to the domain of these types of neural dynamics and better connect with the existing dynamical systems literature. To this end, we have added a new benchmark dataset called ChaoticDelayedMatching based on work in Miconi 2017 (Miconi, T. *Biologically plausible learning in recurrent neural networks reproduces neural dynamics observed during cognitive tasks. eLife* 6:e20899, 2017; `https://elifesciences.org/articles/20899`).

In this dataset, a recurrent neural network with tanh nonlinearities was initialized in a near-chaotic regime via gain-scaled random recurrent weights. The network was then trained to perform a delayed non-match-to-sample task: two sequential binary cues (A or B) were presented, separated by a delay period, and the network was trained to indicate whether the cues match (AA, BB) or differ (AB, BA). This defines a simple and well-specified input-output mapping while requiring the network to maintain and transform information over time; full task and task-trained-model details are given in Appendix B.1.5.

The resulting dynamics exhibit key signatures of chaos in the autonomous or weakly driven regime, including sensitivity to initial conditions as assessed via perturbation-based divergence of nearby trajectories (Fig. S5D,E). Despite these chaotic trajectories, task-relevant variables are still produced correctly during the response period. These trajectories reflect stable computation embedded within high-dimensional, near-chaotic dynamics. This benchmark complements the fixed-point and oscillatory examples in the toolkit by introducing a distinct dynamical regime, one that captures both the variability and computational richness associated with near-chaotic neural systems, while retaining a clear and interpretable task structure.

We also want to be transparent about the nature of this example. As the reviewer notes, the chaotic benchmark we are able to offer is a task-trained recurrent network rather than a biophysically detailed or spiking-network model (such as the balanced-network models of van Vreeswijk & Sompolinsky or Brunel). This reflects a deliberate scope decision: the organizing principle of CtDToolkit is that every benchmark is a dynamical system *optimized to perform a well-specified computation*, which is precisely what lets us pair each dataset with a known ground-truth flow field

and a defined input–output task. Within that scope, ChaoticDelayedMatching supplies what the toolkit previously lacked—a genuinely near-chaotic dynamical regime—and we agree that generating chaotic benchmarks from more biophysically detailed or spiking models would further strengthen the connection to biological neural systems and is a valuable direction for extending the toolkit.

> *2) The authors suggest MMD instead of KL as a measure for assessing geometrical aspects of the dynamics, but I think this is based on a misunderstanding of how KL or Wasserstein distances are actually used in the literature. First, KL and Wasserstein are usually used as ground-truth *independent* measures, unlike how the authors introduce MMD in latent space which requires access to GT. What is commonly done here (see for instance recent review by Durstewitz et al. 2023 and refs. therein) is that a) the observations are delay embedded, b) the latent activity is projected into that same latent space via the used decoders. The problems of invariance to affine transformations or different dimensionalities thus do not occur the way these measures are commonly used on empirical data.*
> *Second, KL and Wasserstein distances are well established and tested out in the dynamical systems lit., while MMD is not to my knowledge, and at least I personally have little intuition on how it behaves for different types of attractors and system dynamics. I therefore think for KL/Wasserstein one would be on safer ground with more experience from a lot of previous work. Alternatively or additionally, for temporal structure autocorrelation functions or power spectra are often used. Besides, the MMD would need a lot more explanation (what is ν for instance?) and concrete examples that show how it behaves and scales for different degrees of geometrical overlap in DS settings.*

Response: We thank the reviewer for this suggestion. In response, we have incorporated Wasserstein distance as the primary geometry-based distribution-matching metric in the CtDToolkit, implemented in a manner consistent with prior work. We agree that Wasserstein distance is well established in the dynamical systems literature and provides an intuitive, ground-truth-independent comparison.

In the revised manuscript, we replace MMD with Wasserstein distance in all figures to align with these established practices. We retain MMD as part of the Toolkit for use in settings where ground-truth latents are available, but we no longer emphasize it in the primary results. We have also clarified the description of all geometry-based metrics in the Methods (Section 2.4.2; Appendix E.5).

> *3) I don't quite understand the 'non-injectivity' argument, as it runs counter to the usual logic in DS theory where – just to the opposite of what the authors recommend – the representations or models are deliberately *expanded* (of much higher dimensionality) than the empirical data. This is the essence of the delay embedding theorems: We aim for a diffeomorphism between sets in the *true* underlying space in which the dynamics lives and the reconstructed space, and *not* literally between the latent space and the actual observations which usually only represent a tiny portion of all dynamical variables governing the observed system. Although the issue of models introducing spurious features is certainly a very important one, and although the general idea may be correct that over-parameterized and more complex models are more likely to introduce such features, the logic of the injection principle as described and specifically implemented in the paper in my mind is somewhat flawed. I suspect that it only works for the kind of toy models the authors examine with access to *all* dynamical variables, but, as stated*

in the paper, would break down on empirical data.

Response: We agree with the reviewer that, if the goal is to reconstruct the full autonomous dynamical system governing the observations, then expanding the state through delay embedding or related approaches can be appropriate. However, a central ambiguity in neural dynamical systems identification is whether apparently "tangled" trajectories reflect unobserved state variables of the autonomous system, or instead reflect unobserved external inputs driving the recorded population. Because strong external drive is ubiquitous in the brain, expanding the model state may inadvertently shift the object of study from the intrinsic dynamics of the recorded population to a larger system that includes upstream areas, sensory feedback, task variables, or broader circuit-level dynamics.

The injectivity principle in our work is therefore not intended as a general argument against state-space expansion, nor as a replacement for delay embedding when the goal is full-system reconstruction. Rather, it is a modeling constraint for cases where the scientific goal is to infer interpretable dynamics of a particular observed neural population. In this setting, injective observation models make an explicit assumption: that the relevant state variables of the modeled population are reflected in the recorded activity. When this assumption fails, the appropriate interpretation may be that the population receives unobserved external inputs, rather than that these variables should necessarily be absorbed into an expanded latent state.

We recognize that this distinction was not made clearly enough in the original manuscript. We have revised the text to clarify the scope of the injectivity argument, to acknowledge its limitations, and to emphasize that injective observation models have their own drawbacks. In particular, when important dynamical variables are unobserved, injectivity alone cannot guarantee recovery of the true dynamics; it instead helps prevent models from explaining away missing inputs by introducing latent features that are not reflected in the recorded neural activity. This motivation is consistent with the manuscript's distinction between feature invention and dynamical misattribution as separate failure modes of data-trained dynamics models (Section 1.2.2).

We have also clarified that the property that matters for this argument is the *functional* injectivity of the *learned* observation model on the data, rather than an architectural injectivity constraint imposed on the readout (see Section 2.4.2 and Appendix E.4). Hard architectural constraints such as injective MLP readouts are one way to encourage this, but unconstrained networks may also learn functionally injective mappings by virtue of training. This framing further weakens any claim that a particular architectural choice is universally preferable.

We have softened the original language to avoid implying that injectivity is universally preferable to state expansion.

I don't want this to come across too negative, I in fact appreciate the authors' efforts to introduce a common standard or basis into the field, and one certainly needs to make a start somewhere! But I find the points above important, and I hope the authors will see what I mean and could consider in their revision one chaotic example and possibly measures better established in the DS identification literature.

Minor points

> *The authors expanded their f:I→O definition of computation. What this definition still misses in my mind is a notion of internal states as crucial in most concepts of computation like Turing machines or finite state machines. Left up to the authors, just wanted to mention this.*

Response: We agree that internal states are essential for performing a computation, but argue they are not specified by the computation itself. For example, the computation SORT defines an input-output mapping from lists to sorted lists, yet can be implemented by many algorithms that differ in their required sizes of internal states and trade off memory and computational complexity. We view it as a feature of our definition that it remains agnostic to these implementation details, specifying only the input-output transformation, and allowing internal states to be reflected as part of the "algorithm" implementing that computation.

> *I still would discourage the use of the work "embedding" for a projection from the latent into the neuronal space, as this conflicts with common usage in ML and DS. How about "neural encoding" or "representation" or similar?*

Response: We thank the reviewer for this suggestion and agree that the term "embedding" may be confusing in this context. We have revised the manuscript to use "observation model" to refer to the mapping from latent states to neural activity, which is more consistent with common usage in the dynamical systems and state-space modeling literature.

> *Since so much in this article is viewed in terms of dynamics, I still find it a bit of a pity that it interfaces so little with the rich literature on dynamical systems reconstruction.*

Response: We thank the reviewer for this suggestion and agree that stronger connections to the dynamical systems reconstruction (DSR) literature would improve the manuscript. In response, we have made this connection explicit in two places. In the Introduction, we now note that the DSR field has developed a mature set of evaluation measures for validating inferred dynamics against known ground-truth systems on canonical (non-neural) attractors such as Lorenz and Rössler, and we frame a central aim of this work as adapting and building upon those tools for the additional challenges of the neural setting (unknown external inputs and goal-directed task structure), rather than reinventing them.

We have also added a paragraph to the Discussion (Section 4) that positions CtDToolkit relative to this body of work. There, we note that the DSR community typically validates reconstructions using measures of *invariant* structure rather than trajectory-by-trajectory prediction error: geometric measures such as the Wasserstein distance between distributions of visited states, and temporal measures such as power-spectrum or autocorrelation agreement, typically computed in a shared, observation-derived (and often delay-embedded) space that does not require ground-truth latents or trial-by-trial alignment. We explain that our newly added Wasserstein distribution-matching metric brings this distributional perspective directly into the Toolkit (consistent with our response to point 2 above), and we identify the complementary temporal measures as a natural next step. Together with the Wasserstein metric and the full Lyapunov-spectrum tools added in this revision, these additions substantially strengthen the manuscript's interface with the DSR literature.

I'm not sure the term "under-fitting" describes best the issue LDS faces on many data: They simply cannot represent the dynamics at all, regardless of how many parameters.

Response: We thank the reviewer for this clarification. We agree that the limitation of linear dynamical systems (LDS) in this context arises from a structural inability to represent the underlying dynamics, rather than from insufficient parameter tuning within the model class.

In the manuscript, we used the term "underfitting" in a broad sense to refer to models that fail to capture the neural observations. We agree, however, that it is useful to distinguish between different sources of this failure. In particular, LDS models can exhibit what may be more precisely described as structural underfitting, where the class of representable dynamics does not include the target system, regardless of parameter values. This contrasts with more expressive models (e.g., RNNs), which may underfit due to limited capacity (e.g., insufficient latent dimensionality) despite being, in principle, capable of representing the true dynamics.

We would add that, for these more expressive models, latent dimensionality is only one of many choices that determine effective capacity. This connects to a related concern raised by Reviewer 2 (major comment 2), who cautioned against framing latent state dimensionality as the only important hyperparameter. We agree: regularization strength, input penalties, training duration, architecture, and optimization settings can all move an expressive data-driven model between underfitting, faithful recovery of the true dynamics, and the invention of spurious features. We have therefore revised the manuscript to treat capacity-limited underfitting as one point within this broader hyperparameter landscape rather than as a property governed by latent dimensionality alone (Section 3.2), and we refer the reader to our response to Reviewer 2's major comment 2 for a fuller discussion.

We have revised the manuscript to clarify this distinction and adopted more precise terminology when referring to limitations of LDS models (Section 3.1).

For me the LDS results still suggest that RandTarget is the simplest, not the most complex of the benchmarks introduced, perhaps only requiring linear regression from inputs to outputs?

Response: We thank the reviewer for this observation and agree that this is an important point. We do not intend to claim that the intrinsic dynamics of the RandomTarget task are necessarily more complex in a representational sense than other canonical datasets. Rather, the challenge posed by this dataset arises from the strong coupling between external inputs and the system's dynamics.

In RandomTarget, the observed activity reflects a regime in which input-driven and recurrent contributions are highly entangled, making it difficult to disentangle intrinsic dynamics from input effects. As a result, even if the underlying input-output mapping could in principle be captured by a relatively simple model (e.g., linear regression), recovering the correct dynamical decomposition, separating autonomous dynamics from inputs, is substantially more challenging. This is the sense in which we refer to the task as "complex" from the perspective of dynamical inference.

We have revised the manuscript to clarify that the difficulty of RandomTarget lies in this input-dynamics confounding, rather than in the intrinsic complexity of the autonomous dynamics alone.

Not sure Fig. 6E is topologically possible: If the map defining the underlying DS is smooth and invertible (and since chaos is absent here), there always would need to be saddles separating the fixed points (Smale-Morse).

Response: We would agree with the reviewer's point if this system were autonomous. The apparent attractors arise from the effect of inferred external inputs, which drive the system toward those locations. As a result, these points are not fixed points of the autonomous flow field, and the Smale-Morse principles do not apply in this setting. We have revised the text to clarify this distinction (Fig. 6E).

> *In some places I missed a bit more precision in the terminology, for example:*

> *p.12: Fixed points are mathematically defined as points where the flow completely vanishes (is *exactly* zero), not when it is "sufficiently slow". And the linear "approximation" (actually it is a topological equivalence) holds only for hyperbolic systems for a sufficiently small environment around the fixed point (not related to "slowness").*

Response: We thank the reviewer for this clarification and agree with the formal definition of fixed points and the conditions under which linearization results apply. Our intent was to describe regions of state space that behave as effectively stable over behaviorally relevant timescales, rather than to redefine fixed points in the strict mathematical sense. We have revised the text to distinguish clearly between true fixed points (where the flow is exactly zero) and "functional fixed points," i.e., regions in which the dynamics are sufficiently slow or contracting to appear stable over the timescales relevant to behavior (Section 2.4.3).

> *p.12/13: The overall stability properties of the system are not described by a single Lyapunov exponent but by the whole Lyapunov spectrum (which can also easily be computed from the Jacobians). For instance, a chaotic set can still be an attracting set if the sum of all Lyapunov exponents is smaller than 0. The virtue of the *maximal* Lyapunov exponent is that this can usually also be assessed from real data, hence directly compared and used as quality stats.*

Response: We thank the reviewer for this clarification and agree that the stability properties of a dynamical system are fully characterized by the Lyapunov spectrum, rather than by a single exponent. In particular, we agree that chaotic dynamics can still correspond to an attracting set when the sum of Lyapunov exponents is negative.

In this work, we focus on the maximal Lyapunov exponent because it provides a practically accessible summary statistic that can be estimated from data and compared across models. Our intent was not to suggest that it fully characterizes system stability, but rather to use it as a coarse diagnostic of sensitivity to initial conditions.

To address this point more directly, we have extended the Lyapunov exponent estimation tools in CtDToolkit to compute and return the full Lyapunov spectrum, rather than only the maximal exponent. Concretely, we added a documented `compute_lyapunov_spectrum` routine (and a corresponding `Analysis_TT.compute_lyapunov_spectrum` method) that returns the complete ordered set of exponents together with summary statistics, including their sum (the mean rate of phase-space volume contraction) and the Kaplan-Yorke dimension. This lets users distinguish, exactly as the reviewer notes, between sensitivity to initial conditions (positive maximal exponent) and the existence of an attracting set (negative sum of exponents).

To verify that this estimator is correct, we added a worked example (`examples/figures/supplementary/lyapunov_spectrum_lorenz.py`) that recovers the full spectrum of the canonical Lorenz system from its Jacobians. The estimated spectrum, $[+0.91, -0.01, -14.56]$, closely matches the literature reference $[+0.906, 0, -14.572]$; the maximal exponent is positive (chaos) while the sum, ≈ -13.67, is strongly negative, confirming that the Lorenz attractor is a chaotic *attracting* set (Kaplan-Yorke dimension ≈ 2.06). The same example reproduces the Rössler spectrum as an additional check.

We have revised the manuscript to clarify the distinction between the maximal Lyapunov exponent and the full spectrum, to note that the maximal exponent is reported because it is the component most directly estimable from data, and to document this additional functionality (Appendix E.6).

Reviewer 2

> *I thank the authors for the careful revisions of the manuscript, which have addressed most of the confusion points raised in the previous review. In particular, the goals of the CtDToolkit as a platform for model development and testing are now clear. The current manuscript is also more explicit about the fundamental ambiguity in attributing the observed activity to dynamics versus inputs. These revisions substantially improve the framing of the CtDToolkit and its grounding in neuroscience and the broader dynamical-systems inference literature. However, several issues have not been addressed in full and require further clarifications, as detailed in the specific comments below. Addressing these issues is important, as CtDToolkit is intended to become a widely used tool, aimed at consolidating the field around common standards of model evaluation. Therefore, it is critical to ensure that the logic and philosophy guiding such model evaluation are sound and capable of fostering consensus within the community.*

Response: We thank the reviewer for the kind words and agree on the importance of clarity of objective for this research area: we need to agree on our goals for us to be able to agree on the best steps to take towards those goals.

Major comments

> *1. The paper is inconsistent about the purpose of the evaluation metrics. The text states at multiple points that these metrics should "guide model selection". In other places, it states that these metrics are used only for "post-hoc assessment of previously trained models", contrasting it with the model selection approach from Ref. [38]. This distinction is confusing. What is the purpose of "post-hoc assessment" if not to select the optimal model for a specific dataset? Indeed, the description of the application of these metrics to example datasets (Figs. 5,6) reads exactly as a model selection procedure: some models are underfitted, some are overfitted, and some are just right (according to the metrics). How is the "post-hoc assessment" different from model selection?*

Response: We apologize for the confusion on this point: to clarify, we consider both 1) metrics computed post-hoc on individual models and 2) methods that use across-model feature consistency

to be complementary approaches to the problem of model selection. We have clarified the role of these model selection approaches in the text (Section 4).

> *2. The paper is inconsistent about the description of hyperparameter tuning. Returning to the stated contrast between "post-hoc assessment" and what is described in the manuscript as "training-phase interventions" in the context of Ref. [38], this framing is misleading, as both approaches are model selection procedures aimed at identifying hyperparameters that yield models most closely matching the true dynamics. For sufficiently expressive DD models, model selection amounts to choosing optimal hyperparameters: latent state dimensionality and regularization strength. The manuscript illustrates how the evaluation metrics can be applied to select both types of hyperparameters: latent state dimensionality (Fig. 5) and regularization strength on inputs (Fig. 6). Similarly, the model selection procedure in Ref. [38] concerns with choosing the regularization strength on model complexity: a training epoch for early stopping or the weight of model complexity penalty. Expressive models trained via gradient descent learn features of increasing complexity across training epochs. Therefore, the number of training epochs is a critical hyperparameter that can result in under- or overfitting. However, the description of results in Fig. 5 glosses over this issue: all models were trained for a fixed number of epochs chosen ad hoc and this choice is not discussed, making a misleading impression that the latent state dimensionality is the only important hyperparameter. To be clear, I do not suggest to implement the model selection metrics from Ref. [38] within the CtDToolkit nor to change the results in Fig. 5. Rather, I encourage the authors to describe the problem of hyperparameter selection consistently, holistically, and transparently. Since CtDToolkit aims at a broad audience of model developers with varying amount of expertise, who may not be able to read between the lines, it is important to avoid misleading distinctions, such as between training-phase versus post-hoc assessment, and to be upfront about all different kinds of hyperparameters critical for identifying correct dynamics with expressive models.*

Response: We agree that our previous framing was not sufficiently general. We have revised the manuscript to emphasize that latent dimensionality is only one of many hyperparameters that can affect model performance on CtDToolkit metrics (Section 3.2). Regularization strength, input penalties, training duration, architecture choices, and optimization settings can all influence whether an expressive model underfits, recovers the relevant dynamics, or invents spurious features. We reference [38] as an example of how the number of training epochs can dramatically affect the fidelity of the inferred dynamical landscapes, even while generalization performance remains high.

> *3. As mentioned in the previous review, injective mappings do not generally prevent inferring spurious features in the dynamics (shown in Ref. [38]). While I agree with the authors' response that an injective mapping is preferred over a degenerate one, the insufficiency of injective mappings has not been addressed in the manuscript. To the contrary, the text suggests at multiple points that injective mappings are sufficient (e.g., statements like "Alone, reconstruction and simplicity are each insufficient to judge DD model quality, but together they can provide interpretable accounts of DD model performance when external inputs are known" or the last paragraph on page 11). The issue with this argument is that it reasons about "predicted neural activity" missing the distinction between firing rates and spikes. Spurious features in latents can be predicted from firing rates if the mapping is injective, but these features may*

not be detected by reconstruction metrics defined on spikes. There may be indications of this effect in Fig. 5E,G,H. For NODE-64, state R2 appears much lower than cycle consistency, suggesting that inferred latents have features predictable from rates but not present in the true latents, without affecting reconstruction metrics (rate R2 and co-BPS). Since both rates and latents are unobserved quantities, their consistency does not generally guarantee the correctness of the inferred dynamics.

Response: We agree that injective mappings are not sufficient to prevent the inference of spurious latent features. Our intent was to describe injectivity as a useful constraint relative to non-injective readouts, not as a guarantee of correct dynamical inference.

Injectivity ensures that latent features cannot be completely hidden in a readout null space: changes in latent state must be reflected in the model-predicted firing rates. However, as the reviewer correctly notes, predicted firing rates are not directly observed. A model may introduce additional latent structure that produces small or noise-level changes in predicted rates, such that these features are not strongly penalized by spike-based reconstruction metrics such as co-BPS. Thus, consistency between inferred latents and inferred rates does not generally imply that the inferred dynamics are correct, particularly because both latents and rates are unobserved quantities.

We have revised the manuscript to clarify that injectivity promotes observability of latent features but is neither necessary nor sufficient to rule out spurious dynamics. We also now distinguish between the *functional* injectivity of the learned observation model on the data (which is what cycle-consistency actually measures) and architectural injectivity constraints on the readout. Hard architectural constraints are one way to encourage functional injectivity, but an unconstrained MLP readout can also happen to learn a functionally injective mapping over the course of training. This framing clarifies that cycle-consistency is a diagnostic on the trained model rather than a property of the chosen architecture (Section 2.4.2; Appendix E.4). We now emphasize that injective readouts should be understood as one modeling constraint among several, and that reliable dynamical inference requires complementary diagnostics, careful model selection, and, where possible, validation against ground-truth or perturbation data.

4. Related to the previous point, the true rates in Fig. 5B exhibit high-frequency features. What is the source of these features? Do they arise from noise injected in the TT models? If so, a model capable of predicting random fluctuations should be considered overfitted. While NODE-8 clearly predicts the high-frequency features better than NODE-3 (as confirmed by the rate R2), are these predictions meaningful if they reflect random noise rather than task-relevant dynamics? Since NODE does not model stochastic dynamics (i.e. the distribution over latent trajectories on each trial), it may be especially prone to this type of overfitting. This raises a question of whether the rate R2 may be contaminated by the reconstruction of noise. If so, it becomes unclear how much improvement in reconstruction (along the y-axis in Fig. 5I) do we actually want from the optimal model? In other words, what direct evidence supports that NODE-3 is underfitting rather than NODE-8 overfitting to noise in the TT model rates?

Response: The high-frequency structure in Fig. 5B arose from two primary sources: noise in the external inputs, which are supplied to both the task-trained and data-trained models, and settling transients following bit transitions. These components are not private stochastic fluctuations in the task-trained latent state that the data-trained model should be unable to predict, but input-driven and/or transient features of the task-trained model's firing rates in response to noisy pulsatile

inputs. We agree that this distinction was not clear in the original manuscript, and do not want to overemphasize the ability of our models to reconstruct trial-specific fluctuations induced by input noise.

For this reason, and as much of the improved performance of NODE-8 over NODE-3 reflected a failure to capture settling transients, we agree that the original examples did not provide a sufficiently clear demonstration of underfitting. We therefore re-ran the model sweep to include a 2D NODE model. We found that 2D NODE suffered from a more pronounced underfitting that the 3D NODE, providing a clearer example of how insufficient model capacity can lead to poor reconstruction than was included in the original submission.

> *5. There are several technical issues In Fig. 5 related to previous points. (i) Fig. 5F shows that the state R2 and cycle consistency have nearly identical values across models (points on the diagonal), whereas Figs. 5G,H make it clear that cycle consistency is systematically higher than state R2 across models. Which data are correct? They cannot be correct simultaneously. (ii) In Fig. 5E, the prediction from the true latents for NODE-64 appears to show a systematic bias (the two time series have different means), which is unexpected for an affine transformation. What is the issue here? (iii) What are the ticks at the bottom of Fig. 5B? What are the time units here relative to the timescale of the task? (iv) In Fig. 5C,F,G,H it would be helpful to indicate all models of the same size with the same color, and highlight the models shown in panels B,E with a distinct symbol. In the current presentation, it is hard to assess the spread across models of the same size.*

Response: We thank the reviewer for identifying these technical issues. In addition to re-running the model training sweep for point 4, we have corrected the relevant panels and revised the figure-generation workflow to prevent inconsistencies across panels.

i) The reviewer is correct that the values in the previous Fig. 5F were inconsistent with those shown in Fig. 5G,H. Some panels were vestigial from an earlier version of the figure and were not regenerated from the same model set. We have now re-run the relevant analyses and updated the figure so that all panels are generated together from the same source data. The qualitative interpretation of the results is unchanged, although the specific plotted values have changed.

ii) The apparent bias in the NODE-64 prediction in Fig. 5E arose (and is still present in the 32D NODE model used for the resubmission) because the affine transformation from task-trained latents to data-trained latents was fit across all trials, whereas the panel shows only a single example trial. The baseline shift in this example reflects across-trial drift in the data-trained model latent activity that is not present in the task-trained model. This is an example of the kind of invented latent structure that the simplicity metrics are intended to diagnose.

To illustrate this point, Response Fig. 1 shows example trials of the 32D NODE model sorted by the per-trial mean of (DD actual − TT→DD affine prediction) for PC8, from negatively biased (left) to positively biased (right). As can be seen, there is a strong per-trial bias in PC8 of the DD model (solid) that cannot be predicted by the affine projection of the TT latents (dashed). This "extra" dimension of variance not present in the TT latents illustrates the danger of invented features potentially making the inferred latent activity untrustworthy.

iii) We have added explicit time-scale labels to Fig. 5B so that readers can interpret the duration of the plotted trial segments relative to the task timescale.

iv) We have revised the marker style for Fig. 5. Models with the same latent dimensionality are now shown with the same color across panels, and the models highlighted in the example panels

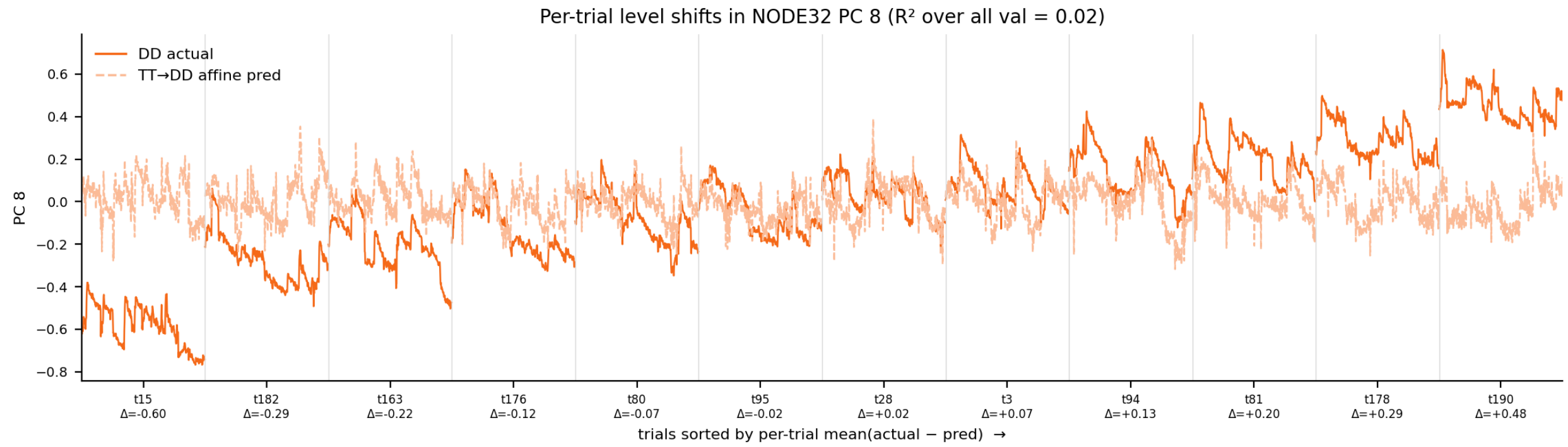

Figure 1: Per-trial level shifts in the 32D DD-NODE model's PC8 (NBFF Canonical dataset). Each segment is one held-out validation trial, sorted left-to-right by the per-trial mean residual between the DD model's PC8 (solid orange, "DD actual") and the best affine prediction of PC8 from the task-trained latents (dashed, "TT→DD affine pred"). The overall R^2 across all validation time points is 0.02. The systematic across-trial offsets in the DD latent are not captured by the affine map from the TT latents, indicating latent structure invented by the DD model.

are marked with distinct symbols. Specifically, the callout models are shown with circles, while the remaining models are shown with X's. This makes the spread across random initializations within each model size easier to assess.

> *6. Many of the issues raised above stem from the fact that all metrics are computed on latents and rates, rather than directly assessing the accuracy of reconstructing the ground-truth flow field. Any error metric between the inferred and ground-truth flow fields would not suffer from problems such as overfitting to noise in the true rates or Poisson noise. While such a metric is not trivial to compute for the TT model architectures chosen here, it would be relatively straightforward for other architectures, e.g., low-rank RNNs. To be clear, I do not suggest to introduce additional TT model architectures in the CtDToolkit. It would be sufficient to acknowledge that many problems arise due to the lack of direct comparison with the ground-truth flow field, which can be addressed in the future using different TT model architectures.*

Response: We thank the reviewer for this insightful comment and agree that direct comparison between inferred and ground-truth flow fields would provide a less ambiguous assessment of dynamical accuracy. In particular, such comparisons would avoid confounds arising from observation noise or input-driven variability, which can affect metrics defined on rates or spikes.

We have revised the manuscript to explicitly acknowledge the limits of metrics evaluated on individual trajectories rather than the flow field itself, and to highlight that, for TT model architectures where the flow field can be directly evaluated (e.g., low-rank RNNs), direct comparisons between inferred and true vector fields would be a valuable direction for future work (Section 4).

> *7. The need for co-smoothing metric is specific to the DD model architectures considered here, which are based on sequential autoencoders that require access to neural data to make predictions even in validation and testing. For DD models that do not require spike data as input, a likelihood computed across all neurons on held-out trials is sufficient to assess the prediction*

accuracy and should be preferred over co-smoothing. Although co-smoothing is necessary to prevent autoencoders from learning trivial identity maps, this metric uses less data for training and testing and hence is noisier, and it is also sensitive to which neurons will land in the held-in and held-out sets. Since CtDToolkit targets a broad model-development community, this issue should be acknowledged because not all DD models are autoencoders.

Response: We thank the reviewer for this clarification and agree that the need for co-smoothing is specific to autoencoder-based DD model architectures that condition on observed neural activity at inference time. In such models, co-smoothing is necessary to prevent trivial identity mappings and to ensure that predictions reflect meaningful latent structure rather than direct copying of inputs.

We also agree that for DD models that do not require neural activity as input at inference (e.g., models that generate activity purely from latent dynamics and inputs), standard likelihood-based evaluation on held-out trials is sufficient and should be preferred, as it makes use of all available neurons and avoids the additional variance introduced by held-in/held-out splits.

In the current manuscript, co-smoothing is used to provide a consistent evaluation framework across the class of autoencoder-based models considered. However, we agree that this choice should be better contextualized, particularly given that CtDToolkit is intended to support a broad range of model architectures.

We have revised the manuscript to clarify that co-smoothing is not a universal requirement, but rather a metric tailored to a specific class of models, and to note that likelihood-based evaluation is more appropriate for models that do not condition on observed spikes at inference (Section 2.4.2).

8. The previously raised concern about the assumption of linear embeddings has not been addressed in full. Several related issues remain.

8.1. The fact that all experiments use linear mapping between latents and log-rates should be stated upfront in the main text (as in the original submission), as this became less apparent in the revised manuscript.

Response: We have restored the statement of the assumption of linear (linear–exponential) observation models in the main text, and we now discuss this assumption explicitly as a limitation in the Discussion (Section 4), where we emphasize the need for further work on metrics that are robust to relaxing the assumption of a known observation model $g(\cdot)$.

8.2. The fact that nonlinear cycle consistency yields similar results to linear cycle consistency under the canonical embedding (Fig. S8) does not validate that nonlinear cycle consistency produces good results under nonlinear embeddings. What is the purpose of this analysis and has the nonlinear cycle consistency been validated under nonlinear embeddings?

Response: A version of the non-linear cycle-consistency metric has been previously published (Versteeg et al., *Expressive dynamics models with nonlinear injective readouts enable reliable recovery of latent features from neural activity.* Proceedings of Machine Learning Research, vol. 228, pp. 255–278, 2024; `https://proceedings.mlr.press/v228/versteeg24a.html`, Figure 3). In this work, we

showed that when simulated data are non-linearly embedded into neural firing rates (e.g. using a sigmoidal activation function), injective non-linear readouts are able to capture the embedding non-linearity and prevent invented features, in contrast with standard MLP readouts. This manifested as an increased noise robustness of the learned generalized inverse $g_\phi^\dagger$ (the MLP-based approximation of the inverse of the observation model $\hat{g}$, in the notation of the appendix), leading to higher non-linear cycle consistency R2.

> *8.3. The nonlinear cycle consistency is based on fitting MLP, which is an expressive model with many hyperparameters (I couldn't find these hyperparameters in the paper). How are these hyperparameters tuned for each model? How do we know that the loss of robustness to noise arises from a non-injective embedding rather than from a brittle, overfitted MLP?*

Response: We have revised the supplementary materials to include the MLP hyperparameters and training / validation loss trajectories for the NL cycle-consistency results shown (Fig. S9C). We saw no evidence of overfitting in the learned inverse mapping: the training and validation loss trajectories tracked closely across all latent sizes. We have added additional guidance for how to use the non-linear cycle consistency while avoiding overfitted inverse models.

> *9. The concern about the use of linear metrics remains, as it also applies to state R2. Any nonlinear change of variables in the latent dynamical system and its embedding yields an equivalent model. Linear state R2 works in the example applications here because the embedding is fixed to be linear, eliminating nonlinear changes of variables. This issue has not been addressed in revision or response letter.*

Response: Per our response to point 8, we have restored the statement of the assumption of linear embeddings in the main text and now discuss it explicitly as a limitation in the Discussion (Section 4), where we emphasize the need for further work on metrics that are robust to relaxing the assumption of a known embedding $g(\cdot)$.

> *10. The description of data generation from TT models is unclear. "The activity of each of these sampled units is standardized by subtracting the mean of each unit individually, and then normalizing by the scaled standard deviation across all units". What is "scaled standard deviation across all units"? Was activity of all units normalized by the same number or different numbers? Does this mean each unit's activity was z-scored? Since firing rates in neural recordings are heterogeneous, what is the rationale for z-scoring the activity of all units? What is the purpose of the fr_scaling parameter?*

Response: We thank the reviewer for pointing out this point: the original description was inaccurate. Simulated neural activity is not z-scored on a per-unit basis. Instead, after selecting latent dimensions to serve as simulated neurons, we compute a single global mean and a single global standard deviation across all trials, time points, and units, and transform activity by subtracting this global mean and dividing by (fr_scaling multiplied by the global standard deviation). All units are normalized using the same mean and the same standard deviation, rather than unit-specific

statistics. Therefore, this process does not affect the relative magnitudes of the synthetic firing rates from their origins in the latent states of the TT model.

Finally, we note that when target_mean_rates is specified, an alternative procedure is used in which per-neuron biases to the pre-link firing rates are fit to achieve desired mean firing rates after the link function, rather than applying the global normalization described above. This branch was used only for the PhaseCodedMemory task, for which we wanted to create a population of neurons all with average firing rates ∼1 Hz. We further note that the DD PhaseCodedMemory validation figure shows the top 3 PCs of the ground-truth activity in both firing-rate space and latent space (Fig. S16A, columns 2 and 3), which exhibit the same qualitative ring structure. This demonstrates that the general geometry of the rates and latents is not distorted by this latent-to-rate transformation, though we acknowledge that this similarity may be partially obfuscated once the rates are passed through the noisy Poisson spiking process.

We have revised the manuscript to clarify these points and to more accurately describe the data generation procedure.

11. The seemingly innocuous latent z-scoring prior to data generation may introduce artifacts, such as an apparent failure of the DD model on the PhaseCodedMemory dataset. If the TT model contains some units with low variance and small differences in mean activity between two conditions, these units contribute negligibly to the total variance, such that the shift between the two ring centroids is virtually invisible in PCs of the true latents (Fig. S3D). These low-variance units may also contribute negligibly to the TT model output, showing low-variance fluctuations with minimal task modulation. However, after z-scoring, their contribution will be up-weighted while the contribution of task-modulated high-variance units will be down-weighted, which will amplify the shift between the ring centroids in z-scored latents. As a result, DD models trained on the scaled latents will also exhibit a shift between the ring centroids. This scenario should be regarded as a failure of data generation (or comparison with wrong latents), not a failure of the DD model. Although it is hard to tell from the text whether this scenario actually takes place, as the description of the results and analyses is incomplete, it is plausible. The TT model has 512 units and is trained with an L2 penalty on the latents, making it plausible that the model contains many low-variance, task-irrelevant units. In addition, the numbers reported in the text appear inconsistent: Table S10 indicates that the dataset includes 500 held-in and 100 held-out units, whereas the TT model contains only 512 units.

Response: The PhaseCodedMemory dataset uses the target_mean_rates pathway, not the global fr_scaling standardization pathway. In this branch, simulated neurons are generated by copying latent dimensions and fitting per-neuron additive biases so that, after the chosen observation non-linearity, each neuron achieves a desired mean firing rate. Thus, this branch does not z-score latents or reweight low-variance dimensions by variance normalization, and so it is unlikely to suffer from the artifact described in comment 11.

We also thank the reviewer for catching the apparent numerical inconsistency between the 600 simulated neurons (500 held-in + 100 held-out; Table S10) and the 512 units of the task-trained model. This is expected behavior of our neural-simulation pipeline rather than an error: the number of simulated neurons is intentionally decoupled from the dimensionality of the task-trained latent state. When the requested neuron count exceeds the latent dimensionality, the simulator builds the observation (readout) matrix by stacking independent random permutations of the latent indices and keeping the first N (here $\lceil 600/512 \rceil = 2$ stacks, from which the first 600 of 1024 indices are retained;

see `NeuralDataSimulator` in `/ctd/task_modeling/simulator/neural_simulator.py`). Each simulated neuron is assigned to a single latent dimension via a one-hot column of this readout, passed through the observation nonlinearity, and then sampled with its own independent (pseudo-)Poisson draw. Consequently, when N exceeds the latent dimensionality, a subset of latent dimensions (here 88 of the 512) is observed by more than one neuron. Because the firing rates are low, the independent Poisson sampling of these shared dimensions produces effectively distinct, noisy "views" of the same underlying rate rather than identical duplicate neurons. The reported 600 neurons versus 512 latent units is consistent with the intended data-generation procedure.

> *12. Overall, the presentation of the PhseCodedMemory task is underwhelming. The conclusion that it "provides a striking demonstration of how naively trusting dynamics learned by a DD model could lead researchers to incorrect conclusions" is vague and uninformative in the absence of any performance metrics. What does it mean to "naively trust" the dynamics? Not using any performance metrics at all? Without any intuition about the mechanism causing the DD model failure, this result is also impossible to interpret.*

Response: We thank the reviewer for pushing us to make this result concrete and interpretable, and we agree that, as originally written, the claim leaned on an undefined notion of "naively trusting" the dynamics without the quantitative support needed to interpret it. By "naively trusting" a DD model we mean reading the geometry of its *inferred latent state*—trajectory structure, fixed points, or single-unit tuning—as the computational strategy of the underlying system without cross-checking that geometry against a ground truth (available here in simulation) or against the simplicity / cycle-consistency metrics we advocate (the only recourse with real data). We have softened the "striking demonstration" phrasing in the main text accordingly.

To support the result quantitatively, the revised appendix (Appendix E.8, Fig. S16) now reports both a reconstruction metric and a state metric across a sweep of LFADS latent sizes (8D, 16D, 128D), and the two *dissociate*: Rate R^2 improves with capacity ($0.68 \rightarrow 0.73 \rightarrow 0.77$) while State R^2 degrades ($0.97 \rightarrow 0.95 \rightarrow 0.92$). A practitioner selecting on reconstruction alone would therefore be most confident in exactly the regime where the inferred latent geometry is most misleading. The failure is thus not an "absence of any performance metric" but a case in which the reconstruction and simplicity metrics *disagree*—precisely the situation CtDToolkit is designed to surface.

The mechanism behind this disagreement is a divergence between *rate-space* geometry (what is observed) and *latent-space* geometry (the model's internal solution). In the ground-truth TT model the two agree: in both views the Stim A and Stim B trajectories form overlapping rings that are primarily *rotated* relative to one another (phase coding). The DD models reproduce this rate-space geometry (overlapping rings, consistent with the high Rate R^2) but infer a qualitatively different latent geometry in which the two conditions are spatially *separated* (rate coding)—reaching the correct observations through an incorrect mechanism that is visible only when the latent geometry is compared against ground truth. As detailed in our response to comment 11, this divergence is not an artifact of data generation: the PhaseCodedMemory dataset uses the `target_mean_rates` pathway, which fits per-neuron biases to achieve target firing rates and does *not* z-score or variance-reweight the latents. The representational difference is therefore a genuine property of the fitted DD model rather than a consequence of the simulation procedure.

13. I agree that current TT models are task-computation focused, but referring to them in the text as "biologically plausible" is misleading. These GRU architectures with tanh activation do not compute through biologically-plausible non-negative firing rates. The firing rates are obtained separately through additional transformations of the GRU activity. It would be appropriate to remove all references to the biological plausibility of these models.

Response: We thank the reviewer for this clarification and agree that the term "biologically plausible" is inappropriate in this context. The TT models used here are not intended to faithfully capture the biophysical mechanisms of neural computation, but rather to provide task-performing dynamical systems with some qualitative similarities to neural activity. We have revised the manuscript to replace "biologically plausible" with "biologically motivated" throughout.

14. The results in Fig. S7 are unclear and their description is confusing and incomplete. Fig. 5E indicates the NODE infers the correct number of stable fixed points, whereas GRU infers many more fixed points than in the TT model. Nevertheless, the DSA metric indicates that the GRU is closer to the true model than NODE. What is the source of the apparent discrepancy here? Section E.3.1. is cryptic and impossible to understand.

Response: We thank the reviewer for pointing out the lack of clarity in this analysis. We agree that the previous figure, which was part of the main text in an earlier submission, had become largely vestigial: many of its points (e.g. those regarding rate and state R2) have since been incorporated into other figures of the main text.

For this reason, we have re-run the DSA analysis using a sweep of models with both NODE and GRU generators. We computed the DSA both between the model-inferred latent activity and the ground-truth task-trained (TT) latents, and between each DD model and every other DD model within the same model class (GRU or NODE).

We found that models which inferred qualitatively accurate fixed-point structures were also found to be more similar to one another by the DSA metric. In other words, the DSA-based groupings were consistent with the fixed-point analyses, resolving the apparent discrepancy noted by the reviewer.

We feel that this updated analysis is a good demonstration of how DSA can provide complementary insights into the performance of DD models, and we have revised Appendix D accordingly to describe the analysis and its interpretation more clearly.

15. In response to the suggestion to comment on the outcomes of the NLB, the authors added a paragraph stating that users tended to focus on a small subset of datasets. This response, however, does not address the intent of the original question. It would be more informative to discuss the broader impact of the NLB on the modeling community, including how it influenced modeling practices across the field, which aspects of the NLB design were successful, and which aspects proved wrong. Such a discussion could naturally continue with an outlook on the impact the authors envision the CtDToolkit will have on the community.

Response: We thank the reviewer for this suggestion and agree that a broader discussion of the impact of the Neural Latents Benchmark (NLB) would strengthen the manuscript. One key lesson

from the NLB is that providing a single metric to optimize can lead to unintended consequences: when a measure becomes a target, it can cease to be a good measure.

In practice, this led to submissions that achieved strong benchmark performance without necessarily reflecting any modeling innovations aimed at improving interpretability or dynamical fidelity. For example, the original "winning" model was in fact an ensemble of multiple previously released models. This approach to NLB may have improved predictive accuracy but does not provide any additional insight into the underlying neural dynamics of computation.

We have revised the manuscript to incorporate this perspective, emphasizing that a central motivation for CtDToolkit is to move beyond single-metric evaluation toward a suite of complementary diagnostics that better capture different aspects of model quality. We have also expanded the discussion to reflect on which aspects of the NLB were successful, which revealed limitations, and how these lessons inform the design goals of CtDToolkit.

Minor comments

> *1. The text is often unclear about what is meant by "dynamics", whether it refers to the flow field or latents. The two are not equivalent, and at several places the text seems to conflate latents with the flow field.*

Response: We have fixed ambiguities in the usage of the word "dynamics" in the text.

> *2. The main text should state explicitly that DSA requires access to the true latents.*

Response: We have added this to the main text.

> *3. What is the definition of simple inputs (Fig. 2D)? How is the input simplicity measured?*

Response: We thank the reviewer for this question and agree that the notion of "input simplicity" was not clearly defined in the manuscript.

In the current work, input simplicity is a qualitative assessment based on properties that affect the ease with which intrinsic dynamics can be interpreted. In particular, we consider inputs to be simpler when they are (i) piecewise constant over time, and (ii) independent of the model's outputs. Piecewise constant inputs allow the system to be analyzed under effectively stationary conditions within each epoch, making standard dynamical systems tools (e.g., fixed point analysis) applicable. In contrast, continuously varying inputs (e.g., in PhaseCodedMemory or RandomTarget) introduce time-dependent forcing that complicates the interpretation of the underlying dynamics.

Additionally, when inputs depend on the model's outputs (as in the RandomTarget task), the system becomes intrinsically coupled to an external process (e.g., a controlled plant), making it difficult to disentangle the intrinsic dynamics of the network from the emergent dynamics of the combined system. This further increases the complexity of interpreting the inferred dynamics.

We have revised the manuscript (Section 2.3, where the task environments and their difficulty axes are introduced) to state explicitly that "input simplicity" refers to these properties (piecewise-constant, open-loop inputs), and that it is intended as a qualitative descriptor of how amenable a task is to standard dynamical analysis, rather than a formally defined scalar metric. We note that

the original panel referenced by the reviewer (Fig. 2D) has been removed for visual clarity, and the definition is now given in the main text.

> *4. The problem of determining the dimensionality of inputs is not mentioned.*

Response: For simplicity, in the current work we assume that the dimensionality of the external inputs is known. This allows us to focus on evaluating the inferred dynamics without introducing additional ambiguity from input dimensionality estimation. We have revised the manuscript to state this assumption explicitly (Section 3.3) and note that determining input dimensionality from data is an important related problem that we leave for future work.

> *5. Fig. 1G is confusing. If trajectory moves to the fixed point B, what happens as the input sequence continues? It seems this illustration works only if we consider a short trial with two input pulses, but it is unclear how we should understand the invented feature for a longer input sequence.*

Response: The intent of this figure was purely illustrative, to provide an intuitive example of what an "invented feature" might look like, rather than to depict a fully consistent dynamical system over extended input sequences. In particular, the schematic implicitly assumes a short input sequence (here, a single pair of input pulses) and is not meant to describe the system's behavior under prolonged or repeated inputs. We agree that this limitation was not clearly conveyed and may lead to ambiguity when interpreting the figure. We have revised the caption (Fig. 1G) to clarify that this is a simplified, conceptual illustration, and not a complete depiction of the system's dynamics under arbitrary input sequences.

For a concrete, empirical example of the kind of invented feature this schematic is meant to evoke, we point the reviewer to the per-trial level shifts in the 32D DD-NODE model shown in Fig. 1 of our response to comment 5 above (Fig. 5 technical issues): there, the DD model invents an extra dimension of across-trial latent variance that is not present in the task-trained latents. While Fig. 1G is a simplified within-trial schematic and does not itself depict this across-trial structure, both are instances of the broader phenomenon of feature invention that the schematic is intended to convey.

> *6. Ref. [38] does not use random initializations: all models are initialized at the minimum of model complexity.*

Response: We thank the reviewer for this clarification and agree that our description of Ref. [38] was imprecise. In that work, models are not selected based on arbitrary random initializations, but rather through a procedure that identifies the minimum-complexity regime in which solutions become consistent across runs. Our intent in citing this work was to highlight approaches that use consistency across independently trained models as a criterion for model selection, in contrast to the per-model diagnostics proposed here. We have revised the manuscript to more accurately describe this procedure and avoid implying that model selection in Ref. [38] is driven directly by random initialization.

7. "This suggests that not only is reconstruction insufficient for DD model selection, but relying on reconstruction alone may be actively misleading!" While the exclamation mark makes it sound like a revelation, this fact has long been established (see Ref. [38]). It would be appropriate to remove the exclamation mark and cite Ref. [38] in this sentence instead.

Response: We thank the reviewer for this suggestion and agree. We have removed the exclamation mark and revised the sentence to cite Ref. [38], acknowledging that the limitations of reconstruction-based model selection have been previously established.

8. "In Supp. Fig. S7, we show that DSA may be able to ignore such trivial non-linearities. However, it is still unclear whether these geometric differences can be safely ignored". Recent work shows that such geometric differences may indicate distinct task solutions and thus cannot be ignored (https://www.nature.com/articles/s42256-025-01127-2).

Response: We thank the reviewer for this important point and for bringing this recent work to our attention. We agree that geometric differences in the inferred dynamics may, in some cases, reflect genuinely distinct task solutions rather than trivial reparameterizations, and therefore should not be assumed to be safely ignorable.

Our intent in this statement was to highlight that certain nonlinear transformations may preserve aspects of the dynamical structure relevant to behavior, but we agree that this requires careful qualification. We have revised the manuscript to temper this claim and to acknowledge that geometric differences can correspond to meaningful differences in computation, as demonstrated in the cited work.

9. The description of models in Appendix is incomplete, many details are missing, making it impossible to understand. Since this paper is intended as a manual for a broad community of users, the authors should put a substantial effort in improving the methods sections in Appendix. Here are some examples of incomplete/confusing descriptions.

Response: We thank the reviewer for pointing out that several variables in the Appendix were not sufficiently defined. We have revised the appendix in detail, and provide explicit responses to the points listed below.

9.1. 3BFF task: what is x in the Loss function? What are x, U, B_h, b_z in Eqs. 9-13? Which of them are trainable parameters, inputs, and output? It should be briefly stated.

Response: In the 3BFF task, x_t in the loss function denotes the target output at time t, and $\hat{x}_t$ denotes the model-predicted output. In the NoisyGRU equations, x_t denotes the task input at time t; U_z, U_r, and U_h are trainable recurrent weight matrices; and b_z, b_r, and b_h are trainable bias terms for the corresponding GRU gates and candidate state update. We have revised the Appendix to explicitly label which quantities are task inputs, model outputs, hidden states, and trainable parameters.

9.2. How does GRU DD model map latents to firing rates?

Response: We agree that this should be stated explicitly. In the GRU DD model, latent states are mapped to firing rates through the observation model: the latent state is first linearly mapped to pre-rate activity, and this activity is then passed through the specified rectifying nonlinearity (e.g., exponential) to produce non-negative firing rates. We have added this description explicitly to the Appendix (Appendix D.3.1).

9.3. How b_h is different from b_x in Eq. 14?

Response: We agree that the distinction between b_h and b_x was not clear. In Eq. 14, b_h is the trainable bias term associated with the autonomous hidden-state update, whereas b_x is the trainable bias term associated with the input contribution. We have revised the text to make this distinction explicit and simplified the notation where appropriate to avoid confusion.

9.4. The text describes SLDS, but it is not included in the toolkit and no results are shown, so it should be removed from the text.

Response: We agree. Since SLDS is not currently included in the toolkit and no results using it are shown, we have removed this description from the manuscript. (We note that the S8 figure shows results for a non-switching LDS model; its title previously read "SLDS" in error, which we have corrected to "LDS.")

9.5. What is the time unit of the simulation? Do TT and DD models use the same time step?

Response: We agree that the simulation timescale should be stated more explicitly. The task-trained (TT) model, the neural simulator, and the data-driven (DD) models all operate on the same discretized trial bins: the simulator emits spikes on the bins of the TT latent trajectory, and the DD models are fit to those spikes without any rebinning or resampling. The TT and DD models therefore use an identical time step within a given dataset. The physical duration of a bin is task-dependent: for tasks tied to a physical process we set an explicit bin width (10 ms for RandomTarget, matching the MotorNet effector integration step; 20 ms for MultiTask; 2 ms for PhaseCodedMemory, a 0.8 s trial over 400 bins; and 1 ms for ChaoticDelayedMatching, so that the leaky-integration time constant $\tau = 30$ ms corresponds to the model's leak factor $\alpha = \mathrm{d}t/\tau = 1/30$), whereas for 3BFF the underlying dynamical system is scale-free and time is expressed in dimensionless bins with no physical unit. We have added a paragraph to the Task Environments appendix (Section B.1) stating this bin convention and the shared TT/DD time step explicitly.
