# Supplementary material for: Computation-through-Dynamics Toolkit: Simulated datasets and quality metrics for dynamical models of neural activity
Source: PLoS Comput Biol. 2026 Oct 15;22(10):e1014755. doi: 10.1371/journal.pcbi.1014755 (PMC13712701; doi:10.1371/journal.pcbi.1014755)
Supplement: Supplementary file 6 [file pcbi.1014755.s034.docx]

**Reviewer response for Computation-through-Dynamics Toolkit**

**Editor:**

Thank you for submitting your manuscript to PLOS Computational Biology. After careful consideration, we feel that it has merit but does not fully meet PLOS Computational Biology's publication criteria as it currently stands. Therefore, we invite you to submit a revised version of the manuscript that addresses the points raised during the review process.

Both the reviewers and I recognize that this work has the potential to be a great contribution to the field. However, they raise questions whether the metrics included in the framework are sufficient to provide a researcher with the intended insight, for example due to their linearity or need for ground truth data that wouldn't be available when carrying out model selection for experimental data. The reviewers make a variety of suggestions as to how to improve the usefulness of CtDB and better position it with regard to previous studies and related endeavours such as dynamic system reconstruction in computational physics.

**Response to the Editor**

We thank you and the reviewers for the thoughtful and constructive feedback. We have made substantial revisions to both the manuscript and the accompanying software to address the key points raised and to broaden the scope and utility of the framework.

1. **Renamed to better reflect intended use-case–** We have renamed the project from the *Computation-through-Dynamics Benchmark* to the *Computation-through-Dynamics Toolkit (CtDToolkit)* to better reflect its intended use-case and avoid confusion. The revised manuscript now emphasizes that CtDToolkit is a platform for both dataset generation and model development, rather than a benchmark where a leaderboard would be used to rank and compare models. Instead, we hope that CtDToolkit provides a platform for model developers to (1) identify architectural innovations that improve model performance generally across diverse datasets and (2) tailor models to perform effectively on datasets with specific structures or computational properties.
2. **Expanded dataset suite** – We have added a new dataset class featuring **cyclic dynamics**, reflecting an important category of recurrent dynamical systems observed in neural recordings that was not represented in the original submission. We also **formalize the process by which additional user-generated datasets will be added to the Toolkit**, further extending CtDToolkit’s long-term relevance to the field.
3. **New and extended metrics** – To improve interpretability and align with the broader dynamical systems identification literature, we have added several new metrics:
   - **MMD on feature distributions** (e.g., pairwise distance), requires ground-truth
   - **Maximal Lyapunov exponent**, quantifying local stability, requires ground-truth
   - **Nonlinear cycle-consistency**, which provides an estimate of simplicity, while relaxing the assumption of linear embeddings of latent activity. Does not require ground-truth
4. **New model class: ResLFADS** – We have incorporated a **Neural ODE–based LFADS variant (ResLFADS)** that allows lower-dimensional latent dynamics inference while retaining LFADS’ ability to infer external inputs.

Together, these revisions enhance **CtDToolkit’s role as a development platform for data-driven dynamical models**. The new datasets, metrics, and model classes expand its capacity to reveal architectural principles that generalize across diverse datasets, while also enabling targeted model development for specific experimental contexts. By combining theoretical grounding with practical flexibility, CtDToolkit provides researchers with a unified framework for generating datasets, evaluating models, and probing how inferred dynamics reflect underlying neural computations. We believe these updates substantially strengthen the manuscript and address the reviewers’ concerns regarding the Toolkit’s utility and relevance to the broader dynamical systems community.

**Reviewers' comments:**

**Responses**:

We first note that we have changed the title of this package from the *Computation-through-Dynamics Benchmark (CtDB)* to the *Computation-through-Dynamics Toolkit (CtDToolkit)*. This change reflects broad feedback that the term “Benchmark” could imply a leaderboard-style comparison of model performance, which is not our goal. Instead, CtDToolkit is intended as a **platform for the joint development of datasets and models for inferring dynamics**. This new name better captures the collaborative and extensible nature of the work, and is reflected in how we refer to the project throughout our responses to both reviewers.

**Reviewer #1**: The authors introduce a framework for evaluating methods and models for inferring computations implemented through dynamics from neural recordings. This includes a set of benchmark tasks and a set of measures to evaluate the performance of inference (“DD”) models trained on spiking data simulated by task-trained models. In general, this is a great and much needed contribution for the field.

We thank the reviewer for the kind words! We are glad that they agree that CtDToolkit addresses a pressing issue facing the data-driven dynamical modeling community. We also thank them for their suggestions, which we feel have improved the reach and impact of the toolkit to a wider community of dynamical modelers and neuroscientists.

I have a couple of suggestions for improvement however:

1) There is a meanwhile extensive literature on reconstructing dynamical systems (DS) from data in ML/AI and comp physics (partly reviewed in [1,2]), which I feel is highly relevant here, but is hardly mentioned (e.g. [1-10]). Although I’m aware that neuroscience may have its special needs, as the interest is often more in transient and non-autonomous (driven) dynamics, I feel there are a number of important insights, methodological developments, and evaluation metrics that will be highly useful for neuroscience as well. After all, authors in the DS field have thought a lot about what constitutes a valid DS reconstruction!

For instance, a successfully trained DD model should also produce long-term behavior that is compatible with recorded neural activity, e.g. express the same power spectrum, rather than drifting off into some completely unphysiological state or falling silent. A lot of work in the field of DS reconstruction emphasizes the correct reconstruction of long-term statistics, like the state space geometry/ topology, power spectrum, invariant measures, or invariant quantities like the Lyapunov spectrum [1-6]. Such properties are defining in DS theory and must be met if, for instance, the vector field or flow of an underlying system is to be correctly captured. In fact, in the field of DS reconstruction a lot of energy has been spent in devising training algorithms or loss functions that encourage the model to learn the correct long-term dynamics [6-10].

Also, many of these measures can be derived from data w/o knowledge of the ground truth system [1-6], thus potentially backing up the short (2-item) list of measures suitable for real data discussed by the authors. Specifically, the measures suggested by the authors are essentially linear (in contrast to most measures suggested in the DS lit.). This includes (contrary to the claim?) DSA, since in my understanding it’s still based on a linear DS fit followed by a linear/affine transformation? As the authors emphasize themselves, linear measures may miss a lot of important dynamical structure. Further, not every detail of the vector field may be relevant for computation, and measures that emphasize the correct topology [4] may often be more useful. Finally, there is the well-know problem of exponential divergence in chaotic systems, which makes R^2 scores (and other measures directly defined on the observed time series) only useful on short time scales (see e.g. [1]).

Thus, I would like to encourage the authors to discuss and potentially include other, nonlinear measures anchored in DS theory as well (such as provided in [1-10]).

We appreciate the reviewer's important insights about the broader literature of dynamical systems identification. In particular, the references have been an extremely helpful guide to performance metrics used in other domains of dynamical systems ID (DSID).

We agree that it is appropriate to include DSID metrics for DD model performance in CtDToolkit that complement the metrics included in the original submission. To this end, we have now implemented two additional metrics inspired by the DSID literature.

The first metric quantifies how well DD models capture order-invariant features of the underlying dynamical system via distribution matching distances (**MMD**) [1, 2]. We used this metric in the place of more common KL-based metrics (as in [3, 4]) because we require the ability to compare distributions that differ in dimension and we require invariance to rotations, translations, and uniform scalings.

The second metric allows users to estimate the long-term stability properties of the ground-truth and inferred systems (**Lyapunov Exponent estimation**) [5]. Importantly, computing MMD for DD models requires access to ground-truth. While the Lyapunov spectrum of DD models can be estimated without access to ground-truth, the estimate can only be verified if ground truth data are available.

We describe these methods' implementations in more detail in **Section 3.4.1** - Distribution Matching (MMD) and **Section 3.4.3** - Maximal Lyapunov Exponent. We also added a supplementary figure that compiles the performance of DD models included in **Fig. 3** with these new metrics.

We found that for the 3BFF dataset, DD models with optimal MMD and maximal Lyapunov exponents closest to ground-truth were also identified as “best-performing” using the CtDToolkit metrics that do not rely on ground-truth - Co-BPS and Cycle Consistency. These results provide alternative methods to evaluate DD models, and also provide confirmation that the previously-included CtDToolkit metrics guide model development in a manner that is corroborated by the broader DSID literature. We describe this result in more detail in **Section 4.2** and **Supplementary Figure 14 in Appendix Section E.4** (“Example Performance of DD Models on CtDToolkit Metrics”).

In addition, we include in the resubmission a previously published non-linear cycle-consistency metric which does not assume that the embedding of latent activity into neural activity is linear [6]. We demonstrate that the linear and non-linear cycle consistency metrics provide similar estimates on the 3BFF dataset in **Supplementary Figure 14** and provide implementation details in **Section E.4.**

2) I like the three benchmark task settings defined by the authors, I think there is a good rationale for them, but it is important to keep in mind that there are many, many other potential settings and tasks in neuroscience not reflected in this small set (e.g. producing longer sequences, behavioral planning, cross-modal transformations, tasks with dense sensory inputs etc.). So I think it is important to emphasize that these 3 tasks are just a starter: If models were developed in the field exclusively on this basis, they may “over-fit” this small task set and miss a lot of dynamics important for computation.

The most specific shortcomings in the suggested set of tasks that I can spot is the assumption that all computation rests on regular (=non-chaotic) dynamics, and mostly on fixed points. While I perfectly agree with the authors that there must be some reliable structure for computation, the nervous system, like any complex system with a lot of heterogeneous elements, is likely bound to be (highly) chaotic in many aspects [11,12]. Further, few neuroscientists I think would contest that oscillatory (cyclic) dynamics is highly relevant [13]. I therefore would suggest including one or two more benchmarks with cyclic and chaotic dynamics, e.g. based on the recent work by Nicolas Brunel [14].

We agree with the reviewer that we sampled only a small region of the total space of applicable datasets that reflect plausible neural computations. Obviously, it would not be plausible for us to span the full space ourselves. However, to broaden the appeal of CtDToolkit, we took two approaches in this revision: we added a new dataset with cyclic dynamics, and we clarified how additional task datasets may be modularly added by users.

We have added a new synthetic dataset to *CtDToolkit* called the **PhaseCodedMemory** task, which expands the toolkit along two important dimensions.

First, it introduces **cyclic dynamics**, inspired by recent work from Omri Barak’s lab on phase-coding as a mechanism for short-term memory in the hippocampus [7]. Unlike previous CtD datasets, which featured non-cyclic computations, this task generates strongly periodic neural trajectories that challenge models to infer and represent oscillatory structure.

Second, it represents a **distinct recording regime**: hippocampal activity characterized by **high-yield but low-rate** neurons (hundreds to thousands of units, mean firing ≈ 1 Hz) rather than the **lower-yield, higher-rate** intracortical recordings typical of MEA datasets. This contrast allows users to test how data-driven (DD) models generalize across regimes differing in both neural dimensionality and firing-rate sparsity.

We demonstrate this by training a TT-RNN to generate CA1-like activity and show that standard DD models (e.g., LFADS [8]) misinterpret the phase-coded computation as rate-coding, highlighting how *CtDToolkit* can reveal the limits of model interpretability under differing dynamical and recording conditions. We have included the above in the manuscript in the following sections: **Section 3.3** – Task Environments describes the PhaseCodedMemory task. **Section 4.1.** details how the simulated neural activity differs from other datasets.  **Section 4.1** “Key Observations” describes example DD model performance on the synthetic dataset. **Section B.1.2.** describes specific implementational details. **Supp. Fig. 9.** Shows the task schematic and performance of an example DD model.

Additionally, we have codified a **standard pipeline for users to add their own datasets** to those supported in CtDToolkit. To add a new dataset to CtDToolkit, contributors fork the repo, implement a task environment matching the Toolkit interface, train a task-trained model, and save the model plus a simulator. After validating behavioral performance and spiking realism, they register the dataset in gen_datasets.py (ideally, including a short “dataset card”, a Jupyter Notebook with task description, training details, and validation results). They then submit a pull request, which maintainers review for correctness, novelty, and scientific utility before merging into the official test set.

This new task-environment submission process (described in greater detail in **Appendix Section F.2**) will allow a greater diversity of datasets and encourage community engagement with the Toolkit.

3) I’m not quite sure what the authors mean by “held-out” data. I think it’s absolutely crucial to test on completely unseen data not included in training, like test trials/ sessions/ animals or left-out portions of the time series. However, if I understood correctly, the authors seem to suggest to merely test on additional spike trains from “unseen neurons” but generated by *same* latent states? This, in my mind, would not even constitute a proper “out-of-sample” test (but please forgive if I got this wrong), let alone out-of-domain.

We thank the reviewer for this important point. To clarify, we use the phrase “held-out” in **Section 4.1** to refer to held-out neuron sets: i.e., synthetic neurons whose activity is not provided as input to the DD model, but that the model is trained to reconstruct. Held-out neuron sets are an important control to ensure that models cannot achieve good reconstruction performance by simply autoencoding data through an identity function, passing-through the input spiking without learning any dynamics.

To put the reviewer’s mind at ease, we clarify that model performance is evaluated on traditional validation datasets, which are composed of trials held-out from training. In accordance with standard ML procedures, we always compute model performance metrics on samples not seen during training, ensuring that our results are not the trivial result of overfitting. We note that these validation trials are indeed from the same domain as trials from the training set, and as such don’t provide any insight into the ability of DD models to generalize to new behavior.

We appreciate the catch; we’ve now clarified the language on this point in **Section 4.1** (TT and Data simulation Pipeline), and in the caption for **Fig. 4.**

4) The authors demonstrate that LDS cannot learn the first two tasks (flip-flop, multi-task). In fact, LDS cannot solve non-linear tasks and exhibit many dynamical phenomena by well-known limitations of linear DS. For instance, linear DS cannot have multiple isolated fixed points or multi-stability more generally. It may be worthwhile stressing this, cos I feel that not everyone in neuroscience is aware of these quite fundamental limitations of LDS.
But my more important point here is: In contrast to the first two tasks, LDS seems to be on par with LFADS/GRU on the supposedly most complex third scenario (RandomTarget; Fig. 4), which is counter-intuitive. In fact, it suggests that RandomTarget may actually be the *easiest* of all three scenarios, if there is even a linear representation for it (on the side, “task complexity” is not really well defined in the paper in my mind). Perhaps this is because RandomTarget basically just requires regressing outputs onto inputs?

We agree that it is important to emphasize that the LDS model is incapable of capturing even the very simple activity of the 3BFF task. We note that the performance of LDS models on the RandomTarget dataset can be accounted for by these models being trained with provided external inputs. Because sensory inputs reflect the effects of previous muscle commands, RandomTarget is the only task with complex time-varying inputs that are informative about the state of the TT network itself. Thus, the difficulty of the RandomTarget dataset lies in disentangling the time-varying inputs to a model from the true intrinsic dynamics of the TT model.

To demonstrate the role of inputs in the performance of DD models trained on the RandomTarget dataset, we tested how well ground-truth RandomTarget task-trained latent activity can be predicted from inputs alone. We fit a linear model to predict the TT latent activity from the time-varying contextual and sensory inputs to see how well a model would perform it if it were a pure pass-through of its inputs without any intrinsic dynamics at all. This model predicted the ground-truth latent activity with an R2 value of 0.65, compared to a Rate R2 value of ~0.8 for the LDS DD models. The internal computations of the TT network are apparently highly inter-related with the time-varying input signal, making discovering the true computation extremely difficult.

In the resubmission **Section 4.1** (Key observations), we add emphasis to the difficulty in disentangling the time-varying inputs to the RandomTarget TT model from its intrinsic dynamics.

Minor points:
- I find the definition of computation based just on a mapping f:I→O a bit too narrow, at least if *external* inputs and *enacted* outputs are meant, since it would miss a lot of important computation performed during sleep, mental simulation or planning.

This is an important point that we have clarified in the revised manuscript. We summarize the change here: Our definition of “inputs” does not only include signals from the external world, but also signals sent from other brain areas. This definition could include inputs from mental simulations and planning routines (e.g., from internal models). Similarly, we consider “outputs” to include both outputs that result in a motor action and outputs transmitted to other brain areas that may not result in motor activity.

This more inclusive definition of inputs and outputs is described in **Section 2.1** (What do we mean by “neural computation”?), and added here for reference.

Importantly, these inputs and outputs do not necessarily relate to the external world: they can include communication with other brain areas (e.g., internal models, planning activity).

- In addition to GRU & LFADS, the inclusion of other popular DS reconstruction models may be considered (as e.g. discussed in [1-10]).

We agree that additional model architectures would improve the overall impact of the manuscript. We highlight that CtDToolkit also includes some recently developed NODE-based SAEs. NODE-based models can improve performance at dynamical inference over standard RNN modules by de-coupling the expressivity of the dynamics from their dimensionality, which for RNNs are both a product of the number of hidden units included in the model. NODE-based models represent a state-of-the-art model class for accurate inference of latent features from noisy neural activity [9,10], and thus represent an important area of future work in the field.

To provide additional models for users to experiment with, in the resubmission we provide a new variant of LFADS (called Residual LFADS, or ResLFADS) that replaces the standard GRU-based dynamics model with these NODEs. This addition to CtDToolkit will allow users to (for example) investigate how NODE-based LFADS influences the accuracy of inferred inputs.

We describe the ResLFADS implementation in **Section D.3.2**, where we also give instructions on how users can begin testing the effects of generator type on model performance.

- Fig. 5 reports an important & interesting obs., but it’s only shown for the 3BFF problem. Since it’s so important, I would like to encourage the authors to repeat this at least for the other tasks.

Good point. We now include two additional supplementary figures that demonstrate the same inverse relationship between reconstruction performance and simplicity (as quantified by Rate R2 + CO-BPS / State R2 + Cycle Consistency, respectively) for both MultiTask and RandomTarget datasets. The results of our DD models on both datasets replicate the main finding in Figure 5; increasing the model capacity can be a mixed blessing, improving reconstruction at the expense of invented features.

We include these results in **Supplementary Figures 13, 14, and 15**, and describe them in the main body in **Section 4.2.**

- The analysis in sect. 4 I find important, but a bit limited.

We have added additional analyses that replicate the findings from **Figure 5** on the two other canonical datasets (MultiTask + RandomTarget) and include additional metrics from DSID in the supplement. These additions provide additional confirmation of the included metrics, demonstrating their general utility at guiding DD model selection.

These changes were included as part of **Supplementary Figures 13, 14, and 15 and in Section 4.2.**

- Tasks and computational requirements are introduced in a very informal manner. It may help to include formal notation (as in a typical ML conference paper), e.g. what are the vector/number spaces inputs and observations come from, what is the precise function to be learned etc. In general, sometimes more detailed explanation would be helpful; e.g. I didn’t understand what is meant by “… the FP ring rotated into a set of output dimensions…” (p.8) and how that would solve the task.

We have made changes to improve the formality of the task description

These changes can be found in **Appendix Section B.1**, included in the “General Description” and “Loss Function” subsections for each task environment.

- Usually the term “embedding” in ML and DS refers to a function that projects the observed data into some latent or expanded space, but here it seems the term is used just the other way round (from latent space to data), which I find quite confusing.

To our knowledge this is a commonly-used way to refer to this relationship in the computation through dynamics field [11, 12]. The low-dimensional dynamics are considered to be “embedded” across many neurons. We considered alternative ways to express this idea, but none seemed less confusing than the current usage. We welcome alternative suggestions.

- p.5: The term “reconstruction” usually implies the true system has been well approximated, but here it seems the term is used differently from the DS lit.?

We chose the word “reconstruction” because it is the term that is most commonly used in the DD literature when quantifying the ability to re-generate input neural activity.

We welcome alternative suggestions, but we have clarified the use of the term for readers from the broader DSID field in **Section 2.2** – Reconstruction.

- p.10 top: Wouldn’t the rank of the projection matrix (relative to max. dim.) be a good metric already? Another way to test this may be to quantify how many of N models trained on the same task yield the same solution (retraining consistency).

While the rank of the projection matrix is useful in theory, in practice we find that these matrices almost always have a rank equal to the maximum possible rank of the projection matrix (i.e., for a NxM matrix, a rank of min(N,M)). The reason for this is that it is unlikely that the model finds a projection matrix with eigenvalues that are exactly zero. Even small deviations make these projections “full-rank” even if they have an “effective” null-space. Our cycle-consistency metric builds on previous work on the “effective rank” of projection matrices [13].

As Reviewer 2 noted, retraining consistency is an interesting potential metric for model evaluation. At this stage, however, we see CtDToolkit’s scope as focusing on metrics that can be computed directly from trained models, rather than those that depend on retraining or comparing multiple model instances. That said, CtDToolkit can still be used to illustrate the benefits of alternative training paradigms or model selection schemes—such as those based on retraining consistency.

We have added a paragraph to Discussion **Section 5** that discusses alternative training-paradigm-based methods to better ground CtDToolkit in the full space of techniques to improve DD model performance.

- Fig. 6E: I’m probably misinterpreting this, but it appears this is the same soln. as Fig. 6F, i.e. the same FP locations at the corners? And then just mathematically there must also be saddles in between the stable FP.

While the latent activity of the DD model in **Fig 6E** approximates the ground-truth latent activity (as shown in Panel D), the fixed points (green circles = stable FPs, red Xs = unstable FPs) are dramatically different. As we show in Panel B, this is due to the input inference module essentially taking over the role of the dynamics module and inferring external inputs that drive the latent state, rather than allowing the model to learn intrinsic dynamics that generate the data.

We have modified the text in **Section 4.3** and in the **Figure 6** caption to make the distinction between these two models more clearly.

**Response 1 References:**

1. Bińkowski, M., Sutherland, D. J., Arbel, M., & Gretton, A. (2018). *Demystifying MMD GANs*. arXiv preprint arXiv:1801.01401.
2. Unterthiner, T., van Steenkiste, S., Kurach, K., Marinier, R., Michalski, M., & Gelly, S. (2019). *FVD: A new metric for video generation*. In ICLR Workshop (DeepGenStruct).
3. Brenner, M., Hess, F., Koppe, G. &; Durstewitz, D.. (2024). Integrating Multimodal Data for Joint Generative Modeling of Complex Dynamics. *Proceedings of the 41st International Conference on Machine Learning*, in *Proceedings of Machine Learning Research* 235:4482-4516 Available from <https://proceedings.mlr.press/v235/brenner24a.html>.
4. Hemmer, C. J., & Durstewitz, D. (2025). True zero-shot inference of dynamical systems preserving long-term statistics. *arXiv preprint arXiv:2505.13192*.
5. Eisen, A. J., Ostrow, M., Chandra, S., Kozachkov, L., Miller, E. K., & Fiete, I. R. (2025). *Characterizing control between interacting subsystems with deep Jacobian estimation*. arXiv preprint arXiv:2507.01946.
6. Versteeg, C., Sedler, A. R., McCart, J. D., & Pandarinath, C. (2023). *Expressive dynamics models with nonlinear injective readouts enable reliable recovery of latent features from neural activity*. arXiv preprint arXiv:2309.06402.
7. Pals, M., Macke, J. H., & Barak, O. (2024). *Trained recurrent neural networks develop phase-locked limit cycles in a working memory task*. *PLoS Computational Biology, 20*(2): e1011852. https://doi.org/10.1371/journal.pcbi.1011852
8. Pandarinath, C., O’Shea, D. J., Collins, J., Jozefowicz, R., Stavisky, S. D., Kao, J. C., … & Sussillo, D. (2018). Inferring single-trial neural population dynamics using sequential auto-encoders. *Nature Methods, 15*(10), 805–815. https://doi.org/10.1038/s41592-018-0109-9
9. Kim, T. D., Luo, T. Z., Pillow, J. W., & Brody, C. D. (2021). Inferring latent dynamics underlying neural population activity via neural differential equations. In *Proceedings of the 38th International Conference on Machine Learning*(Vol. 139, pp. 5551–5561). PMLR.
10. Sedler, A. R., Versteeg, C., Pandarinath, C., et al. (2022). *A deep learning framework for inference of single-trial neural population activity from calcium imaging with sub-frame temporal resolution*. arXiv preprint arXiv:2212.03771.
11. Jazayeri, M., & Ostojic, S. (2021). Interpreting neural computations by examining intrinsic and embedding dimensionality. *Current Opinion in Neurobiology, 70*, 113-120. <https://doi.org/10.1016/j.conb.2021.08.002>
12. Langdon, C., & Engel, T. A. (2025). Latent circuit inference from heterogeneous neural responses during cognitive tasks. *Nature Neuroscience, 28*(3), 665–675. https://doi.org/10.1038/s41593-025-01869-7
13. Roy, O., & Vetterli, M. (2007). *The Effective Rank: A Measure of Effective Dimensionality*. In Proceedings of the 15th European Signal Processing Conference (EUSIPCO 2007), Poznań, Poland, September 3–7, 2007.

**Reviewer 1 References**
[1] <https://www.nature.com/articles/s41583-023-00740-7>
[2] <https://www.nature.com/articles/s42254-024-00688-2>
[3] <https://openreview.net/pdf?id=4t3ox9hj3z>
[4] <https://proceedings.mlr.press/v235/goring24a.html>
[5] <https://proceedings.mlr.press/v235/brenner24a.html>
[6]<https://pubs.aip.org/aip/cha/article/33/10/103107/2914133/Constraining-chaos-Enforcing-dynamical-invariants>
[7] <https://proceedings.mlr.press/v235/schiff24b.html>
[8] <https://proceedings.mlr.press/v202/hess23a.html>
[9]<https://proceedings.neurips.cc/paper_files/paper/2022/hash/495e55f361708bedbab5d81f92048dcd-Abstract-Conference.html>
[10]<https://proceedings.neurips.cc/paper_files/paper/2023/file/57d7e7e1593ad1ab6818c258fa5654ce-Paper-Conference.pdf>
[11] <https://www.science.org/doi/10.1126/science.274.5293.1724>
[12] <https://academic.oup.com/cercor/article-abstract/17/4/894/377757?redirectedFrom=fulltext>
[13] <https://www.science.org/doi/10.1126/science.1099745>
[14] <https://journals.aps.org/prx/abstract/10.1103/PhysRevX.13.011009>

Reviewer #2: The manuscript describes a model development and evaluation platform called the Computation-through-Dynamics Benchmark (CtDB). The platform offers task-trained (TT) models for generating synthetic neural activity and metrics for evaluating the quality of data-driven (DD) dynamical models fitted to neural activity data. The current implementation includes a few TT models, a few baseline DD models, and some evaluation metrics. The framework is extensible: users can contribute new TT models and metrics and use the platform to develop and tune their own DD models.

Dynamical systems modeling is an influential framework for interpreting neural computations in population recording data. The CtDB platform, like the earlier effort Neural Latents Benchmark (NLB), is founded on the premise that advancing this field requires standardized metrics and datasets for comparing the performance of different DD models. NLB relied solely on data reconstruction metrics—which do not guarantee accurate inference of latent dynamics—and used experimental datasets with unknown ground truth. CtDB aims to address these limitations by incorporating additional metrics and using synthetic datasets generated from task-trained artificial network models.

The central premise that standardized benchmarking is critical to advancing the field warrants more rigorous justification. In addition, most proposed metrics going beyond reconstruction require access to ground truth and are inapplicable for model selection in experimental data, which makes the utility of benchmarking on synthetic data using these metrics unclear. Finally, while discovery is expected to come from the flow field structure, current metrics—focused on latent trajectories—do not directly evaluate the accuracy of flow field reconstruction. These limitations diminish the overall impact of the proposed CtDB platform.

1) The value of standardized benchmarking in enabling scientific discovery needs to be articulated more clearly. What goals will such benchmarking achieve? In engineering applications, benchmarking aims to improve specific performance metrics that directly quantify a system’s value (e.g., the information transfer rate in BMI typing systems). In contrast, CtDB proposes benchmarking models intended to drive scientific discovery (dashed arrow in Fig. 1E), but there is no metric to directly assess a model’s potential for generating new insights. What is the expected outcome of CtDB? Is the CtDB effort expected to produce a model architecture that is universally optimal for scientific discovery across all brain regions, tasks, and research questions? If so, what is the evidence that such a model may exist? As a counterexample, a model purposefully designed to address a specific question, integrating existing knowledge about a particular brain region and task, may produce the deepest insights in that context, yet not generalize to other tasks or datasets. Evaluating such specialized models on unrelated tasks serves no meaningful purpose. If the goal is not to identify a universally optimal architecture, then what is the purpose of evaluating models on a wide range of standard tasks that are unrelated to the specific research question any given model is supposed to address?

We thank the reviewer for these thoughtful points. We agree that no single data-driven (DD) model will be universally optimal across all brain regions, tasks, and research questions. Indeed, it is for this reason that we intentionally omitted a public leaderboard. To further ensure there is no confusion about our intentions, we now have reframed our work from *Benchmark* to *Toolkit*. Rather than ranking models, the **CtDToolkit** aims to provide the field with shared *infrastructure*—standardized metrics, biologically grounded synthetic datasets, and flexible tools for generating custom datasets—that together enable more rigorous and interpretable use of DD models for scientific discovery.

Regarding the question about the role of CtDToolkit in the interpretation of neural computation: we consider that the use of DD models for neuroscience faces two primary challenges: (1) **accurate inference** of latent dynamics from recorded activity, and (2) **interpretation** of the inferred dynamics in computational terms. **CtDToolkit primarily targets the first challenge—developing methods that reliably recover dynamics from data.** Interpretation of those dynamics necessarily depends on the scientific context; for example, in our own work we interpret 3BFF via fixed-point analysis and RandomTarget via flow-field visualization of a specifically chosen sensorimotor plane. However, as we demonstrate in **Fig. 6**, interpretation is a moot point if the underlying dynamics are inferred inaccurately.

Regarding the question if we believe there to be a “universally optimal” model: we agree that a DD model’s ability to infer dynamics depends strongly on the structure of the underlying system—particularly on the form of its external inputs and the computations those inputs drive. Ideally, DD models will be validated using synthetic datasets designed to be similar to the biological datasets on which they will be fit. However, before CtDToolkit, the field lacked even much simpler tools for assessing DD model performance generally: (i) metrics that quantify key aspects of model performance relevant for inference (e.g., simplicity, input inference) and (ii) validation datasets that capture the fundamental properties of neural systems (e.g., structured inputs, functional computations). **CtDToolkit helps to address both gaps and, critically, takes the next step by providing a flexible platform for researchers to simulate synthetic datasets that reflect their prior knowledge about the systems they study.** This will enable DD model developers to fine-tune their models for the computational features and input structures relevant to their own experimental context, rather than assuming that strong performance on canonical datasets will generalize to the dynamics present in their specific domain.

In addition to changing the title of the package, we have revised the introduction to more clearly state the intended use-case for the Toolkit, both providing resources for general model development and for tailoring DD models for use in specific domains. This was included as an additional paragraph in **Section 2.2:** “Synthetic systems…”, major revisions in **Sections 3.1** “Key Motivation” and **Section 3.2** “Model developer’s guide to CtDToolkit”. Again, we thank the reviewer for this important feedback, which we hope will prevent users from being misled about the intended use-case of CtdToolkit.

2) Accuracy benchmarks for input inference are misleading. Input inference is an ill-posed problem: depending on an arbitrary choice of regularizers, neural responses can be explained entirely by inferred time-varying inputs or by recurrent dynamics (Fig. 1H). This problem has no principled solution, yet the benchmark gives the misleading impression that it may be solvable. Using Input R2 to select hyperparameters does yield accurate inferred inputs and dynamics, but only on synthetic data with known inputs. There is no guarantee that hyperparameters selected using Input R2 on synthetic data will yield correct results on experimental data. For example, hyperparameters tuned on synthetic data with sparse inputs (Fig. 6) will produce incorrect results when applied to experimental data with dense inputs (e.g., ramping input [1]). Thus, benchmarking input inference on synthetic data does not predict accuracy on experimental data—which is the actual objective—but instead creates the misleading impression that input inference problem may be solved.

We agree that input inference is an ill-posed problem and that accuracy benchmarks for input inference would be misleading. We apologize if this was not clear in the original submission. We contend that the first step toward addressing these challenges is to clearly demonstrate when and how they arise, and to provide metrics that can detect them when ground-truth information is available. This way, in the best case scenario (where ground truth is available), model developers and users can develop intuition for the variety of factors that affect the form that inferred inputs can take, their potential match or mismatch to known inputs in the test system, and how the family of solutions can affect the dynamical computations inferred by a model.

In the revised manuscript, we add additional emphasis to make clear we haven’t solved this fundamental issue, but we instead highlight how this failure mode can manifest—specifically, how models can reconstruct neural activity with minimal feature invention by offloading all of the dynamics to the inferred inputs (i.e., *dynamical misattribution*).

The Input R² metric shown in **Figure 6** was included to help illustrate this problem. As discussed in Section 4.3, there is no principled way to select optimal hyperparameters using metrics that do not rely on ground-truth data. We explicitly note that:

“However, we caution that the hyperparameters found to produce good Input R^2^ on this dataset are unlikely to work well on datasets with different properties; since Input R^2^ relies upon knowledge of the ground-truth, this presents a major unresolved challenge for the field of data-driven dynamical modeling. Multi-area recordings and perturbations will likely be essential for resolving the ambiguities associated with external input inference.”

In the longer term, multi-area recordings and/or perturbation studies may help make this problem more tractable. By providing empirical priors on the *structure* and *temporal evolution* of external inputs, such datasets can better constrain input inference and reduce ambiguities in the estimated dynamics.

3) While benchmarking on experimental data in NLB was limited by the lack of ground truth, the shift toward TT models in CtDB faces a complementary issue: the dynamics in these artificial models may bear little resemblance to brain activity during the same tasks, rendering the benchmarking effort irrelevant for understanding neural data. For example, working memory in TT models presented in CtDB relies on persistent activity supported by fixed point attractors, whereas population activity in the prefrontal cortex does not persist on single trials but instead alternates between intermittent epochs of high and low spiking rates [2]. Additionally, a major portion of neural variability is unrelated to computations that transform task inputs into task outputs [3,4]. Moreover, animals often use more complex strategies than directly mapping task inputs to outputs, integrating additional variables such as past choices, rewards, and motivation into their decision-making process [5]. Such strategies do not emerge in TT models unless the modeler explicitly engineers them by incorporating additional inputs, which implies prior knowledge of the computation and thereby defeats the purpose of inferring it from data.

We agree that the datasets in CtDToolkit span a range in their resemblance to biological neural data. Each dataset was chosen with a specific role in mind. For example, NBFF was included as a simple toy example, while MultiTask was included as a representative case at the frontier of our current ability to understand artificial dynamical computations. RandomTarget, by contrast, was included because it is a commonly used system that has been shown to produce high-level responses resembling those observed in motor cortex during reaching behavior [1]. We also introduced the PhaseCodedMemory task (from Pals et al. 2024 [2]), which extends the diversity of the toolkit by incorporating a model of working memory based on cyclic dynamics, further broadening the range of datasets represented in CtDToolkit.

Of course, it would be impossible for us to release enough datasets to fully capture the diversity of neural dynamics found in the brain. Moreover, including *too many* datasets could dilute the advantages of a shared evaluation framework by dispersing community effort across an unmanageably broad range of tasks. Instead, we have curated a small set of datasets that each play a distinct and valuable role in validating data-driven (DD) models. To enable expansion beyond the Canonical datasets available at release, we now provide a formalized pipeline through which community members can contribute their own task environments and simulated datasets for public use (**Appendix Section F.2**). This approach encourages broader participation and helps address the reviewer’s concern about representational breadth.

We hope that these clarifications and additions underscore the value of CtDToolkit, and we look forward to the community using and extending it as a shared resource for building and testing DD models.

4) The discovery of computation is expected to come from the flow field structure (Fig. 1E), but evaluation metrics are based solely on latents, not on the flow field. Producing "good" latents on the held-out data does not guarantee that the flow field does not contain invented features. High-dimensional models can develop invented features in the flow field (especially in the undersampled regions of the state space), yet infer reasonable latents, since the inference uses both the model and the data. Although DSA and fixed-point search are offered as additional visualization tools, they require ground truth for evaluating whether the inferred dynamics are accurate.

We agree with the reviewer that accurate latent reconstruction does not guarantee faithful recovery of the underlying flow field, and that high-dimensional models can produce “invented” features in undersampled regions of state space. We do not claim to have fully solved this fundamental challenge or to provide any guarantee of ground-truth recovery. Rather, our goal is to develop tools that help researchers identify when common issues are likely to arise and provide some quantifiable metrics of model performance. While these evaluations cannot guarantee correctness, they can indicate *relative reliability*: models that perform better across the CtDToolkit metrics are, in a meaningful sense, less wrong, even if they are not fully right.

To provide an additional means of assessing model performance, we have introduced a new metric – the Maximal Lyapunov Exponent (MLE) – which quantifies the long-term stability of TT and DD models using the inferred flow field directly (i.e., an evaluation based on the dynamics themselves rather than the latents). Although the Lyapunov spectrum can be estimated without access to ground-truth data, its accuracy cannot be verified without corresponding ground-truth values for comparison. We demonstrate this new metric in **Supplementary Figure 15**.

5) The state R2 and cycle consistency metrics are linear, which is problematic. Any nonlinear change of variables in the latent dynamical system and its embedding yields an equivalent model. Linear state R2 only works because the embedding g(x) is fixed to be linear, eliminating nonlinear changes of variables. However, there is no reason to prefer linear embeddings. Nonlinear embeddings enable capturing dynamics on the manifold with the minimal number of latent dimensions [6]. Either modeling choice may be useful depending on the scientific question.

We agree that the linear cycle-consistency metrics rely on the assumption of a linear embedding from latent activity to neural activity. We also agree that non-linear readouts can improve the interpretability of inferred dynamics; previous work from our lab and others has shown that relaxing the linearity assumption can substantially reduce the dimensionality required to capture a system’s activity [3,4].

To address this limitation, we now include an alternative measure – nonlinear cycle-consistency – that does not assume a specific mapping from latent activity to predicted firing rates. This metric (introduced previously in [3]) is computed by training an multi-layer feed-forward neural network to map predicted firing rates back to the inferred latent activity, and then testing the robustness of this inverse mapping to different levels of noise injected into the firing rates. Intuitively, when a readout compresses large distances in latent space into small distances in firing-rate space (i.e., is nearly non-injective), small perturbations in the rates will produce large reconstruction errors in the latent space. We demonstrate that the linear and nonlinear cycle-consistency metrics yield similar estimates on the 3BFF dataset (**Supplementary Figure 14**). We provide implementation details in **Section E.4**.

Relatedly, the CtDToolkit also enables users to explore the effects of nonlinear embeddings directly through dataset simulation. As described in **Appendix Section C**, the embed_dict object in the Simulator allows users to select among several embedding functions – Sigmoidal, SoftPlus, and the canonical LinExp – or to define their own. To make this functionality more apparent, we have updated the main text to explicitly note that CtDToolkit supports simulation of datasets with nonlinear embedding functions. This capability allows users to systematically test how deviations from the canonical linear-exponential embedding influence the performance and interpretability of DD models, addressing the reviewer’s broader point about the importance of nonlinear mappings.

6) Injective mappings g(x) do not generally prevent learning of spurious features in the flow field, which arise in expressive models due to overfitting to noise [7]. Spurious features do not only result form excess dimensionality in the latent DD model, but arise also when the latent dimensionality matches the ground truth. The point that good data reconstruction does not ensure that a DD model identified the correct features was already noted in [7], which is not cited here despite raising this concerns prior to Ref. 46, 55. Moreover, [7] proposed a model selection approach based on cross-validation of model features instead of reconstruction metrics, without requiring ground truth, contrary to the statement here that all approaches need ground truth.
We agree that injective mappings cannot, by themselves, guarantee that spurious dynamical features are absent. However, they serve an important role in promoting interpretability: by enforcing a one-to-one relationship between latent activity and predicted firing rates, injective readouts ensure that any change in the latents must produce a measurable change in model output, discouraging (but not fully preventing) models from encoding irrelevant or redundant structure in their latent spaces and helps limit the extent of feature invention during training.

We thank the reviewer for drawing our attention to this prior work, which we have now cited in the revised manuscript. The approach proposed there (training multiple models and selecting those that converge to similar inferred features) offers a valuable framework for model selection without requiring ground truth. While such training-phase procedures can be powerful tools for identifying robust models, CtDToolkit is designed primarily to provide *post-hoc evaluation metrics* that can be applied to models after training, independent of the specific training protocol used. In this sense, the two efforts are complementary: methods like those in the reference can help guide model discovery during training, whereas CtDToolkit provides a platform for demonstrating these improvements relative to alternative training approaches.

To better acknowledge these complementary approaches, we have added the following paragraph to the Discussion (**Section 5**):

“There are also alternative approaches for addressing spurious feature learning focus on training-time model selection procedures, such as cross-validation of model features rather than reconstruction metrics, or splitting datasets to train multiple models and selecting those that converge on similar solutions \cite{Genkin2020-ow}. These training-phase interventions differ from the evaluation metrics provided in CtDToolkit, which are designed for post-hoc assessment of previously trained models. We expect models trained using such approaches to perform better on the post-hoc metrics included in CtDToolkit, providing an additional use case that extends beyond the Toolkit’s current scope.”

7) Scientific insight is expected to come from the flow field (Fig. 1E), but it is unclear how this insight will be derived in a more complex random target task, using any DD model. It would be helpful to clarify what is the ground truth flow field here. While this task is described as the most difficult, it needs to be clarified how it fits within the CtDB framework, where the ultimate goal is the dashed arrow in Fig. 1E. If it is fundamentally impossible to derive this insight even in TT model, it undermines the premise of fitting DD models to understand computation.

We thank the reviewer for this insightful comment. To clarify, the field of data-driven (DD) modeling – and by extension, the purpose of the CtDToolkit – has two interdependent goals. The final goal is to use the inferred flow fields to derive interpretable accounts of neural computation (the dashed arrow in Fig. 1E). However, achieving this goal first requires progress toward a critical intermediate goal: ensuring that the inferred flow fields accurately reflect the true underlying dynamics. Without confidence in the accuracy of these inferred dynamics, any interpretation of the computation would be unreliable.

In this context, the RandomTarget task plays a specific role within the CtDToolkit framework. It is intentionally difficult because it represents a situation where the ground-truth flow field of the teacher–target (TT) model alone is insufficient to fully explain the computation, as the TT model’s dynamics are coupled to those of the simulated motor plant. This makes the overall computation inherently distributed across interacting systems. We state this explicitly when introducing RandomTarget in **Section 3.3:**

“Given the combination of task complexity, time-varying inputs, and lack of prior understanding of the computational structures involved, RandomTarget is perhaps the most challenging dataset currently included in CtDToolkit. As biological circuits also receive complex, time-varying inputs that likely reflect coupled dynamics, RandomTarget is also arguably most similar to datasets that might be collected experimentally.”

We agree that we still don’t fully understand how the TT model performing the RandomTarget task implements its computation. Nonetheless, examining the dynamics of the coupled TT + arm system reveals some promising, interpretable structure: activity within one sensorimotor plane of the TT model behaves like a feedback controller, transforming sensory inputs into motor outputs that correct goal-relevant perturbations (Fig. 3I). This offers an illustrative, if partial, example of how flow-field analysis can provide insight into the computations embedded in a system’s dynamics, provided the system boundaries are defined carefully.

Finally, we note that the value of the RandomTarget dataset does not depend on a complete understanding of the TT network itself. Accurately inferring the features of the data-generating system – the intermediate goal – is a prerequisite for deriving scientific insights from those inferred dynamics – the final goal. RandomTarget thus serves as a synthetic test case that captures many of the challenges we expect from biological datasets: (1) complex, time-varying inputs, (2) coupling to other dynamical systems, and (3) goal-directed behavior. Even without full interpretability of the TT network’s computation, the task provides a valuable benchmark for assessing whether DD models can accurately recover the system’s underlying activity and dynamics.

8) Describing the embedding g(x) as "implementation in a neural circuit" is misleading. The arbitrary (in this case linear) mapping g(x) bears no relationship to how the dynamics arise from the connectivity in a neural circuit. A more suitable term would be "implemented in neural activity", removing all references to circuits

We agree with this suggested change in terminology, and have made the appropriate revisions in the final manuscript. The phrase “neural circuit” is only used in reference to actual biological neurons, rather than an abstraction of an interconnected network.

9) It is important to discuss the outcomes of NLB and its relationship to CtDB. Since this effort builds on NLB, it is critical to reflect on its specific successes and failures, as well as the broader challenges of benchmarking in neuroscience.

We have added a section in the discussion that expands on the role of NLB in the creation of CtDToolkit, and how its adoption has influenced the choices we made while designing and building this benchmark. This can be found in **Section 5**, and is copied here:

Based on our experience with the Neural Latents Benchmark (NLB) \cite{pei_neural_2022}, we observed that users tended to focus on a small subset of "workhorse" datasets rather than engaging broadly with the full range of available benchmarks. This suggested that including an extensive collection of diverse datasets could potentially dilute efforts across many standards. Here, we prioritize a smaller, curated set of well-validated datasets to help the field consolidate around common standards of model evaluation, accepting that this approach may limit applicability to neural recordings with substantially different characteristics or computational demands. We hope that researchers will continue to expand the collection of datasets by submitting their tasks and models to CtDToolkit following the procedure described in Appendix F.2.

**Response 2 References:**

1. Sussillo, D., Churchland, M., Kaufman, M. T., & Shenoy, K. V. (2015). *A neural network that finds a naturalistic solution for the production of muscle activity.* *Nature Neuroscience, 18*(7), 1025–1033. https://doi.org/10.1038/nn.4042
2. Pals, M., Macke, J. H., & Barak, O. (2024). *Trained recurrent neural networks develop phase-locked limit cycles in a working memory task*. *PLoS Computational Biology, 20*(2): e1011852. https://doi.org/10.1371/journal.pcbi.1011852
3. Versteeg, C., Sedler, A. R., McCart, J. D., & Pandarinath, C. (2023). *Expressive dynamics models with nonlinear injective readouts enable reliable recovery of latent features from neural activity*. arXiv preprint arXiv:2309.06402.
4. Jazayeri, M., & Ostojic, S. (2021). Interpreting neural computations by examining intrinsic and embedding dimensionality. *Current Opinion in Neurobiology, 70*, 113-120. <https://doi.org/10.1016/j.conb.2021.08.002>

**Reviewer 2 References:**
[1] <https://www.nature.com/articles/s41593-021-00840-6>
[2] <https://www.nature.com/articles/s41586-024-08139-9>
[3] <https://www.nature.com/articles/s41593-019-0502-4>
[4] <https://www.science.org/doi/10.1126/science.aav7893>
[5] <https://www.nature.com/articles/nature25510>
[6] <https://www.biorxiv.org/content/10.1101/2023.07.22.550183v1>
[7] <https://www.nature.com/articles/s42256-020-00242-6>
