# Supplementary material for: Computation-through-Dynamics Toolkit: Simulated datasets and quality metrics for dynamical models of neural activity
Source: PLoS Comput Biol. 2026 Oct 15;22(10):e1014755. doi: 10.1371/journal.pcbi.1014755 (PMC13712701; doi:10.1371/journal.pcbi.1014755)
Supplement: Peer review history

# Peer review: Decision Letter 0

**DOI:** 10.1371/journal.pcbi.1014755.r001

**Authors:** Abigail Morrison, Marieke Karlijn van Vugt

6 Jul 2025

PCOMPBIOL-D-25-00520

Computation-through-Dynamics Benchmark: Simulated datasets and quality metrics for dynamical models of neural activity

PLOS Computational Biology

Dear Dr. Versteeg,

Thank you for submitting your manuscript to PLOS Computational Biology. After careful consideration, we feel that it has merit but does not fully meet PLOS Computational Biology's publication criteria as it currently stands. Therefore, we invite you to submit a revised version of the manuscript that addresses the points raised during the review process.

Both the reviewers and I recognize that this work has the potential to be a great contribution to the field. However, they raise questions whether the metrics included in the framework are sufficient to provide a researcher with the intended insight, for example due to their linearity or need for ground truth data that wouldn't be available when carrying out model selection for experimental data. The reviewers make a variety of suggestions as to how to improve the usefulness of CtDB and better position it with regard to previous studies and related endeavours such as dynamic system reconstruction in computational physics.

Please submit your revised manuscript within 60 days Sep 05 2025 11:59PM. If you will need more time than this to complete your revisions, please reply to this message or contact the journal office at ploscompbiol@plos.org. When you're ready to submit your revision, log on to https://www.editorialmanager.com/pcompbiol/ and select the 'Submissions Needing Revision' folder to locate your manuscript file.

Please include the following items when submitting your revised manuscript:

* A rebuttal letter that responds to each point raised by the editor and reviewer(s). You should upload this letter as a separate file labeled 'Response to Reviewers'. This file does not need to include responses to formatting updates and technical items listed in the 'Journal Requirements' section below.

* A marked-up copy of your manuscript that highlights changes made to the original version. You should upload this as a separate file labeled 'Revised Manuscript with Track Changes'.

* An unmarked version of your revised paper without tracked changes. You should upload this as a separate file labeled 'Manuscript'.

If you would like to make changes to your financial disclosure, competing interests statement, or data availability statement, please make these updates within the submission form at the time of resubmission. Guidelines for resubmitting your figure files are available below the reviewer comments at the end of this letter

We look forward to receiving your revised manuscript.

Kind regards,

Abigail Morrison

Academic Editor

PLOS Computational Biology

Marieke van Vugt

Section Editor

PLOS Computational Biology

**Journal Requirements:**

1) We ask that a manuscript source file is provided at Revision. Please upload your manuscript file as a .doc, .docx, .rtf or .tex. If you are providing a .tex file, please upload it under the item type u2018LaTeX Source Fileu2019 and leave your .pdf version as the item type u2018Manuscriptu2019.

2) Please provide an Author Summary. This should appear in your manuscript between the Abstract (if applicable) and the Introduction, and should be 150-200 words long. The aim should be to make your findings accessible to a wide audience that includes both scientists and non-scientists. Sample summaries can be found on our website under Submission Guidelines:

https://journals.plos.org/ploscompbiol/s/submission-guidelines#loc-parts-of-a-submission

3) Your manuscript is missing the following sections: Introduction, and Methods.  Please ensure all required sections are present and in the correct order. Make sure section heading levels are clearly indicated in the manuscript text, and limit sub-sections to 3 heading levels. An outline of the required sections can be consulted in our submission guidelines here:

https://journals.plos.org/ploscompbiol/s/submission-guidelines#loc-parts-of-a-submission

4) Please upload all main figures as separate Figure files in .tif or .eps format. For more information about how to convert and format your figure files please see our guidelines:

https://journals.plos.org/ploscompbiol/s/figures

5) We notice that your supplementary Figures, Tables, and information are included in the manuscript file. Please remove them and upload them with the file type 'Supporting Information'. Please ensure that each Supporting Information file has a legend listed in the manuscript after the references list.

Note: Please cite and label the supplementary tables and figures as “S1 Table” and “S2 Table,” "S1 Figure", S2 Figure" and so forth.

6) Please ensure that the funders and grant numbers match between the Financial Disclosure field and the Funding Information tab in your submission form. Note that the funders must be provided in the same order in both places as well. Currently "the Centre Interdisciplinaire de Recherche sur le Cerveau et l’Apprentissage" is missing from the Funding Information tab.                                          ".

**Reviewers' comments:**

Reviewer's Responses to Questions

Reviewer #1: The authors introduce a framework for evaluating methods and models for inferring computations implemented through dynamics from neural recordings. This includes a set of benchmark tasks and a set of measures to evaluate the performance of inference (“DD”) models trained on spiking data simulated by task-trained models. In general, this is a great and much needed contribution for the field.

I have a couple of suggestions for improvement however:

1) There is a meanwhile extensive literature on reconstructing dynamical systems (DS) from data in ML/AI and comp physics (partly reviewed in [1,2]), which I feel is highly relevant here, but is hardly mentioned (e.g. [1-10]). Although I’m aware that neuroscience may have its special needs, as the interest is often more in transient and non-autonomous (driven) dynamics, I feel there are a number of important insights, methodological developments, and evaluation metrics that will be highly useful for neuroscience as well. After all, authors in the DS field have thought a lot about what constitutes a valid DS reconstruction!

For instance, a successfully trained DD model should also produce long-term behavior that is compatible with recorded neural activity, e.g. express the same power spectrum, rather than drifting off into some completely unphysiological state or falling silent. A lot of work in the field of DS reconstruction emphasizes the correct reconstruction of long-term statistics, like the state space geometry/ topology, power spectrum, invariant measures, or invariant quantities like the Lyapunov spectrum [1-6]. Such properties are defining in DS theory and must be met if, for instance, the vector field or flow of an underlying system is to be correctly captured. In fact, in the field of DS reconstruction a lot of energy has been spent in devising training algorithms or loss functions that encourage the model to learn the correct long-term dynamics [6-10].

Also, many of these measures can be derived from data w/o knowledge of the ground truth system [1-6], thus potentially backing up the short (2-item) list of measures suitable for real data discussed by the authors. Specifically, the measures suggested by the authors are essentially linear (in contrast to most measures suggested in the DS lit.). This includes (contrary to the claim?) DSA, since in my understanding it’s still based on a linear DS fit followed by a linear/affine transformation? As the authors emphasize themselves, linear measures may miss a lot of important dynamical structure. Further, not every detail of the vector field may be relevant for computation, and measures that emphasize the correct topology [4] may often be more useful. Finally, there is the well-know problem of exponential divergence in chaotic systems, which makes R^2 scores (and other measures directly defined on the observed time series) only useful on short time scales (see e.g. [1]).

Thus, I would like to encourage the authors to discuss and potentially include other, nonlinear measures anchored in DS theory as well (such as provided in [1-10]).

2) I like the three benchmark task settings defined by the authors, I think there is a good rationale for them, but it is important to keep in mind that there are many, many other potential settings and tasks in neuroscience not reflected in this small set (e.g. producing longer sequences, behavioral planning, cross-modal transformations, tasks with dense sensory inputs etc.). So I think it is important to emphasize that these 3 tasks are just a starter: If models were developed in the field exclusively on this basis, they may “over-fit” this small task set and miss a lot of dynamics important for computation.

The most specific shortcomings in the suggested set of tasks that I can spot is the assumption that all computation rests on regular (=non-chaotic) dynamics, and mostly on fixed points. While I perfectly agree with the authors that there must be some reliable structure for computation, the nervous system, like any complex system with a lot of heterogeneous elements, is likely bound to be (highly) chaotic in many aspects [11,12]. Further, few neuroscientists I think would contest that oscillatory (cyclic) dynamics is highly relevant [13]. I therefore would suggest including one or two more benchmarks with cyclic and chaotic dynamics, e.g. based on the recent work by Nicolas Brunel [14].

3) I’m not quite sure what the authors mean by “held-out” data. I think it’s absolutely crucial to test on completely unseen data not included in training, like test trials/ sessions/ animals or left-out portions of the time series. However, if I understood correctly, the authors seem to suggest to merely test on additional spike trains from “unseen neurons” but generated by *same* latent states? This, in my mind, would not even constitute a proper “out-of-sample” test (but please forgive if I got this wrong), let alone out-of-domain.

4) The authors demonstrate that LDS cannot learn the first two tasks (flip-flop, multi-task). In fact, LDS cannot solve non-linear tasks and exhibit many dynamical phenomena by well-known limitations of linear DS. For instance, linear DS cannot have multiple isolated fixed points or multi-stability more generally. It may be worthwhile stressing this, cos I feel that not everyone in neuroscience is aware of these quite fundamental limitations of LDS.

But my more important point here is: In contrast to the first two tasks, LDS seems to be on par with LFADS/GRU on the supposedly most complex third scenario (RandomTarget; Fig. 4), which is counter-intuitive. In fact, it suggests that RandomTarget may actually be the *easiest* of all three scenarios, if there is even a linear representation for it (on the side, “task complexity” is not really well defined in the paper in my mind). Perhaps this is because RandomTarget basically just requires regressing outputs onto inputs?

Minor points:

- I find the definition of computation based just on a mapping f:I→O a bit too narrow, at least if *external* inputs and *enacted* outputs are meant, since it would miss a lot of important computation performed during sleep, mental simulation or planning.

- In addition to GRU & LFADS, the inclusion of other popular DS reconstruction models may be considered (as e.g. discussed in [1-10]).

- Fig. 5 reports an important & interesting obs., but it’s only shown for the 3BFF problem. Since it’s so important, I would like to encourage the authors to repeat this at least for the other tasks.

- The analysis in sect. 4 I find important, but a bit limited.

- Tasks and computational requirements are introduced in a very informal manner. It may help to include formal notation (as in a typical ML conference paper), e.g. what are the vector/number spaces inputs and observations come from, what is the precise function to be learned etc. In general, sometimes more detailed explanation would be helpful; e.g. I didn’t understand what is meant by “… the FP ring rotated into a set of output dimensions…” (p.8) and how that would solve the task.

- Usually the term “embedding” in ML and DS refers to a function that projects the observed data into some latent or expanded space, but here it seems the term is used just the other way round (from latent space to data), which I find quite confusing.

- p.5: The term “reconstruction” usually implies the true system has been well approximated, but here it seems the term is used differently from the DS lit.?

- p.10 top: Wouldn’t the rank of the projection matrix (relative to max. dim.) be a good metric already? Another way to test this may be to quantify how many of N models trained on the same task yield the same solution (retraining consistency).

- Fig. 6E: I’m probably misinterpreting this, but it appears this is the same soln. as Fig. 6F, i.e. the same FP locations at the corners? And then just mathematically there must also be saddles in between the stable FP.

References

[1] https://www.nature.com/articles/s41583-023-00740-7

[2] https://www.nature.com/articles/s42254-024-00688-2

[3] https://openreview.net/pdf?id=4t3ox9hj3z

[4] https://proceedings.mlr.press/v235/goring24a.html

[5] https://proceedings.mlr.press/v235/brenner24a.html

[6] https://pubs.aip.org/aip/cha/article/33/10/103107/2914133/Constraining-chaos-Enforcing-dynamical-invariants

[7] https://proceedings.mlr.press/v235/schiff24b.html

[8] https://proceedings.mlr.press/v202/hess23a.html

[9] https://proceedings.neurips.cc/paper_files/paper/2022/hash/495e55f361708bedbab5d81f92048dcd-Abstract-Conference.html

[10] https://proceedings.neurips.cc/paper_files/paper/2023/file/57d7e7e1593ad1ab6818c258fa5654ce-Paper-Conference.pdf

[11] https://www.science.org/doi/10.1126/science.274.5293.1724

[12] https://academic.oup.com/cercor/article-abstract/17/4/894/377757?redirectedFrom=fulltext

[13] https://www.science.org/doi/10.1126/science.1099745

[14] https://journals.aps.org/prx/abstract/10.1103/PhysRevX.13.011009

Reviewer #2: The manuscript describes a model development and evaluation platform called the Computation-through-Dynamics Benchmark (CtDB). The platform offers task-trained (TT) models for generating synthetic neural activity and metrics for evaluating the quality of data-driven (DD) dynamical models fitted to neural activity data. The current implementation includes a few TT models, a few baseline DD models, and some evaluation metrics. The framework is extensible: users can contribute new TT models and metrics and use the platform to develop and tune their own DD models.

Dynamical systems modeling is an influential framework for interpreting neural computations in population recording data. The CtDB platform, like the earlier effort Neural Latents Benchmark (NLB), is founded on the premise that advancing this field requires standardized metrics and datasets for comparing the performance of different DD models. NLB relied solely on data reconstruction metrics—which do not guarantee accurate inference of latent dynamics—and used experimental datasets with unknown ground truth. CtDB aims to address these limitations by incorporating additional metrics and using synthetic datasets generated from task-trained artificial network models.

The central premise that standardized benchmarking is critical to advancing the field warrants more rigorous justification. In addition, most proposed metrics going beyond reconstruction require access to ground truth and are inapplicable for model selection in experimental data, which makes the utility of benchmarking on synthetic data using these metrics unclear. Finally, while discovery is expected to come from the flow field structure, current metrics—focused on latent trajectories—do not directly evaluate the accuracy of flow field reconstruction. These limitations diminish the overall impact of the proposed CtDB platform.

1) The value of standardized benchmarking in enabling scientific discovery needs to be articulated more clearly. What goals will such benchmarking achieve? In engineering applications, benchmarking aims to improve specific performance metrics that directly quantify a system’s value (e.g., the information transfer rate in BMI typing systems). In contrast, CtDB proposes benchmarking models intended to drive scientific discovery (dashed arrow in Fig. 1E), but there is no metric to directly assess a model’s potential for generating new insights. What is the expected outcome of CtDB? Is the CtDB effort expected to produce a model architecture that is universally optimal for scientific discovery across all brain regions, tasks, and research questions? If so, what is the evidence that such a model may exist? As a counterexample, a model purposefully designed to address a specific question, integrating existing knowledge about a particular brain region and task, may produce the deepest insights in that context, yet not generalize to other tasks or datasets. Evaluating such specialized models on unrelated tasks serves no meaningful purpose. If the goal is not to identify a universally optimal architecture, then what is the purpose of evaluating models on a wide range of standard tasks that are unrelated to the specific research question any given model is supposed to address?

2) Accuracy benchmarks for input inference are misleading. Input inference is an ill-posed problem: depending on an arbitrary choice of regularizers, neural responses can be explained entirely by inferred time-varying inputs or by recurrent dynamics (Fig. 1H). This problem has no principled solution, yet the benchmark gives the misleading impression that it may be solvable. Using Input R2 to select hyperparameters does yield accurate inferred inputs and dynamics, but only on synthetic data with known inputs. There is no guarantee that hyperparameters selected using Input R2 on synthetic data will yield correct results on experimental data. For example, hyperparameters tuned on synthetic data with sparse inputs (Fig. 6) will produce incorrect results when applied to experimental data with dense inputs (e.g., ramping input [1]). Thus, benchmarking input inference on synthetic data does not predict accuracy on experimental data—which is the actual objective—but instead creates the misleading impression that input inference problem may be solved.

3) While benchmarking on experimental data in NLB was limited by the lack of ground truth, the shift toward TT models in CtDB faces a complementary issue: the dynamics in these artificial models may bear little resemblance to brain activity during the same tasks, rendering the benchmarking effort irrelevant for understanding neural data. For example, working memory in TT models presented in CtDB relies on persistent activity supported by fixed point attractors, whereas population activity in the prefrontal cortex does not persist on single trials but instead alternates between intermittent epochs of high and low spiking rates [2]. Additionally, a major portion of neural variability is unrelated to computations that transform task inputs into task outputs [3,4]. Moreover, animals often use more complex strategies than directly mapping task inputs to outputs, integrating additional variables such as past choices, rewards, and motivation into their decision-making process [5]. Such strategies do not emerge in TT models unless the modeler explicitly engineers them by incorporating additional inputs, which implies prior knowledge of the computation and thereby defeats the purpose of inferring it from data.

4) The discovery of computation is expected to come from the flow field structure (Fig. 1E), but evaluation metrics are based solely on latents, not on the flow field. Producing "good" latents on the held-out data does not guarantee that the flow field does not contain invented features. High-dimensional models can develop invented features in the flow field (especially in the undersampled regions of the state space), yet infer reasonable latents, since the inference uses both the model and the data. Although DSA and fixed-point search are offered as additional visualization tools, they require ground truth for evaluating whether the inferred dynamics are accurate.

5) The state R2 and cycle consistency metrics are linear, which is problematic. Any nonlinear change of variables in the latent dynamical system and its embedding yields an equivalent model. Linear state R2 only works because the embedding g(x) is fixed to be linear, eliminating nonlinear changes of variables. However, there is no reason to prefer linear embeddings. Nonlinear embeddings enable capturing dynamics on the manifold with the minimal number of latent dimensions [6]. Either modeling choice may be useful depending on the scientific question.

6) Injective mappings g(x) do not generally prevent learning of spurious features in the flow field, which arise in expressive models due to overfitting to noise [7]. Spurious features do not only result form excess dimensionality in the latent DD model, but arise also when the latent dimensionality matches the ground truth. The point that good data reconstruction does not ensure that a DD model identified the correct features was already noted in [7], which is not cited here despite raising this concerns prior to Ref. 46, 55. Moreover, [7] proposed a model selection approach based on cross-validation of model features instead of reconstruction metrics, without requiring ground truth, contrary to the statement here that all approaches need ground truth.

7) Scientific insight is expected to come from the flow field (Fig. 1E), but it is unclear how this insight will be derived in a more complex random target task, using any DD model. It would be helpful to clarify what is the ground truth flow field here. While this task is described as the most difficult, it needs to be clarified how it fits within the CtDB framework, where the ultimate goal is the dashed arrow in Fig. 1E. If it is fundamentally impossible to derive this insight even in TT model, it undermines the premise of fitting DD models to understand computation.

8) Describing the embedding g(x) as "implementation in a neural circuit" is misleading. The arbitrary (in this case linear) mapping g(x) bears no relationship to how the dynamics arise from the connectivity in a neural circuit. A more suitable term would be "implemented in neural activity", removing all references to circuits.

9) It is important to discuss the outcomes of NLB and its relationship to CtDB. Since this effort builds on NLB, it is critical to reflect on its specific successes and failures, as well as the broader challenges of benchmarking in neuroscience.

[1] https://www.nature.com/articles/s41593-021-00840-6

[2] https://www.nature.com/articles/s41586-024-08139-9

[3] https://www.nature.com/articles/s41593-019-0502-4

[4] https://www.science.org/doi/10.1126/science.aav7893

[5] https://www.nature.com/articles/nature25510

[6] https://www.biorxiv.org/content/10.1101/2023.07.22.550183v1

[7] https://www.nature.com/articles/s42256-020-00242-6

**********

**Have the authors made all data and (if applicable) computational code underlying the findings in their manuscript fully available?**

The PLOS Data policy requires authors to make all data and code underlying the findings described in their manuscript fully available without restriction, with rare exception (please refer to the Data Availability Statement in the manuscript PDF file). The data and code should be provided as part of the manuscript or its supporting information, or deposited to a public repository. For example, in addition to summary statistics, the data points behind means, medians and variance measures should be available. If there are restrictions on publicly sharing data or code —e.g. participant privacy or use of data from a third party—those must be specified.

Reviewer #1: Yes

Reviewer #2: Yes

**********

PLOS authors have the option to publish the peer review history of their article (what does this mean?). If published, this will include your full peer review and any attached files.

If you choose “no”, your identity will remain anonymous but your review may still be made public.

**Do you want your identity to be public for this peer review?**  For information about this choice, including consent withdrawal, please see our Privacy Policy.

Reviewer #1: No

Reviewer #2: No

[NOTE: If reviewer comments were submitted as an attachment file, they will be attached to this email and accessible via the submission site. Please log into your account, locate the manuscript record, and check for the action link "View Attachments". If this link does not appear, there are no attachment files.]

**Figure resubmission:**

While revising your submission, please upload your figure files to the Preflight Analysis and Conversion Engine (PACE) digital diagnostic tool, https://pacev2.apexcovantage.com/. PACE helps ensure that figures meet PLOS requirements. To use PACE, you must first register as a user. Registration is free. Then, login and navigate to the UPLOAD tab, where you will find detailed instructions on how to use the tool. If you encounter any issues or have any questions when using PACE, please email PLOS at figures@plos.org. Please note that Supporting Information files do not need this step. If there are other versions of figure files still present in your submission file inventory at resubmission, please replace them with the PACE-processed versions.

**Reproducibility:**

To enhance the reproducibility of your results, we recommend that authors of applicable studies deposit laboratory protocols in protocols.io, where a protocol can be assigned its own identifier (DOI) such that it can be cited independently in the future. Additionally, PLOS ONE offers an option to publish peer-reviewed clinical study protocols. Read more information on sharing protocols at https://plos.org/protocols?utm_medium=editorial-email&utm_source=authorletters&utm_campaign=protocols

# Author comment: Author response to Decision Letter 1

**DOI:** 10.1371/journal.pcbi.1014755.r002

28 Oct 2025

Attachment Submitted filename: Reviewer Response_CtDToolkit.docx

# Peer review: Decision Letter 1

**DOI:** 10.1371/journal.pcbi.1014755.r003

**Authors:** Abigail Morrison, Marieke Karlijn van Vugt

28 Jan 2026

PCOMPBIOL-D-25-00520R1

Computation-through-Dynamics Toolkit: Simulated datasets and quality metrics for dynamical models of neural activity

PLOS Computational Biology

Dear Dr. Versteeg,

Thank you for submitting your manuscript to PLOS Computational Biology. After careful consideration, we feel that it has merit but does not fully meet PLOS Computational Biology's publication criteria as it currently stands. Therefore, we invite you to submit a revised version of the manuscript that addresses the points raised during the review process.

Both the reviewers and I appreciate the considerable revisions that improve the clarity of your manuscript, and we are also all in agreement that the aim of your project is timely and important. However, the are some remaining issues that need to be taken care of to increase the impact of your work.

Please submit your revised manuscript by Mar 30 2026 11:59PM. If you will need more time than this to complete your revisions, please reply to this message or contact the journal office at ploscompbiol@plos.org. When you're ready to submit your revision, log on to https://www.editorialmanager.com/pcompbiol/ and select the 'Submissions Needing Revision' folder to locate your manuscript file.

Please include the following items when submitting your revised manuscript:

* A letter that responds to each point raised by the editor and reviewer(s). You should upload this letter as a separate file labeled 'Response to Reviewers'. This file does not need to include responses to formatting updates and technical items listed in the 'Journal Requirements' section below.

* A marked-up copy of your manuscript that highlights changes made to the original version. You should upload this as a separate file labeled 'Revised Manuscript with Track Changes'.

* An unmarked version of your revised paper without tracked changes. You should upload this as a separate file labeled 'Manuscript'.

If you would like to make changes to your financial disclosure, competing interests statement, or data availability statement, please make these updates within the submission form at the time of resubmission. Guidelines for resubmitting your figure files are available below the reviewer comments at the end of this letter

We look forward to receiving your revised manuscript.

Kind regards,

Abigail Morrison

Academic Editor

PLOS Computational Biology

Marieke van Vugt

Section Editor

PLOS Computational Biology

**Reviewers' comments:**

Reviewer's Responses to Questions

**Comments to the Authors:**

**Please note here if the review is uploaded as an attachment.**

Reviewer #1: This is a revised version of a previously submitted manuscript by Versteeg et al. on a toolbox combining benchmark tasks, datasets, and measures for assessing dynamical models of neural computation inferred from data.

I’m still generally in favor of this work and appreciate the initiative to create such a benchmark, as well as the thorough revision. I think the paper did improve at lot.

I still have a couple of concerns, however, some from the previous version that I still don’t find adequately addressed:

1) The authors added PhaseCodedMemory as an example with simple oscillatory activity, which is appreciated. Yet, there is still not a single example with chaotic activity, although there is mounting evidence that the brain operates in a chaotic regime (almost unavoidable for networks operating in a balanced regime, see for instance famous Nature paper by London et al. 2010). The authors argue that chaotic activity would not offer the reliability and robustness needed for neural information processing, but I disagree as chaotic attractors still have a reproducible statistical structure. They also argue that previous work testing models for inferring dynamical systems focused on poor proxies for dynamics in neural systems, namely mainly examples of 3d chaotic attractors. First of all, I think this misses the point a bit. While I fully agree that benchmarks closer to neuroscience are needed, I believe that for any method developed for DS identification it needs to be established first that it can indeed faithfully recover the dynamical structure of a variety of systems with different types of dynamical regimes and attractor geometries. And many of the papers cited which then aim to apply this to neuroscience often do go further and examine biologically more plausible scenarios or real data.

Second, the example benchmarks suggested by the authors are also all quite artificial. It may even be debatable whether and in which sense they are more realistic than a 3d chaotic attractor: Its chaotic dynamics may still be closer to the *dynamics* observed in the nervous system than a model just exhibiting fixed points (like 3BFF), i.e. constant firing rates in the limit. In fact, none of the benchmarks suggested were created from biophysically detailed or spiking neuron model simulations, they are all artificial high-level examples, and in that sense may not be physiologically realistic at all. The aspect that they perform computation on a task is important, however.

Bottom line: I still think the paper would benefit from inclusion of at least one chaotic example. There are many such neuro-examples out there, like in recent work of Brunel or older work by van Vreeswijk & Sompolinsky. I also think the phrasing needs to be more cautious in some places, in line with the points above, otherwise the paper sometimes sends a bit of a wrong message in my mind.

2) The authors suggest MMD instead of KL as a measure for assessing geometrical aspects of the dynamics, but I think this is based on a misunderstanding of how KL or Wasserstein distances are actually used in the literature. First, KL and Wasserstein are usually used as ground-truth *independent* measures, unlike how the authors introduce MMD in latent space which requires access to GT. What is commonly done here (see for instance recent review by Durstewitz et al. 2023 and refs. therein) is that a) the observations are delay embedded, b) the latent activity is projected into that same latent space via the used decoders. The problems of invariance to affine transformations or different dimensionalities thus do not occur the way these measures are commonly used on empirical data.

Second, KL and Wasserstein distances are well established and tested out in the dynamical systems lit., while MMD is not to my knowledge, and at least I personally have little intuition on how it behaves for different types of attractors and system dynamics. I therefore think for KL/Wasserstein one would be on safer ground with more experience from a lot of previous work. Alternatively or additionally, for temporal structure autocorrelation functions or power spectra are often used. Besides, the MMD would need a lot more explanation (what is \nu for instance?) and concrete examples that show how it behaves and scales for different degrees of geometrical overlap in DS settings.

3) I don’t quite understand the ‘non-injectivity’ argument, as it runs counter to the usual logic in DS theory where – just to the opposite of what the authors recommend – the representations or models are deliberately *expanded* (of much higher dimensionality) than the empirical data. This is the essence of the delay embedding theorems: We aim for a diffeomorphism between sets in the *true* underlying space in which the dynamics lives and the reconstructed space, and *not* literally between the latent space and the actual observations which usually only represent a tiny portion of all dynamical variables governing the observed system. Although the issue of models introducing spurious features is certainly a very important one, and although the general idea may be correct that over-parameterized and more complex models are more likely to introduce such features, the logic of the injection principle as described and specifically implemented in the paper in my mind is somewhat flawed. I suspect that it only works for the kind of toy models the authors examine with access to *all* dynamical variables, but, as stated in the paper, would break down on empirical data.

I don’t want this to come across too negative, I in fact appreciate the authors’ efforts to introduce a common standard or basis into the field, and one certainly needs to make a start somewhere! But I find the points above important, and I hope the authors will see what I mean and could consider in their revision one chaotic example and possibly measures better established in the DS identification literature.

Minor points:

- The authors expanded their f:I→O definition of computation. What this definition still misses in my mind is a notion of internal states as crucial in most concepts of computation like Turing machines or finite state machines. Left up to the authors, just wanted to mention this.

- I still would discourage the use of the work “embedding” for a projection from the latent into the neuronal space, as this conflicts with common usage in ML and DS. How about “neural encoding” or “representation” or similar?

- Since so much in this article is viewed in terms of dynamics, I still find it a bit of a pity that it interfaces so little with the rich literature on dynamical systems reconstruction.

- I’m not sure the term “under-fitting” describes best the issue LDS faces on many data: They simply cannot represent the dynamics at all, regardless of how many parameters.

- For me the LDS results still suggest that RandTarget is the simplest, not the most complex of the benchmarks introduced, perhaps only requiring linear regression from inputs to outputs?

- Not sure Fig. 6E is topologically possible: If the map defining the underlying DS is smooth and invertible (and since chaos is absent here), there always would need to be saddles separating the fixed points (Smale-Morse).

In some places I missed a bit more precision in the terminology, for example:

- p.12: Fixed points are mathematically defined as points where the flow completely vanishes (is *exactly* zero), not when it is “sufficiently slow”. And the linear “approximation” (actually it is a topological equivalence) holds only for hyperbolic systems for a sufficiently small environment around the fixed point (not related to “slowness”).

- p.12/13: The overall stability properties of the system are not described by a single Lyapunov exponent but by the whole Lyapunov spectrum (which can also easily be computed from the Jacobians). For instance, a chaotic set can still be an attracting set if the sum of all Lyapunov exponents is smaller than 0. The virtue of the *maximal* Lyapunov exponent is that this can usually also be assessed from real data, hence directly compared and used as quality stats.

Reviewer #2: I thank the authors for the careful revisions of the manuscript, which have addressed most of the confusion points raised in the previous review. In particular, the goals of the CtDToolkit as a platform for model development and testing are now clear. The current manuscript is also more explicit about the fundamental ambiguity in attributing the observed activity to dynamics versus inputs. These revisions substantially improve the framing of the CtDToolkit and its grounding in neuroscience and the broader dynamical-systems inference literature. However, several issues have not been addressed in full and require further clarifications, as detailed in the specific comments below. Addressing these issues is important, as CtDToolkit is intended to become a widely used tool, aimed at consolidating the field around common standards of model evaluation. Therefore, it is critical to ensure that the logic and philosophy guiding such model evaluation are sound and capable of fostering consensus within the community.

Major comments.

1. The paper is inconsistent about the purpose of the evaluation metrics. The text states at multiple points that these metrics should "guide model selection". In other places, it states that these metrics are used only for "post-hoc assessment of previously trained models", contrasting it with the model selection approach from Ref. [38]. This distinction is confusing. What is the purpose of "post-hoc assessment" if not to select the optimal model for a specific dataset? Indeed, the description of the application of these metrics to example datasets (Figs. 5,6) reads exactly as a model selection procedure: some models are underfitted, some are overfitted, and some are just right (according to the metrics). How is the "post-hoc assessment" different from model selection?

2. The paper is inconsistent about the description of hyperparameter tuning. Returning to the stated contrast between "post-hoc assessment" and what is described in the manuscript as "training-phase interventions" in the context of Ref. [38], this framing is misleading, as both approaches are model selection procedures aimed at identifying hyperparameters that yield models most closely matching the true dynamics. For sufficiently expressive DD models, model selection amounts to choosing optimal hyperparameters: latent state dimensionality and regularization strength. The manuscript illustrates how the evaluation metrics can be applied to select both types of hyperparameters: latent state dimensionality (Fig. 5) and regularization strength on inputs (Fig. 6). Similarly, the model selection procedure in Ref. [38] concerns with choosing the regularization strength on model complexity: a training epoch for early stopping or the weight of model complexity penalty. Expressive models trained via gradient descent learn features of increasing complexity across training epochs. Therefore, the number of training epochs is a critical hyperparameter that can result in under- or overfitting. However, the description of results in Fig. 5 glosses over this issue: all models were trained for a fixed number of epochs chosen ad hoc and this choice is not discussed, making a misleading impression that the latent state dimensionality is the only important hyperparameter. To be clear, I do not suggest to implement the model selection metrics from Ref. [38] within the CtDToolkit nor to change the results in Fig. 5. Rather, I encourage the authors to describe the problem of hyperparameter selection consistently, holistically, and transparently. Since CtDToolkit aims at a broad audience of model developers with varying amount of expertise, who may not be able to read between the lines, it is important to avoid misleading distinctions, such as between training-phase versus post-hoc assessment, and to be upfront about all different kinds of hyperparameters critical for identifying correct dynamics with expressive models.

3. As mentioned in the previous review, injective mappings do not generally prevent inferring spurious features in the dynamics (shown in Ref. [38]). While I agree with the authors' response that an injective mapping is preferred over a degenerate one, the insufficiency of injective mappings has not been addressed in the manuscript. To the contrary, the text suggests at multiple points that injective mappings are sufficient (e.g., statements like "Alone, reconstruction and simplicity are each insufficient to judge DD model quality, but together they can provide interpretable accounts of DD model performance when external inputs are known" or the last paragraph on page 11). The issue with this argument is that it reasons about "predicted neural activity" missing the distinction between firing rates and spikes. Spurious features in latents can be predicted from firing rates if the mapping is injective, but these features may not be detected by reconstruction metrics defined on spikes. There may be indications of this effect in Fig. 5E,G,H. For NODE-64, state R2 appears much lower than cycle consistency, suggesting that inferred latents have features predictable from rates but not present in the true latents, without affecting reconstruction metrics (rate R2 and co-BPS). Since both rates and latents are unobserved quantities, their consistency does not generally guarantee the correctness of the inferred dynamics.

4. Related to the previous point, the true rates in Fig. 5B exhibit high-frequency features. What is the source of these features? Do they arise from noise injected in the TT models? If so, a model capable of predicting random fluctuations should be considered overfitted. While NODE-8 clearly predicts the high-frequency features better than NODE-3 (as confirmed by the rate R2), are these predictions meaningful if they reflect random noise rather than task-relevant dynamics? Since NODE does not model stochastic dynamics (i.e. the distribution over latent trajectories on each trial), it may be especially prone to this type of overfitting. This raises a question of whether the rate R2 may be contaminated by the reconstruction of noise. If so, it becomes unclear how much improvement in reconstruction (along the y-axis in Fig. 5I) do we actually want from the optimal model? In other words, what direct evidence supports that NODE-3 is underfitting rather than NODE-8 overfitting to noise in the TT model rates?

5. There are several technical issues In Fig. 5 related to previous points. (i) Fig. 5F shows that the state R2 and cycle consistency have nearly identical values across models (points on the diagonal), whereas Figs. 5G,H make it clear that cycle consistency is systematically higher than state R2 across models. Which data are correct? They cannot be correct simultaneously. (ii) In Fig. 5E, the prediction from the true latents for NODE-64 appears to show a systematic bias (the two time series have different means), which is unexpected for an affine transformation. What is the issue here? (iii) What are the ticks at the bottom of Fig. 5B? What are the time units here relative to the timescale of the task? (iv) In Fig. 5C,F,G,H it would be helpful to indicate all models of the same size with the same color, and highlight the models shown in panels B,E with a distinct symbol. In the current presentation, it is hard to assess the spread across models of the same size.

6. Many of the issues raised above stem from the fact that all metrics are computed on latents and rates, rather than directly assessing the accuracy of reconstructing the ground-truth flow field. Any error metric between the inferred and ground-truth flow fields would not suffer from problems such as overfitting to noise in the true rates or Poisson noise. While such a metric is not trivial to compute for the TT model architectures chosen here, it would be relatively straightforward for other architectures, e.g., low-rank RNNs. To be clear, I do not suggest to introduce additional TT model architectures in the CtDToolkit. It would be sufficient to acknowledge that many problems arise due to the lack of direct comparison with the ground-truth flow field, which can be addressed in the future using different TT model architectures.

7. The need for co-smoothing metric is specific to the DD model architectures considered here, which are based on sequential autoencoders that require access to neural data to make predictions even in validation and testing. For DD models that do not require spike data as input, a likelihood computed across all neurons on held-out trials is sufficient to assess the prediction accuracy and should be preferred over co-smoothing. Although co-smoothing is necessary to prevent autoencoders from learning trivial identity maps, this metric uses less data for training and testing and hence is noisier, and it is also sensitive to which neurons will land in the held-in and held-out sets. Since CtDToolkit targets a broad model-development community, this issue should be acknowledged because not all DD models are autoencoders.

8. The previously raised concern about the assumption of linear embeddings has not been addressed in full. Several related issues remain.

8.1. The fact that all experiments use linear mapping between latents and log-rates should be stated upfront in the main text (as in the original submission), as this became less apparent in the revised manuscript.

8.2. The fact that nonlinear cycle consistency yields similar results to linear cycle consistency under the canonical embedding (Fig. S8) does not validate that nonlinear cycle consistency produces good results under nonlinear embeddings. What is the purpose of this analysis and has the nonlinear cycle consistency been validated under nonlinear embeddings?

8.3. The nonlinear cycle consistency is based on fitting MLP, which is an expressive model with many hyperparameters (I couldn't find these hyperparameters in the paper). How are these hyperparameters tuned for each model? How do we know that the loss of robustness to noise arises from a non-injective embedding rather than from a brittle, overfitted MLP?

9. The concern about the use of linear metrics remains, as it also applies to state R2. Any nonlinear change of variables in the latent dynamical system and its embedding yields an equivalent model. Linear state R2 works in the example applications here because the embedding is fixed to be linear, eliminating nonlinear changes of variables. This issue has not been addressed in revision or response letter.

10. The description of data generation from TT models is unclear. "The activity of each of these sampled units is standardized by subtracting the mean of each unit individually, and then normalizing by the scaled standard deviation across all units". What is "scaled standard deviation across all units"? Was activity of all units normalized by the same number or different numbers? Does this mean each unit's activity was z-scored? Since firing rates in neural recordings are heterogeneous, what is the rationale for z-scoring the activity of all units? What is the purpose of the fr_scaling parameter?

11. The seemingly innocuous latent z-scoring prior to data generation may introduce artifacts, such as an apparent failure of the DD model on the PhaseCodedMemory dataset. If the TT model contains some units with low variance and small differences in mean activity between two conditions, these units contribute negligibly to the total variance, such that the shift between the two ring centroids is virtually invisible in PCs of the true latents (Fig. S3D). These low-variance units may also contribute negligibly to the TT model output, showing low-variance fluctuations with minimal task modulation. However, after z-scoring, their contribution will be up-weighted while the contribution of task-modulated high-variance units will be down-weighted, which will amplify the shift between the ring centroids in z-scored latents. As a result, DD models trained on the scaled latents will also exhibit a shift between the ring centroids. This scenario should be regarded as a failure of data generation (or comparison with wrong latents), not a failure of the DD model. Although it is hard to tell from the text whether this scenario actually takes place, as the description of the results and analyses is incomplete, it is plausible. The TT model has 512 units and is trained with an L2 penalty on the latents, making it plausible that the model contains many low-variance, task-irrelevant units. In addition, the numbers reported in the text appear inconsistent: Table S10 indicates that the dataset includes 500 held-in and 100 held-out units, whereas the TT model contains only 512 units.

12. Overall, the presentation of the PhseCodedMemory task is underwhelming. The conclusion that it "provides a striking demonstration of how naively trusting dynamics learned by a DD model could lead researchers to incorrect conclusions" is vague and uninformative in the absence of any performance metrics. What does it mean to "naively trust" the dynamics? Not using any performance metrics at all? Without any intuition about the mechanism causing the DD model failure, this result is also impossible to interpret.

13. I agree that current TT models are task-computation focused, but referring to them in the text as "biologically plausible" is misleading. These GRU architectures with tanh activation do not compute through biologically-plausible non-negative firing rates. The firing rates are obtained separately through additional transformations of the GRU activity. It would be appropriate to remove all references to the biological plausibility of these models.

14. The results in Fig. S7 are unclear and their description is confusing and incomplete. Fig. 5E indicates the NODE infers the correct number of stable fixed points, whereas GRU infers many more fixed points than in the TT model. Nevertheless, the DSA metric indicates that the GRU is closer to the true model than NODE. What is the source of the apparent discrepancy here? Section E.3.1. is cryptic and impossible to understand.

15. In response to the suggestion to comment on the outcomes of the NLB, the authors added a paragraph stating that users tended to focus on a small subset of datasets. This response, however, does not address the intent of the original question. It would be more informative to discuss the broader impact of the NLB on the modeling community, including how it influenced modeling practices across the field, which aspects of the NLB design were successful, and which aspects proved wrong. Such a discussion could naturally continue with an outlook on the impact the authors envision the CtDToolkit will have on the community.

Minor comments.

1. The text is often unclear about what is meant by "dynamics", whether it refers to the flow field or latents. The two are not equivalent, and at several places the text seems to conflate latents with the flow field.

2. The main text should state explicitly that DSA requires access to the true latents.

3. What is the definition of simple inputs (Fig. 2D)? How is the input simplicity measured?

4. The problem of determining the dimensionality of inputs is not mentioned.

5. Fig. 1G is confusing. If trajectory moves to the fixed point B, what happens as the input sequence continues? It seems this illustration works only if we consider a short trial with two input pulses, but it is unclear how we should understand the invented feature for a longer input sequence.

6. Ref. [38] does not use random initializations: all models are initialized at the minimum of model complexity.

7. "This suggests that not only is reconstruction insufficient for DD model selection, but relying on reconstruction alone may be actively misleading!" While the exclamation mark makes it sound like a revelation, this fact has long been established (see Ref. [38]). It would be appropriate to remove the exclamation mark and cite Ref. [38] in this sentence instead.

8. "In Supp. Fig. S7, we show that DSA may be able to ignore such trivial non-linearities. However, it is still unclear whether these geometric differences can be safely ignored". Recent work shows that such geometric differences may indicate distinct task solutions and thus cannot be ignored (https://www.nature.com/articles/s42256-025-01127-2).

9. The description of models in Appendix is incomplete, many details are missing, making it impossible to understand. Since this paper is intended as a manual for a broad community of users, the authors should put a substantial effort in improving the methods sections in Appendix. Here are some examples of incomplete/confusing descriptions.

9.1. 3BFF task: what is x in the Loss function? What are x, U, B_h, b_z in Eqs. 9-13? Which of them are trainable parameters, inputs, and output? It should be briefly stated.

9.2. How does GRU DD model map latents to firing rates?

9.3. How b_h is different from b_x in Eq. 14?

9.4. The text describes SLDS, but it is not included in the toolkit and no results are shown, so it should be removed from the text.

9.5. What is the time unit of the simulation? Do TT and DD models use the same time step?

**********

**Have the authors made all data and (if applicable) computational code underlying the findings in their manuscript fully available?**

The PLOS Data policy requires authors to make all data and code underlying the findings described in their manuscript fully available without restriction, with rare exception (please refer to the Data Availability Statement in the manuscript PDF file). The data and code should be provided as part of the manuscript or its supporting information, or deposited to a public repository. For example, in addition to summary statistics, the data points behind means, medians and variance measures should be available. If there are restrictions on publicly sharing data or code —e.g. participant privacy or use of data from a third party—those must be specified.

Reviewer #1: None

Reviewer #2: Yes

**********

PLOS authors have the option to publish the peer review history of their article (what does this mean?). If published, this will include your full peer review and any attached files.

If you choose “no”, your identity will remain anonymous but your review may still be made public.

**Do you want your identity to be public for this peer review?**  For information about this choice, including consent withdrawal, please see our Privacy Policy.

Reviewer #1: No

Reviewer #2: **Yes:**  Tatiana Engel

[NOTE: If reviewer comments were submitted as an attachment file, they will be attached to this email and accessible via the submission site. Please log into your account, locate the manuscript record, and check for the action link "View Attachments". If this link does not appear, there are no attachment files.]

**Figure resubmission:**

**Reproducibility:**

?>

# Author comment: Author response to Decision Letter 2

**DOI:** 10.1371/journal.pcbi.1014755.r004

31 May 2026

Attachment Submitted filename: response_to_reviewers.pdf

# Peer review: Decision Letter 2

**DOI:** 10.1371/journal.pcbi.1014755.r005

**Authors:** Abigail Morrison, Marieke Karlijn van Vugt

23 Aug 2026

Dear Dr. Versteeg,

We are pleased to inform you that your manuscript 'Computation-through-Dynamics Toolkit: Simulated datasets and quality metrics for dynamical models of neural activity' has been provisionally accepted for publication in PLOS Computational Biology.

Before your manuscript can be formally accepted you will need to complete some formatting changes, which you will receive in a follow up email. A member of our team will be in touch with a set of requests.

Please note that your manuscript will not be scheduled for publication until you have made the required changes, so a swift response is appreciated.

IMPORTANT: The editorial review process is now complete. PLOS will only permit corrections to spelling, formatting or significant scientific errors from this point onwards. Requests for major changes, or any which affect the scientific understanding of your work, will cause delays to the publication date of your manuscript.

Should you, your institution's press office or the journal office choose to press release your paper, you will automatically be opted out of early publication. We ask that you notify us now if you or your institution is planning to press release the article. All press must be co-ordinated with PLOS.

Thank you again for supporting Open Access publishing; we are looking forward to publishing your work in PLOS Computational Biology.

Best regards,

Abigail Morrison

Academic Editor

PLOS Computational Biology

Marieke van Vugt

Section Editor

PLOS Computational Biology

***********************************************************

Reviewer's Responses to Questions

**Comments to the Authors:**

**Please note here if the review is uploaded as an attachment.**

Reviewer #1: I thank the authors for their careful revisions, and for taking my comments so seriously!

I am generally happy with this second revision as it stands now, and think this it is largely ready for publication. I think it is important that this toolbox will be an open environment that allows other researchers to contribute further benchmark models and evaluation statistics. In this sense, one may want to see the authors’ contribution more as a start, which can further grow and diversify with input from the wider research community (and my understanding from the Intro is that’s the way the authors actually conceived it).

I have a few remaining comments, but leave it up to the authors whether they want to address them:

1) In reply to my first comment the authors argue that they wanted to put the emphasis on models which implement actual tasks, and therefore chose simpler, RNN-based models. However, there is by now also a large variety of task-trained spiking neural networks (with the advance of gradient descent techniques that work for spiking neurons), or biophysically more realistic SNNs trained by other learning rules as in recent work by Brunel, which exhibit both chaotic dynamics and computational features like working memory. Personally, for neuroscience I still find these more appealing benchmarks as they are closer to biology, but it’s a future extension the authors may just want to discuss.

I also still find the phrasing in the subsection “Synthetic systems as proxies for neural circuits” a bit subjective and reflecting more the authors’ personal opinions rather than scientifically supported evidence. For instance, “chaotic attractors don’t "do" anything” – sure, but that’s true for *any* attractor or dynamical system that has not been trained to perform a task, it is not specific to chaos. And many of the tasks are also low-dimensional (see tiny-RNN Nature paper 2025); vice versa, in the literature referred to by the authors there are often also examples of high-d systems. That is, the text appears a bit imbalanced to me here, and I think it suffices to say that most of the DS usually considered in the DSR literature a) do not receive (much) external inputs (essential in the nervous system), and b) are not trained to perform any computations. This in itself is a strong argument for the authors’ approach!

2) It’s great the authors added the Wasserstein distance to the repertoire of evaluation measures!

Let me say that KL in my perception is the more commonly used measure, and has some advantages over Wasserstein, esp. if Wasserstein-1 is used which can obscure some of the geometrical structure through the projections. Something to be aware of.

3) I agree with the authors that “tangled trajectories” may also reflect external inputs, but that rather further supports the point for expanding the space as in non-autonomous systems in my opinion. In my mind this is not “inadvertently shifting the object of study” (as the authors write) but simply what one would need to do if one wants to stand any chance of recovering the true attractor objects (unless you can explicitly model the external inputs as well). But I appreciate the authors have softened the phrasing in this section; I think it’s important to be aware of the pro’s and con’s and potential pitfalls of the choices one makes here.

Again, these are just remaining thoughts, and I completely leave it up to the authors whether they want to implement any further changes. They certainly already put a lot of time and effort into this!

Reviewer #2: The authors have excellently addressed all remaining points raised in the previous round of review. The revised manuscript is conceptually coherent, clearly written, and presents accurate and rigorous results. It also strikes an appropriate balance between highlighting advances in the field and acknowledging important limitations that remain to be addressed in future work. Overall, the manuscript is excellent, and, together with the accompanying codebase, it will undoubtedly become a highly valuable and widely used resource for the community interested in modeling neural dynamics.

While reading the paper, I noticed a few minor inconsistencies in notation, pointed out below. These issues can be easily corrected by the authors without the need for another round of review.

Small issues.

1. There is some inconsistency in what is defined as an observation model g(z). On Fig. 1B (lower panel), it is defined as n=g(z), where n are the firing rates. On page 5 first line, it is stated that y=g(z), where y is the observed neural spiking activity.

2. In Eq. 1, the rates are denoted with N instead of n.

3. In section 2.4.1, where Rate R^2, State R^2, and Input R^2 are introduced, it would be helpful to briefly explain the rationale for predicting the ground-truth quantities from the inferred quantities for Rate R^2 and Input R^2, but using the reverse direction for State R^2.

4. The \hat notation is used to indicate model-inferred features. However, in the equation for co-bps (which is right after Eq. 3) and in the text following this equation, \hat{y} is used to denote the observed spike counts instead of inferred quantity.

**********

**Have the authors made all data and (if applicable) computational code underlying the findings in their manuscript fully available?**

The PLOS Data policy requires authors to make all data and code underlying the findings described in their manuscript fully available without restriction, with rare exception (please refer to the Data Availability Statement in the manuscript PDF file). The data and code should be provided as part of the manuscript or its supporting information, or deposited to a public repository. For example, in addition to summary statistics, the data points behind means, medians and variance measures should be available. If there are restrictions on publicly sharing data or code —e.g. participant privacy or use of data from a third party—those must be specified.

Reviewer #1: Yes

Reviewer #2: Yes

**********

PLOS authors have the option to publish the peer review history of their article (what does this mean?). If published, this will include your full peer review and any attached files.

If you choose “no”, your identity will remain anonymous but your review may still be made public.

**Do you want your identity to be public for this peer review?**  For information about this choice, including consent withdrawal, please see our Privacy Policy.

Reviewer #1: No

Reviewer #2: **Yes:**  Tatiana Engel

# Editor report: Acceptance letter

**DOI:** 10.1371/journal.pcbi.1014755.r006

**Authors:** Abigail Morrison, Marieke Karlijn van Vugt

PCOMPBIOL-D-25-00520R2

Computation-through-Dynamics Toolkit: Simulated datasets and quality metrics for dynamical models of neural activity

Dear Dr Versteeg,

I am pleased to inform you that your manuscript has been formally accepted for publication in PLOS Computational Biology. Your manuscript is now with our production department and you will be notified of the publication date in due course.

The corresponding author will soon be receiving a typeset proof for review, to ensure errors have not been introduced during production. Please review the PDF proof of your manuscript carefully, as this is the last chance to correct any errors. Please note that major changes, or those which affect the scientific understanding of the work, will likely cause delays to the publication date of your manuscript.

Soon after your final files are uploaded, unless you have opted out, the early version of your manuscript will be published online. The date of the early version will be your article's publication date. The final article will be published to the same URL, and all versions of the paper will be accessible to readers.

For Research, Software, and Methods articles, you will receive an invoice from PLOS for your publication fee after your manuscript has reached the completed accept phase. If you receive an email requesting payment before acceptance or for any other service, this may be a phishing scheme. Learn how to identify phishing emails and protect your accounts at https://explore.plos.org/phishing.

Thank you again for supporting PLOS Computational Biology and open-access publishing. We are looking forward to publishing your work!

With kind regards,

Sharmila Kamatchi

PLOS Computational Biology | Carlyle House, Carlyle Road, Cambridge CB4 3DN | United Kingdom ploscompbiol@plos.org | Phone +44 (0) 1223-442824 | ploscompbiol.org | @PLOSCompBiol
